# A novel spirocyclic scaffold accessed via tandem Claisen rearrangement/intramolecular oxa-Michael addition

**DOI:** 10.3762/bjoc.18.177

**Published:** 2022-12-06

**Authors:** Anastasia Vepreva, Alexander Yanovich, Dmitry Dar’in, Grigory Kantin, Alexander Bunev, Mikhail Krasavin

**Affiliations:** 1 Saint Petersburg State University, Saint Petersburg 199034, Russian Federation; 2 Academic Gymnasium of Saint Petersburg University, Saint Petersburg 199155, Russian Federation; 3 Medicinal Chemistry Center, Togliatti State University, Togliatti 445020, Russian Federation; 4 Immanuel Kant Baltic Federal University, Kaliningrad 236016, Russian Federation

**Keywords:** Claisen rearrangement, diazo arylidene succinimides, intramolecular oxa-Michael addition, rhodium(II) carbene O–H insertion, spirocycles

## Abstract

A straightforward access to novel spiro[benzofuran-2,3'-pyrrolidine]-2',5'-diones based on the Rh_2_(esp)_2_-catalyzed insertion of carbenes derived from diazo arylidene succinimides (DAS) into the O–H bond of phenols is described. The initial adducts underwent a thermally promoted Claisen rearrangement followed by DABCO-catalyzed intramolecular 5-*exo*-*trig* oxa-Michael addition.

## Introduction

**Figure 1:**
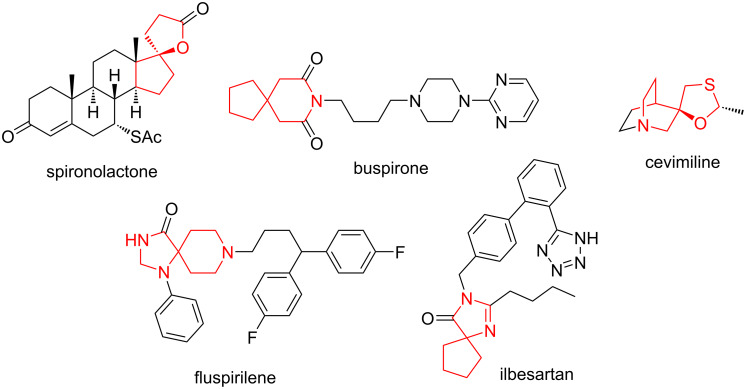
Examples of approved spirocyclic drugs.

Spirocycles undoubtedly occupy a special place in drug design [1] and, in general, spirocyclic compounds intended for the interrogation of biological targets have been associated with higher success rates [2] in discovering new cases of affinity towards a three-dimensional protein molecule [3]. Spirocycles of all sorts are omnipresent in the natural product realm [4]. Among the approved medicines the following spirocyclic molecules are notable: spironolactone for heart disease and hypertention [5], buspirone for anxiety disorders [6], cevimeline for dry mouth and dry eye syndrome [7], fluspirilene for schizophrenia [8] and ilbesartan for hypertention and diabetic nephropathy [9], to mention a few (Figure 1).

Hence the development of novel synthetic methods to construct spirocycles [10–14] constitutes a distinctly worthy undertaking which may as well influence the outlook of the novel medicines discovered and developed in the future.

**Scheme 1:**
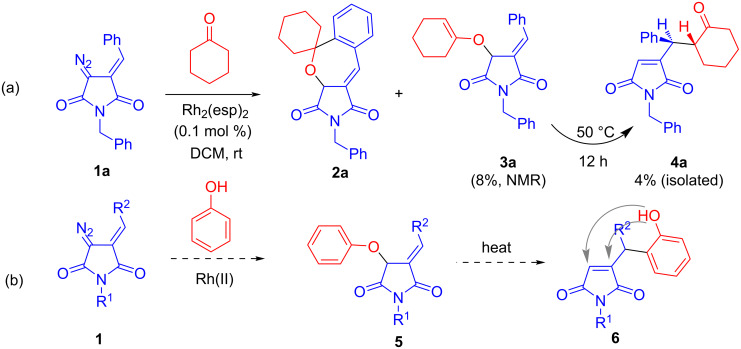
(a) Earlier reported Rh(II)-catalyzed spirocyclization of DAS with the formation of minor enol ether product **3a** and its Claisen rearrangement. (b) Synthetic strategy investigated in this work.

Recently, we described a novel Rh(II)-catalyzed spirocyclizations involving diazo arylidene succinimide (DAS, such as **1a**) with cyclohexanone (as well as other cyclic ketones) which delivered spiro-annulated 2-benzoxepines (such as **2a**) along with a minor byproduct **3a** identified by ^1^H NMR as the product of formal insertion of the rhodium(II) carbene species into the O–H bond of cyclohexanone enol form. This minor byproduct, on heating at 50 °C for 12 h, underwent the Claisen rearrangement to give diastereomerically pure maleimide **4a** in 4% yield [15]. While for our study at the time the formation of **3** and **4** were viewed as a minor side-reaction, later be started pondering the possibility of giving the observed transformation a stronger impetus from the synthetic point of view. Specifically, we wanted to see if Rh(II)-catalyzed insertion of DAS-derived carbenes could be performed into the O–H bond of phenols and if the resulting phenoxy-substituted succinimides **5** could also undergo a Claisen rearrangement. The products of the latter (**6**) would again have a reactive phenolic hydroxy group which could potentially be involved in post-condensational transformations such as intramolecular oxa-Michael addition (Scheme 1). Herein, we report our findings obtained in the course of investigating the viability of this strategy.

## Results and Discussion

**Scheme 2:**
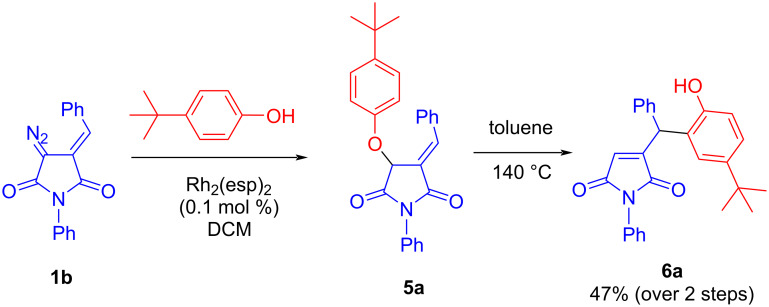
Initial attempt at Rh(II)-catalyzed O–H insertion/Claisen rearrangement.

The initial attempt to involve DAS **1b** in the Rh_2_(esp)_2_-catalyzed insertion reaction with 4-(*tert*-butyl)phenol was successful. The initial adduct **5b** was not purified and was heated at 140 °C in toluene to give compound **6b** in 47% yield over two steps (Scheme 2). No further optimization of the reaction conditions was undertaken except for the isolation for the initial O–H insertion product which facilitated subsequent steps (the Claisen rearrangement and the final cyclization, vide infra).

With product **6a** at hand we proceeded studying its base-promoted Michael-type cyclizations. As it follows from the data collated in Table 1, to our delight, cyclizations of the phenoxide anion generated on the action of base (except for 2,6-lutidine) proceeded as 5-*exo-trig* (rather than 6-*endo-trig*) process and yielded spirocyclic compound **7a** as a mixture of *syn* and *anti* diastereomers. DABCO in toluene at room temperature over 1 h (Table 1, entry 5) gave a superior result both in terms of the isolated yield and diastereoselectivity. Increasing the polarity of the solvent appeared to be detrimental to the reaction outcome.

**Table 1:** Investigation of base-catalyzed transformation of compound **6a**.

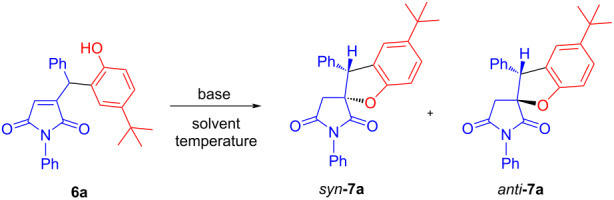						

Entry	Solvent	Base (30 mol %)	*T* (°C)	Total yield (%)	*syn*/*anti*	Conversion within 1 h (%)

1	toluene	DABCO	0	34	79:21	70
2	toluene	DBU	25	63	62:38	100
3	toluene	Cs_2_CO_3_	25	66	73:27	100
4	toluene	2,6-lutidine	25	–	–	–
5	toluene	DABCO	25	**71**	**81:19**	100
6	acetonitrile	DABCO	25	66	77:23	100
7	methanol	DABCO	25	48	46:54	100
8	MeOH/H_2_O (1:1)	DABCO	25	–	–	–

With the conditions identified for the Rh_2_(esp)_2_-catalyzed insertion into the phenolic O–H bond, the Claisen rearrangement and the base-catalyzed cyclization to the spirocyclic product, we experimented with attempts to perform all three steps in a one-pot format. This gave inferior results in terms of product yield and purity. The best result was obtained by performing the Rh(II)-catalyzed insertion first, purifying the respective product **5** and then engaging it in the sequential Claisen rearrangement cyclization reactions performed in one-pot format.

**Scheme 3:**
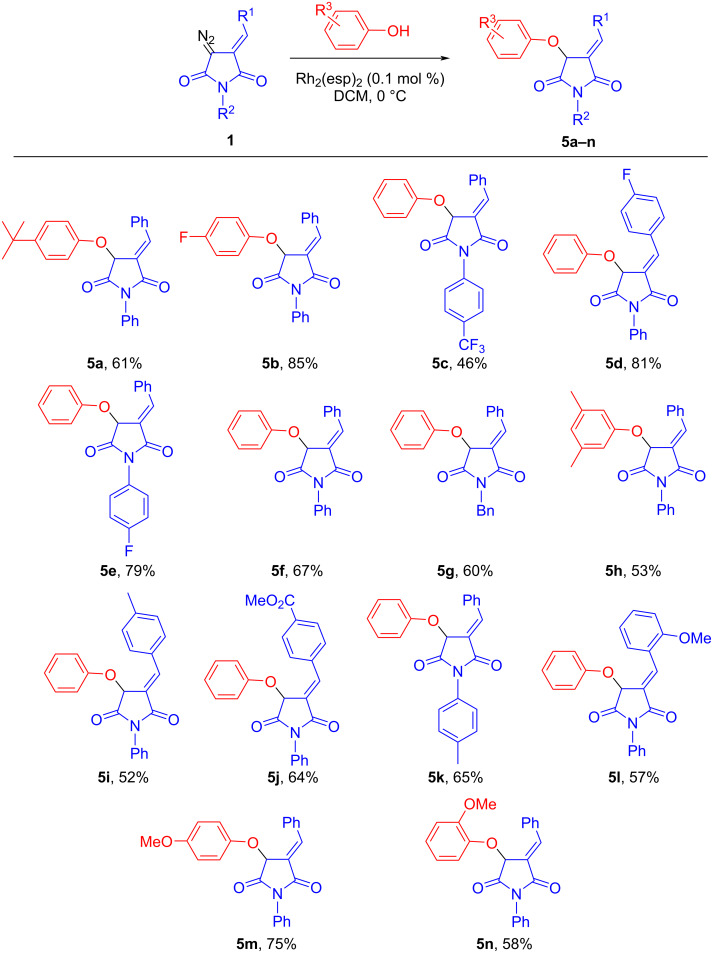
Rh_2_(esp)_2_-catalyzed O–H insertion reactions between various DAS **1** and phenols.

To expand the scope of the newly discovered transformations, several products of the Rh(II)-catalyzed O–H insertions **5** were synthesized as detailed in Scheme 3.

The O–H insertion reaction worked well for electron-neutral or electron-rich phenols. The presence of chlorine substituents (such as 4-Cl or 2,4-diCl) in the phenol component drastically diminished the yield and led to the formation of the earlier reported DAS dimer [16]. Similarly, electron-donating groups (such as 4-methoxy) in the arylidene portion of **1** complicated the course of the reaction.

**Scheme 4:**
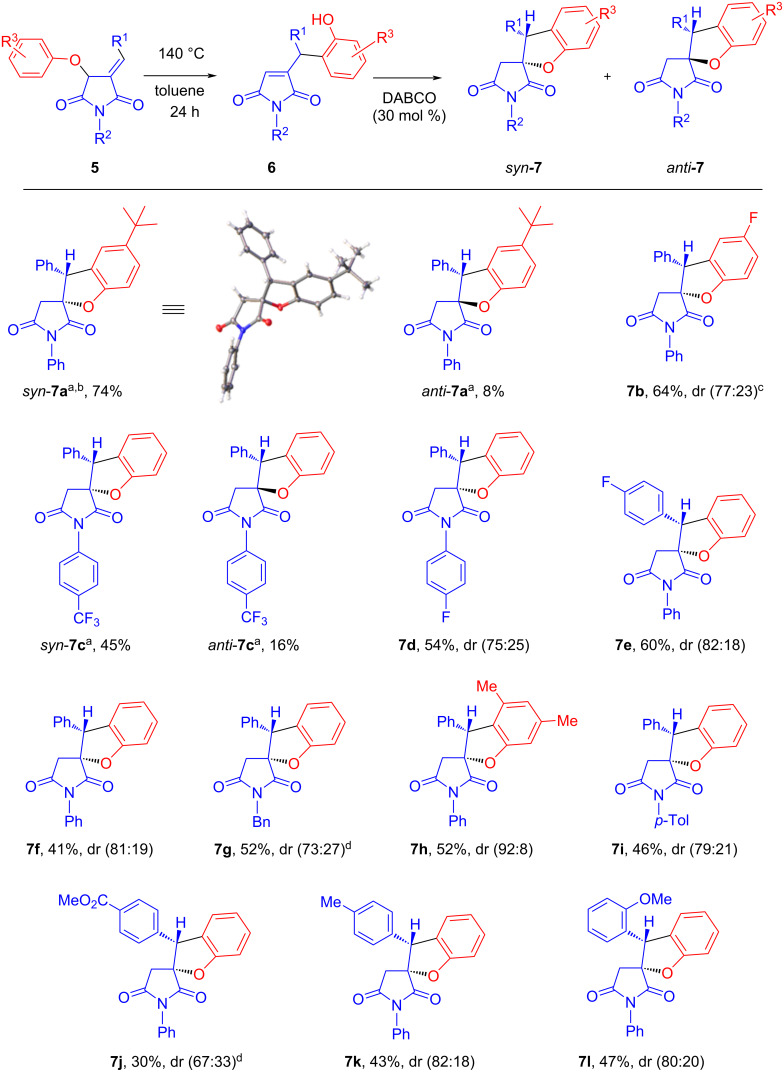
Two-step, one-pot sequence of the Claisen rearrangement/intramolecular Michael-type spirocyclization of substrates **5a–l**. The Claisen rearrangement product was not isolated. Scale from 0.3 to 0.9 mmol; pure major *syn* diastereomer was isolated and characterized in all cases; ^a^in these examples, pure minor *anti* diastereomer was isolated and characterized; ^b^the structure was confirmed by crystallography. ^c^*syn*/*anti* ratio is shown in parentheses; ^d^the reaction was performed at 150 °C.

Having amassed a sizable arsenal of O–H insertion products **5a–n**, we proceeded to study their behavior in the two-step, one-pot sequence of the Claisen rearrangement/intramolecular Michael-type spirocyclization as detailed in Scheme 4. In two cases (**7a** and **7c**), the major (*syn*) and minor (*anti*) diastereomers were separated chromatographically and characterized. In one case (**7a**), the structure of the major (*syn*) diastereomer was unequivocally confirmed by single-crystal X-ray crystallography. In all other cases, only the pure *syn* diastereomer was isolated and characterized. The yields of spirocyclic products were generally modest to good over two steps. An electron-accepting group in the benzylidene portion (**5j**) or an *N*-benzyl substitution in the starting material (**5g**) lowered the reactivity and the Claisen rearrangement step was performed at a higher (150 °C) temperature.

Notable was our inability to involve *o*-methoxy- (**5n**) and (*p*-methoxy)phenoxy (**5m**) substrates in the two-step synthesis of the respective spirocycles **7**. In both cases, ^1^H NMR analysis of the reaction mixture indicated the formation of complex product mixtures at the Claisen rearrangement step.

**Scheme 5:**
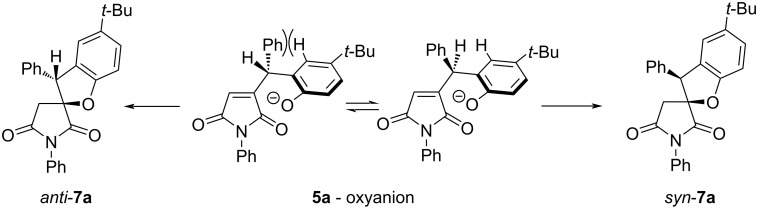
Tentative rationalization of the diastereoselectivity observed in all **5**→**7** transformations (shown for **5a**→**7a**).

3,5-Dimethylphenoxy-substituted substrate **5h** gave the best diastereomeric ratio in the series. The preference for the formation of the *syn* diastereomer in each case (and for **5a**→**7a** in particular) can, in principle, be rationalized by the greater steric repulsion in the conformer of the respective oxyanion leading to the *anti* diastereomer compared to that from which the major, *syn* diastereomer is formed (Scheme 5).

Notably, a compound analogous to **5a** was synthesized with thiophenol in 78% yield. However, it turned out to be completely unreactive towards the Claisen rearrangement step, even at 150 °C in 1,2-dichlorobenzene. Raising the temperature to 200 °C led to starting material deterioration and was not productive either.

Spirocyclic products **7a–l** were tested for their ability to influence the survival of MDA-MB-231 (breast adenocarcinoma) and NCI-H460 (lung cancer) cell lines and proved completely non-cytotoxic. This validates these new compounds as suitable molecular probes for interrogating various biological targets via screening in cell-based assays.

## Conclusion

We have developed a straightforward access to novel spiro[benzofuran-2,3'-pyrrolidine]-2',5'-diones based on Rh_2_(esp)_2_-catalyzed insertion of carbenes derived from α-diazosuccinimides (DAS) into the O–H bond of phenols. The initial adducts underwent a thermally promoted Claisen rearrangement followed by a DABCO-catalyzed intramolecular 5-*exo*-*dig* Michael addition. The resulting spirocyclic compounds are formed with a clear preference to the *syn* diastereomer over *anti* which can be rationalized by conformational analysis of the Claisen rearrangement precursors to their formation.

## Supporting Information

Deposition number 2166113 (for *syn***-7a**) contains the supplementary crystallographic data for this paper. These data are provided free of charge by the joint Cambridge Crystallographic Data Centre and Fachinformationszentrum Karlsruhe Access Structures service http://www.ccdc.cam.ac.uk/structures.

File 1 General experimental information, X-ray crystallographic data, synthetic procedures, analytical data and NMR spectra for the reported compounds.
