# Supplementary material for: A novel spirocyclic scaffold accessed via tandem Claisen rearrangement/intramolecular oxa-Michael addition
Source: Beilstein J Org Chem. 2022 Dec 6;18:1649–55. doi: 10.3762/bjoc.18.177 (PMC9749547; doi:10.3762/bjoc.18.177)
Supplement: File 1 — General experimental information, X-ray crystallographic data, synthetic procedures, analytical data and NMR spectra for the reported compounds. [file Beilstein_J_Org_Chem-18-1649-s001.pdf]

BEILSTEIN JOURNAL OF ORGANIC CHEMISTRY

Supporting Information

for

A novel spirocyclic scaffold accessed via tandem Claisen rearrangement/intramolecular oxa-Michael addition

Anastasia Vepreva, Alexander Yanovich, Dmitry Dar'in, Grigory Kantin, Alexander Bunev and Mikhail Krasavin

Beilstein J. Org. Chem. **2022,** *18,* 1649–1655. doi:10.3762/bjoc.18.177

General experimental information, X-ray crystallographic data, synthetic procedures, analytical data and NMR spectra for the reported compounds

License and Terms: This is a supporting information file under the terms of the Creative Commons Attribution License (https://creativecommons.org/licenses/by/4.0). Please note that the reuse, redistribution and reproduction in particular requires that the author(s) and source are credited and that individual graphics may be subject to special legal provisions.
The license is subject to the *Beilstein Journal of Organic Chemistry* terms and conditions: (https://www.beilstein-journals.org/bjoc/terms)

Table of contents

I. Materials and methods S2

II. General procedure (GP1, GP2, GP3) for the synthesis of 5a–i, 6a and 7a–l S3

III. NMR spectra S51

IV. X-ray crystallographic data S49

V. References S51

I. Materials and methods

All commercial reagents were used without purification. Dichloromethane (DCM) was freshly distilled over P_2O_5. NMR spectra were recorded using a Bruker Avance III spectrometer in $CDCl_3$ or DMSO-d_6 (^{1}H: 400.13 MHz, ^{13}C: 100.61 MHz and 125.73 MHz). All chemical shifts are reported in parts per million (ppm). The residual solvent peak was used as internal standard: $CDCl_3$ (7.26 for ^{1}H and 77.16 ppm for ^{13}C), DMSO-d_6 (2.50 for ^{1}H and 39.52 ppm for ^{13}C). Standard abbreviations were used in the description of resonances. Coupling constants (*J*) are quoted to the nearest 0.1 Hz. Mass spectra were recorded with a HRMS-ESI-qTOF spectrometer (electrospray ionization mode, positive ion detection). Melting points were determined with a melting point apparatus Stuart SMP 10 in open capillary tubes. Single crystal X-ray data were obtained using an Agilent Technologies SuperNova Atlas and an Agilent Technologies Xcalibur Eos diffractometer at a temperature of 100 K. Analytical thin layer chromatography was carried out on UV-254 silica gel plates using appropriate eluents. Compounds were visualized with short wave length UV light. Column chromatography was performed using silica gel Merk grade 60 (0.040−0.063 mm) 230−400 mesh. Starting diazo compounds **1** were obtained according to a literature procedure. [1] Compounds **5** were obtained by modified previously reported procedure. [2]

II. General procedure (GP1) for the synthesis of 5a–n

To a solution of corresponding phenol (0.35 mmol) and catalyst (2.5 mM $Rh_2(esp)_2$ in DCM, 200 µL, 0.1 mol %) in dry DCM (1.8 mL) was slowly added a solution of the diazo compound (0.5 mmol) while cooling to 0 °C. The reaction mixture was stirred at 0 °C for 15–30 minutes (controlled by TLC). The reaction mixture was diluted with n-hexane (2 mL) and the resulting solution was subjected to column chromatography on silica gel (eluent *n*-hexane–DCM) to afford **5**.

*(E)-3-Benzylidene-4-(4-(tert-butyl)phenoxy)-1-phenylpyrrolidine-2,5-dione (**5a**)*

Prepared according to the general procedure from corresponding diazo compound **1** and 4-*tert*-butylphenol (scale – 1 mmol). Yield: 225 mg (61%). Eluent – *n*-hexane–DCM (from 20 to 60% of DCM). White solid, m.p. 175.4-176.9 °C. ^{1}H NMR (400 MHz, $CDCl_3$) δ 8.02 (d, *J* = 1.7 Hz, 1H), 7.70 (d, *J* = 7.2 Hz, 2H), 7.51 (t, *J* = 7.5 Hz, 2H), 7.48 – 7.38 (m, 4H), 7.38 – 7.32 (m, 4H), 7.15 – 7.10 (m, 2H), 5.87 (d, *J* = 1.7 Hz, 1H), 1.34 (s, 9H). ^{13}C NMR (101 MHz, $CDCl_3$) δ 171.6, 168.3, 154.5, 146.1, 141.5, 132.8, 131.5, 131.5, 131.2, 129.1, 129.1, 128.8, 126.4, 126.3, 123.6, 117.3, 72.5, 34.3, 31.5. HRMS (ESI), *m/z* calcd for $C_{27}H_{26}NO_3$ $[M+H]^+$ 412.1907 found 412.1909.

*(E)-3-Benzylidene-4-(4-fluorophenoxy)-1-phenylpyrrolidine-2,5-dione (**5b**)*

Prepared according to the general procedure from corresponding diazo compound **1** and 4-fluorophenol (scale – 1 mmol). Yield: 285 mg (85%). Eluent – *n*-hexane–DCM (from 90 to 100% of DCM). White solid, m.p. 154.0-155.8 °C. ^{1}H NMR (400 MHz, $CDCl_3$) δ 8.03 (d, *J* = 1.7 Hz, 1H), 7.66 (d, *J* = 7.3 Hz, 2H), 7.51 (m, 3H), 7.47 – 7.34 (m, 5H), 7.19 – 7.13 (m, 2H), 7.06 – 6.99 (m, 2H), 5.83 (d, *J* = 1.7 Hz, 1H). ^{13}C NMR (101 MHz, $CDCl_3$) δ 171.5, 168.1, 158.8 (d, J_{C-F} = 241.7 Hz), 152.9 (d, J_{C-F} = 2.5 Hz), 141.7, 132.7, 131.4, 131.4, 131.4, 129.2, 129.2, 128.9, 126.3, 123.2, 119.4 (d, J_{C-F} = 8.2 Hz), 116.1 (d, J_{C-F} = 23.3 Hz), 73.2. ^{19}F NMR (376 MHz, $CDCl_3$) δ -120.1. HRMS (ESI), *m/z* calcd for $C_{23}H_{17}FNO_3$ $[M+H]^+$ 374.1187 found 374.1189.

*(E)-3-Benzylidene-4-phenoxy-1-(4-(trifluoromethyl)phenyl)pyrrolidine-2,5-dione (**5c**)*

Prepared according to the general procedure from corresponding diazo compound **1** and phenol (scale – 1.5 mmol). Yield: 263 mg (46%). Eluent – *n*-hexane–DCM (from 30 to 50% of DCM). White solid, m.p. 163.4-164.6 °C. ^{1}H NMR (400 MHz, $CDCl_3$) δ 8.05 (d, *J* = 1.6 Hz, 1H), 7.78 (d, *J* = 8.3 Hz, 2H), 7.62 (dd, *J* = 22.6, 7.9 Hz, 4H), 7.47 (t, *J* = 7.4 Hz, 1H), 7.38 (dd, *J* = 16.2, 8.3 Hz, 4H), 7.22 (d, *J* = 7.9 Hz, 2H), 7.14 (t, *J* = 7.4 Hz, 1H), 5.93 (d, *J* = 1.6 Hz, 1H). ^{13}C NMR (101 MHz, $CDCl_3$) δ 171.1, 167.7, 156.9, 142.4, 134.6, 132.6, 131.5, 131.5, 130.6 (q, J_{C-F} = 33.0 Hz), 129.7, 129.2, 126.5, 126.2 (q, J_{C-F} = 3.7 Hz), 125.0, 123.7 (q, J_{C-F} = 273.7 Hz), 122.9, 117.4, 72.2. ^{19}F NMR (376 MHz, $CDCl_3$) δ -62.7. HRMS (ESI), *m/z* calcd for HRMS (ESI), *m/z* calcd for $C_{24}H_{17}F_3NO_3$ $[M+H]^+$ 424.1155 found 424.1151.

*(E)-3-(4-Fluorobenzylidene)-4-phenoxy-1-phenylpyrrolidine-2,5-dione (**5d**)*

Prepared according to the general procedure from corresponding diazo compound **1** and phenol (scale – 1.5 mmol). Yield: 407 mg (81%). Eluent – *n*-hexane–DCM (from 50 to 100% of DCM). White solid, m.p. 173.4-174.2 °C. ^{1}H NMR (400 MHz, $CDCl_3$) δ 7.98 (d, *J* = 1.6 Hz, 1H), 7.65 (dd, *J* = 8.7, 5.4 Hz, 2H), 7.52 (t, *J* = 7.5 Hz, 2H), 7.47 – 7.43 (m, 1H), 7.42 – 7.31 (m, 4H), 7.21 (d, *J* =

8.0 Hz, 2H), 7.13 (t, J = 7.5 Hz, 1H), 7.07 (t, J = 8.7 Hz, 2H), 5.89 (d, J = 1.6 Hz, 1H). ^{13}C NMR (101 MHz, $CDCl_3$) δ 171.4, 168.0, 164.3 (d, $J_{C\text{-}F}$ = 254.7 Hz), 156.9, 140.1, 133.7 (d, $J_{C\text{-}F}$ = 8.9 Hz), 131.5, 129.7, 129.2, 129.1 (d, $J_{C\text{-}F}$ = 3.4 Hz), 128.8, 126.3, 123.4, 122.9 (d, $J_{C\text{-}F}$ = 2.4 Hz), 117.5, 116.4 (d, $J_{C\text{-}F}$ = 21.9 Hz), 72.2. ^{19}F NMR (376 MHz, $CDCl_3$) δ -106.8. HRMS (ESI), *m/z* calcd for $C_{23}H_{17}FNO_3$ $[M+H]^+$ 374.1187 found 374.1185.

*(E)-3-Benzylidene-1-(4-fluorophenyl)-4-phenoxypyrrolidine-2,5-dione (**5e**)*

Prepared according to the general procedure from corresponding diazo compound **1** and phenol (scale – 1.5 mmol). Yield: 397 mg (79%). Eluent – *n*-hexane–DCM (from 80 to 100% of DCM). White solid, m.p. 162.1-162.7 °C. ^{1}H NMR (400 MHz, $CDCl_3$) δ 8.02 (d, J = 1.6 Hz, 1H), 7.64 (d, J = 7.4 Hz, 2H), 7.46 (t, J = 7.4 Hz, 1H), 7.42 – 7.32 (m, 6H), 7.25 – 7.17 (m, 4H), 7.13 (t, J = 7.3 Hz, 1H), 5.90 (d, J = 1.6 Hz, 1H). ^{13}C NMR (101 MHz, $CDCl_3$) δ 171.5, 168.1, 162.3 (d, $J_{C\text{-}F}$ = 249.0 Hz), 156.9, 141.9, 132.7, 131.4, 131.4, 129.6, 129.1, 128.2 (d, $J_{C\text{-}F}$ = 8.8 Hz), 127.4 (d, $J_{C\text{-}F}$ = 3.2 Hz), 123.3, 123.2, 117.5, 116.2 (d, $J_{C\text{-}F}$ = 23.0 Hz), 72.3. ^{19}F NMR (376 MHz, $CDCl_3$) δ -111.9. HRMS (ESI), *m/z* calcd for $C_{23}H_{17}FNO_3$ $[M+Na]^+$ 374.1187 found 374.1193.

*(E)-3-Benzylidene-4-phenoxy-1-phenylpyrrolidine-2,5-dione (**5f**)*

Prepared according to the general procedure from corresponding diazo compound **1** and phenol (scale – 1 mmol). Yield: 213 mg (67%). Eluent – *n*-hexane–DCM (from 30 to 100% of DCM). White solid, m.p. 169.2-171.0 °C. 1H NMR (400 MHz, $CDCl_3$) δ 8.03 (d, J = 1.7 Hz, 1H), 7.65 (d, J = 7.3 Hz, 2H), 7.52 (t, J = 7.5 Hz, 2H), 7.45 (d, J = 7.3 Hz, 2H), 7.42 – 7.32 (m, 6H), 7.22 (d, J = 7.9 Hz, 2H), 7.12 (t, J = 7.3 Hz, 1H), 5.91 (d, J = 1.7 Hz, 1H). ^{13}C NMR (126 MHz, $CDCl_3$) δ 171.6, 168.2, 156.9, 141.6, 132.7, 131.5, 131.4, 131.3, 129.6, 129.2, 129.1, 128.8, 126.3, 123.4, 123.3, 117.6, 72.3. HRMS (ESI), *m/z* calcd for $C_{23}H_{18}NO_3$ $[M+H]^+$ 356.1281 found 356.1282.

*(E)-1-Benzyl-3-benzylidene-4-phenoxypyrrolidine-2,5-dione (**5g**)*

Prepared according to the general procedure from corresponding diazo compound **1** and phenol (scale – 1.5 mmol). Yield: 331 mg (60%). Eluent – *n*-hexane–DCM (from 30 to 70% of DCM). White solid, m.p. 157.0-158.9 °C. ^{1}H NMR (400 MHz, $CDCl_3$) δ 7.89 (d, J = 1.7 Hz, 1H), 7.58 (d, J = 7.3 Hz, 2H), 7.42 (m, 3H), 7.33 (m, 6H), 7.26 (s, 1H), 7.09 (m, 3H), 5.76 (d, J = 1.7 Hz, 1H), 4.86 (d, J = 14.0 Hz, 1H), 4.80 (d, J = 14.0 Hz, 1H). ^{13}C NMR (101 MHz, $CDCl_3$) δ 172.3, 168.7, 156.9, 140.6, 135.3, 132.7, 131.3, 131.1, 129.5, 129.0, 128.9, 128.7, 128.1, 123.6, 123.1, 117.3, 72.2, 42.7. HRMS (ESI), *m/z* calcd for $C_{24}H_{20}NO_3$ $[M+Na]^+$ 370.1438 found 370.1437.

*(E)-3-Benzylidene-4-(3,5-dimethylphenoxy)-1-phenylpyrrolidine-2,5-dione (**5h**)*

Prepared according to the general procedure from corresponding diazo compound **1** and 3,5-dimethylphenol (scale – 1 mmol). Yield: 184 mg (53%). Eluent – *n*-hexane–DCM (from 20 to 70% of DCM). White solid, m.p. 149.1-149.7 °C. ^{1}H NMR (400 MHz, $CDCl_3$) δ 8.01 (d, J = 1.7 Hz, 1H), 7.66 (d, J = 7.3 Hz, 2H), 7.52 (t, J = 7.6 Hz, 2H), 7.48 – 7.43 (m, 2H), 7.43 – 7.37 (m, 4H), 6.83 (s, 2H), 6.76 (s, 1H), 5.88 (d, J = 1.7 Hz, 1H), 2.31 (s, 6H). ^{13}C NMR (101 MHz, $CDCl_3$) δ 171.7, 168.2, 157.1, 141.4, 139.3, 132.8, 131.6, 131.5, 131.2, 129.1, 129.1, 128.8, 126.3, 125.0, 123.6, 115.2, 72.2, 21.5. HRMS (ESI), *m/z* calcd for $C_{25}H_{22}NO_3$ $[M+H]^+$ 384.1594 found 384.1595.

*(E)-3-(4-Methylbenzylidene)-4-phenoxy-1-phenylpyrrolidine-2,5-dione (**5i**)*

Prepared according to the general procedure from corresponding diazo compound **1** and phenol (scale – 1.5 mmol). Yield: 261 mg (52%). Eluent – *n*-hexane–DCM (from 30 to 100% of DCM). White solid, m.p. 158.2-159.9 °C. ^{1}H NMR (400 MHz, $CDCl_3$) δ 7.99 (d, *J* = 1.3 Hz, 1H), 7.59 – 7.48 (m, 4H), 7.44 (d, *J* = 7.3 Hz, 1H), 7.37 (dd, *J* = 15.8, 7.3 Hz, 4H), 7.24 (d, *J* = 8.0 Hz, 2H), 7.20 (d, *J* = 8.0 Hz, 2H), 7.13 (t, *J* = 7.3 Hz, 1H), 5.89 (d, *J* = 1.3 Hz, 1H), 2.40 (s, 3H). ^{13}C NMR (101 MHz, $CDCl_3$) δ 171.6, 168.4, 156.9, 142.2, 141.6, 131.6, 131.6, 130.0, 129.9, 129.6, 129.1, 128.7, 126.3, 123.2, 122.1, 117.6, 72.4, 21.6. HRMS (ESI), *m/z* calcd for $C_{24}H_{20}NO_3$ $[M+H]^+$ 370.1438 found 370.1437.

*(E)-Methyl 4-((2,5-dioxo-4-phenoxy-1-phenylpyrrolidin-3-ylidene)methyl)benzoate (**5j**)*

Prepared according to the general procedure from corresponding diazo compound **1** and phenol (scale – 1.5 mmol). Yield: 354 mg (64%). Eluent – *n*-hexane–DCM (from 30 to 70% of DCM). White solid, m.p. 161.2-162.3 °C. ^{1}H NMR (400 MHz, $CDCl_3$) δ 8.02 (m, 3H), 7.69 (d, *J* = 8.3 Hz, 2H), 7.52 (t, *J* = 7.5 Hz, 2H), 7.46 (d, *J* = 7.2 Hz, 1H), 7.42 – 7.33 (m, 4H), 7.20 (d, *J* = 8.0 Hz, 2H), 7.13 (t, *J* = 7.3 Hz, 1H), 5.92 (d, *J* = 1.8 Hz, 1H), 3.95 (s, 3H). ^{13}C NMR (101 MHz, $CDCl_3$) δ 171.2, 167.7, 166.2, 156.9, 139.9, 136.8, 131.9, 131.3, 131.1, 130.1, 129.7, 129.2, 128.9, 126.3, 125.8, 123.4, 117.2, 72.0, 52.4. HRMS (ESI), *m/z* calcd for $C_{25}H_{20}NO_5$ $[M+H]^+$ 414.1336 found 414.1332.

*(E)-3-Benzylidene-4-phenoxy-1-(p-tolyl)pyrrolidine-2,5-dione (**5k**)*

Prepared according to the general procedure from corresponding diazo compound **1** and phenol (scale – 1.5 mmol). Yield: 322 mg (65%). Eluent – *n*-hexane–DCM (from 70 to 100% of DCM). White solid, m.p. 164.7-165.3 °C. ^{1}H NMR (400 MHz, $CDCl_3$) δ 8.01 (d, *J* = 1.5 Hz, 1H), 7.65 (d, *J* = 7.4 Hz, 2H), 7.45 (t, *J* = 7.3 Hz, 1H), 7.42 – 7.28 (m, 7H), 7.27 – 7.20 (m, 3H), 7.12 (t, *J* = 7.3 Hz, 1H), 5.90 (d, *J* = 1.5 Hz, 1H), 2.42 (s, 3H). ^{13}C NMR (101 MHz, $CDCl_3$) δ 171.6, 168.3, 157.0, 141.3, 138.9, 132.8, 131.4, 131.2, 129.8, 129.6, 129.1, 128.9, 126.1, 123.5, 123.2, 117.6, 72.4, 21.3. HRMS (ESI), *m/z* calcd for $C_{24}H_{20}NO_3$ $[M+H]^+$ 370.1438 found 370.1437.

*(E)-3-(2-Methoxybenzylidene)-4-phenoxy-1-phenylpyrrolidine-2,5-dione (**5l**)*

Prepared according to the general procedure from corresponding diazo compound **1** and phenol (scale – 1.5 mmol). Yield: 295 mg (57%). Eluent – *n*-hexane–DCM (from 70 to 100% of DCM). White solid, m.p. 141.4-142.8 °C. ^{1}H NMR (400 MHz, $CDCl_3$) δ 8.31 (d, J = 1.9 Hz, 1H), 7.61 – 7.47 (m, 3H), 7.48 – 7.38 (m, 3H), 7.37 – 7.28 (m, 3H), 7.12 – 7.02 (m, 3H), 6.97 – 6.82 (m, 2H), 5.87 (d, J = 1.9 Hz, 1H), 3.67 (s, 3H). ^{13}C NMR (101 MHz, $CDCl_3$) δ 172.2, 168.1, 158.2, 157.9, 136.7, 132.6, 131.7, 131.5, 129.4, 129.1, 129.1, 128.6, 126.5, 126.4, 122.8, 120.7, 117.2, 111.0, 73.7, 55.2. HRMS (ESI), *m/z* calcd for $C_{24}H_{20}NO_4$ $[M+H]^+$ 386.1387 found 386.1386.

*(E)-3-Benzylidene-4-(4-methoxyphenoxy)-1-phenylpyrrolidine-2,5-dione (**5m**)*

Prepared according to the general procedure from corresponding diazo compound **1** and 4-methoxyphenol (scale – 1 mmol). Yield: 259 mg (75%). Eluent – *n*-hexane–DCM (from 50 to 100% of DCM). White solid, m.p. 72.8-74.9 °C. ^{1}H NMR (400 MHz, $CDCl_3$) δ 8.01 (d, *J* = 1.7 Hz,

1H), 7.74 (d, *J* = 7.0 Hz, 2H), 7.54 – 7.41 (m, 6H), 7.38 – 7.32 (m, 2H), 7.16 – 7.08 (m, 2H), 6.88 – 6.81 (m, 2H), 5.78 (d, *J* = 1.7 Hz, 1H), 3.80 (s, 3H). ^{13}C NMR (101 MHz, $CDCl_3$) δ 171.7, 168.2, 155.8, 150.4, 141.6, 132.9, 131.52, 131.51, 131.3, 129.2, 129.2, 128.8, 126.3, 123.5, 119.7, 114.6, 73.4, 55.6. HRMS (ESI), *m/z* calcd for C24H19NO4 $[M+H]^+$ 386.1387 found 386.1391.

*(E)-3-Benzylidene-4-(2-methoxyphenoxy)-1-phenylpyrrolidine-2,5-dione (**5n**)*

Prepared according to the general procedure from corresponding diazo compound **1** and 2-methoxyphenol (scale – 1.5 mmol). Yield: 301 mg (58%). Eluent – *n*-hexane–ethylacetate (from 15 to 30% of ethylacetate). White solid, m.p. 160.4-161.7 °C. ^{1}H NMR (400 MHz, $CDCl_3$) δ 8.02 (d, *J* = 1.7 Hz, 1H), 7.78 (d, *J* = 7.2 Hz, 2H), 7.51 (m, 2H), 7.46 – 7.36 (m, 7H), 7.14 – 7.07 (m, 1H), 6.98 – 6.91 (m, 2H), 5.98 (d, *J* = 1.7 Hz, 1H), 3.75 (s, 3H). ^{13}C NMR (101 MHz, $CDCl_3$) δ 171.2, 168.4, 150.6, 145.8, 141.8, 133.0, 131.6, 131.5, 131.0, 129.1, 128.8, 128.6, 126.3, 124.3, 123.6, 120.9, 119.3, 112.1, 72.9, 55.6. HRMS (ESI), *m/z* calcd for $C_{24}H_{20}NO_4$ $[M+H]^+$ 386.1387 found 386.1390.

General procedure (GP2) for the synthesis of 6a

To a solution of corresponding phenol (0.35 mmol) and catalyst (2.5 mM $Rh_2(esp)_2$ in DCM, 200 μL, 0.1 mol %) in dry DCM (1.8 mL) was slowly added a solution of the diazo compound (0.5 mmol) while cooling to 0 °C. The reaction mixture was stirred at 0 °C for 15-30 minutes (controlled by TLC). Then DCM was evaporated and reaction mixture was dissolved in toluene (2 mL) and stirred at 140 °C for 24 h. The reaction mixture was diluted with *n*-hexane (2 mL) and the resulting solution was subjected to column chromatography on silica gel (eluent *n*-hexane–acetone) to afford **6**.

*3-((5-(tert-Butyl)-2-hydroxyphenyl)(phenyl)methyl)-1-phenyl-1H-pyrrole-2,5-dione (**6a**)*

Prepared according to the general procedure from corresponding diazo compound **1** and 4-*tert*-butylphenol (scale – 1 mmol). Yield: 135 mg (47%) Amorphous solid. ^{1}H NMR (400 MHz, $CDCl_3$) δ 7.49 – 7.42 (m, 2H), 7.41 – 7.29 (m, 7H), 7.33 (s, 1H), 7.19 (dd, *J* = 8.3, 2.4 Hz, 1H), 7.05 (d, *J* = 2.3 Hz, 1H), 6.74 (d, *J* = 8.4 Hz, 1H), 6.34 (d, *J* = 1.6 Hz, 1H), 5.72 (s, 1H), 5.08 (s, 1H), 1.25 (s, 9H). ^{13}C NMR (101 MHz, $CDCl_3$) δ 169.7, 169.4, 151.7, 150.7, 143.8, 138.7, 131.5, 129.0, 128.8, 128.7, 128.6, 127.6, 127.3, 126.5, 125.8, 125.6, 125.4, 115.7, 42.2, 34.1, 31.4. HRMS (ESI), *m/z* calcd for $C_{27}H_{26}NO_3$ $[M+H]^+$ 412.1907 found 412.1911.

General procedure (GP3) for the synthesis of 7a–l

Compound **5** (0.5 mmol) was dissolved in toluene (2 mL) and stirred at 140 °C (150 °C for **5g**) for 8–24 h (controlled by TLC). Upon cooling to room temperature, to the solution was added DABCO (0.15 mmol, 30 mol %), and the reaction mixture was stirred for 30–45 min (controlled by TLC). Cyclized product as a mixture of diastereomers *syn*-**7** and *anti*-**7** was isolated by flash column chromatography on silica gel (*n*-hexane–acetone, from 0 to 50% of acetone). Pure major diastereomer was obtained by crystallization from *n*-hexane–acetone. In some cases pure minor diasteromer was isolated by column chromatography on silica gel (eluent *n*-hexane–MTBE).

*(2R/S,3R/S)-5-(Tert-butyl)-1',3-diphenyl-3H-spiro[benzofuran-2,3'-pyrrolidine]-2',5'-dione (syn-**7a**) and (2S/R,3R/S)-5-(tert-butyl)-1',3-diphenyl-3H-spiro[benzofuran-2,3'-pyrrolidine]-2',5'-dione*

(anti-7a): prepared according to the general procedure from compound **5a** (0.46 mmol). After column chromatography (eluent – *n*-hexane–MTBE, from 10% to 50% of MTBE) two fractions were obtained: pure major diastereomer *syn-**7a*** – 140 mg (74%) and pure minor diastereomer *anti-**7a*** – 15 mg (8%).

Compound *syn-**7a***. Yield: 140 mg (74%). White solid, m.p. 229.6-230.8 °C. ^{1}H NMR (400 MHz, $CDCl_3$) δ 7.53 (t, *J* = 7.5 Hz, 2H), 7.48 – 7.37 (m, 6H), 7.34 (dd, *J* = 8.5, 1.2 Hz, 1H), 7.22 (d, *J* = 6.8 Hz, 2H), 7.18 (s, 1H), 6.92 (d, *J* = 8.5 Hz, 1H), 5.29 (s, 1H), 2.87 (d, *J* = 18.5 Hz, 1H), 2.72 (d, *J* = 18.5 Hz, 1H), 1.32 (s, 9H). ^{13}C NMR (126 MHz, $CDCl_3$) δ 175.2, 172.3, 156.1, 145.5, 137.3, 131.4, 129.4, 129.2, 128.9, 128.9, 128.5, 127.6, 126.3, 126.2, 122.5, 109.2, 89.6, 54.2, 38.6, 34.5, 31.7. HRMS (ESI), *m/z* calcd for $C_{27}H_{26}NO_3$ $[M+H]^+$ 412.1907 found 412.1912.

Compound *anti-**7a***. Yield: 15 mg (8%) Amorphous solid. ^{1}H NMR (400 MHz, $CDCl_3$) δ 7.43 – 7.28 (m, 8H), 7.27 (m, 1H), 7.12 (m, 1H), 6.93 (d, *J* = 8.5 Hz, 1H), 6.56 – 6.52 (m, 2H), 5.08 (s, 1H), 3.63 (d, *J* = 18.0 Hz, 1H), 3.37 (d, *J* = 18.0 Hz, 1H), 1.31 (s, 9H). ^{13}C NMR (101 MHz, $CDCl_3$) δ 172.9, 171.4, 157.8, 145.2, 135.3, 130.8, 129.3, 129.1, 128.9, 128.7, 128.6, 126.9, 126.2, 126.1, 121.9, 108.9, 90.8, 60.2, 42.8, 34.5, 31.7. HRMS (ESI), *m/z* calcd for $C_{27}H_{26}NO_3$ $[M+H]^+$ 412.1907 found 412.1915.

*(2R/S,3R/S)-5-Fluoro-1',3-diphenyl-3H-spiro[benzofuran-2,3'-pyrrolidine]-2',5'-dione(syn-**7b**)*

Prepared according to the general procedure from compound **5b** (0.91 mmol). Total yield: 216 mg (64%), dr = 77:23. Individual major diastereomer *syn-**7b*** was obtained by recrystallization from *n*-hexane/acetone. White solid, m.p. 175.8 – 176.6 °C. ^{1}H NMR (400 MHz, $CDCl_3$) δ 7.52 (m, 2H), 7.44 (m, 4H), 7.36 (d, J = 7.5 Hz, 2H), 7.16 (d, J = 6.4 Hz, 2H), 6.98 (td, J = 8.7, 2.4 Hz, 1H), 6.90 (m, 1H), 6.84 (dd, J = 7.7, 1.9 Hz, 1H), 5.20 (s, 1H), 2.88 (d, J = 18.6 Hz, 1H), 2.71 (d, J = 18.6 Hz, 1H). ^{13}C NMR (126 MHz, $CDCl_3$) δ 174.8, 172.0, 158.5 (d, J_{C-F} = 239.6 Hz), 154.2 (d, J_{C-F} = 1.4 Hz), 136.8, 131.3, 130.0 (d, J_{C-F} = 8.7 Hz), 129.6, 129.3, 128.9, 128.8, 128.8, 126.2, 115.7 (d, J_{C-F} = 24.5 Hz), 112.7 (d, J_{C-F} = 25.4 Hz), 110.4 (d, J_{C-F} = 8.5 Hz), 90.1, 53.8 (d, J_{C-F} = 1.7 Hz), 38.4. ^{19}F NMR (470 MHz, $CDCl_3$) δ -121.6. HRMS (ESI), *m/z* calcd for $C_{24}H_{21}FNO_4$ $[M+MeOH+H]^+$ 406.1449 found 406.1456.

*(2R/S,3R/S)-3-Phenyl-1'-(4-(trifluoromethyl)phenyl)-3H-spiro[benzofuran-2,3'-pyrrolidine]-2',5'-dione (syn-**7c**) and (2S,3R)-3-phenyl-1'-(4-(trifluoromethyl)phenyl)-3H-spiro[benzofuran-2,3'-pyrrolidine]-2',5'-dione (anti-**7c**):* prepared according to the general procedure from compound **5c** (0.35 mmol). After column chromatography (eluent – *n*-hexane–MTBE, from 10% to 50% of MTBE) two fractions were obtained: pure major diastereomer *syn-**7c*** – 66 mg (45%) and pure minor diastereomer *anti-**7c*** – 23 mg (16%).

Compound *syn-**7c***. Yield: 66 mg (45%). White solid, m.p. 150.2-150.5 °C. ^{1}H NMR (400 MHz, $CDCl_3$) δ 7.79 (d, J = 8.4 Hz, 2H), 7.57 (d, J = 8.4 Hz, 2H), 7.45 – 7.39 (m, 3H), 7.31 (m, 1H), 7.20 – 7.11 (m, 3H), 7.06 – 6.95 (m, 2H), 5.24 (s, 1H), 2.90 (d, J = 18.7 Hz, 1H), 2.74 (d, J = 18.7 Hz, 1H). ^{13}C NMR (101 MHz, $CDCl_3$) δ 174.6, 171.7, 158.1, 137.3, 134.5, 130.8 (q, J_{C-F} = 32.9 Hz), 129.5, 129.4, 128.9, 128.6, 128.3, 126.5, 126.3 (q, J_{C-F} = 3.7 Hz), 125.8 (q, J_{C-F} = 172.7 Hz) 125.6, 122.5, 110.1, 89.4, 53.8, 38.5. ^{19}F NMR (376 MHz, $CDCl_3$) δ -62.7. HRMS (ESI), *m/z* calcd for $C_{25}H_{21}F_3NO_4$ $[M+MeOH+H]^+$ 456.1417 found 456.1423.

Compound *anti-**7c***. Yield: 23 mg (16%) Amorphous solid. ^{1}H NMR (400 MHz, $CDCl_3$) δ 7.55 (d, J = 8.4 Hz, 2H), 7.43 – 7.29 (m, 6H), 7.12 (d, J = 7.3 Hz, 1H), 7.07 – 7.01 (m, 2H), 6.70 (d, J = 8.4 Hz, 2H), 5.11 (s, 1H), 3.68 (d, J = 18.0 Hz, 1H), 3.41 (d, J = 18.0 Hz, 1H). ^{13}C NMR (101 MHz,

$CDCl_3$) δ 172.5, 170.7, 159.9, 134.9, 133.8, 130.7 (q, J_{C-F} = 33.1 Hz), 129.6, 129.3, 129.2, 128.8, 127.2, 126.4, 126.06 (q, J_{C-F} = 3.7 Hz), 125.0, 125.6 (q, J_{C-F} = 132.3 Hz), 122.2, 109.9, 90.7, 59.9, 42.6. ^{19}F NMR (376 MHz, $CDCl_3$) δ -62.8. HRMS (ESI), *m/z* calcd for $C_{25}H_{21}F_3NO_4$ $[M+MeOH+H]^+$ 456.1417 found 456.1392.

*(2R/S,3R/S)-1'-(4-Fluorophenyl)-3-phenyl-3H-spiro[benzofuran-2,3'-pyrrolidine]-2',5'-dione (syn-**7d**)*

Prepared according to the general procedure from compound **5d** (0.56 mmol). Total yield: 112 mg (54%), dr = 75:25. Individual major diastereomer *syn-**7d*** was obtained by recrystallization from *n*-hexane/acetone. White solid, m.p. 213.5-214.3 °C. ^1H NMR (400 MHz, $CDCl_3$) δ 7.45 – 7.34 (m, 5H), 7.31 (m, 1H), 7.24 – 7.11 (m, 5H), 7.07 – 6.94 (m, 2H), 5.23 (s, 1H), 2.87 (d, *J* = 18.6 Hz, 1H), 2.72 (d, *J* = 18.6 Hz, 1H). ^{13}C NMR (101 MHz, $CDCl_3$) δ 174.9, 172.1, 162.3 (d, J_{C-F} = 249.1 Hz), 158.2, 137.3, 129.5, 129.3, 128.9, 128.6, 128.6, 128.1 (d, J_{C-F} = 8.8 Hz), 127.3 (d, J_{C-F} = 3.2 Hz), 125.6, 122.4, 116.3 (d, J_{C-F} = 23.0 Hz), 110.1, 89.4, 53.7, 38.4. ^{19}F NMR (376 MHz, $CDCl_3$) δ -111.6. HRMS (ESI), *m/z* calcd for $C_{24}H_{21}FNO_4$ $[M+MeOH+H]^+$ 406.1449 found 406.1453.

*(2R/S,3R/S)-3-(4-Fluorophenyl)-1'-phenyl-3H-spiro[benzofuran-2,3'-pyrrolidine]-2',5'-dione (syn-**7e**)*

Prepared according to the general procedure from compound **5e** (0.28 mmol). Total yield: 62 mg (60%), dr = 82:18. Individual major diastereomer *syn*-**7e** was obtained by recrystallization from *n*-hexane/acetone. Amorphous solid. ^1H NMR (400 MHz, $CDCl_3$) δ 7.52 (t, *J* = 7.5 Hz, 2H), 7.44 (t, *J* = 7.4 Hz, 1H), 7.40 – 7.36 (m, 2H), 7.31 (m, 1H), 7.19 – 7.09 (m, 5H), 7.05 – 6.96 (m, 2H), 5.22 (s, 1H), 2.89 (d, *J* = 18.5 Hz, 1H), 2.70 (d, *J* = 18.5 Hz, 1H). ^{13}C NMR (101 MHz, $CDCl_3$) δ 174.9, 172.0, 162.6 (d, J_{C-F} = 248.4 Hz), 158.2, 133.3 (d, J_{C-F} = 3.3 Hz), 131.3, 130.6 (d, J_{C-F} = 8.2 Hz), 129.5, 129.2, 128.9, 128.4, 126.2, 125.5, 122.4, 116.4 (d, J_{C-F} = 21.6 Hz), 110.2, 89.3, 52.9, 38.4. ^{19}F NMR (376 MHz, $CDCl_3$) δ -112.8. HRMS (ESI), *m/z* calcd for $C_{24}H_{21}FNO_4$ $[M+MeOH+H]^+$ 406.1449 found 406.1453.

*(2R/S,3R/S)-1',3-Diphenyl-3H-spiro[benzofuran-2,3'-pyrrolidine]-2',5'-dione (syn-**7f**):*

Prepared according to the general procedure from compound **5f** (0.59 mmol). Total yield: 85 mg (41%), dr = 81:19. Individual major diastereomer *syn-**7f*** was obtained by recrystallization from *n*-hexane/acetone. White solid, m.p. 196.4-196.8 °C. ^1H NMR (400 MHz, $CDCl_3$) δ 7.52 (t, *J* = 7.5 Hz, 2H), 7.48 – 7.35 (m, 6H), 7.30 (m, 1H), 7.20 – 7.16 (m, 2H), 7.14 (d, *J* = 7.4 Hz, 1H), 7.05 – 6.95 (m, 2H), 5.25 (s, 1H), 2.88 (d, *J* = 18.6 Hz, 1H), 2.72 (d, *J* = 18.6 Hz, 1H). ^{13}C NMR (101 MHz, $CDCl_3$) δ 175.0, 172.2, 158.3, 137.3, 131.4, 129.4, 129.3, 129.2, 128.9, 128.9, 128.5, 128.3, 126.3, 125.6, 122.3, 110.1, 89.4, 53.8, 38.5. HRMS (ESI), *m/z* calcd for $C_{23}H_{18}NO_3$ $[M+H]^+$ 356.1281 found 356.1289.

*(2R/S,3R/S)-1'-Benzyl-3-phenyl-3H-spiro[benzofuran-2,3'-pyrrolidine]-2',5'-dione (syn-**7g**):*

Prepared according to the general procedure from compound **5g** (0.56 mmol). Total yield: 106 mg (52%), dr = 73:27. Individual major diastereomer *syn-**7g*** was obtained by HPLC (eluent – *n*-hexane/MTBE). Amorphous solid. ^1H NMR (400 MHz, $CDCl_3$) δ 7.38 (m, 5H), 7.32 – 7.29 (m, 3H), 7.25 (d, *J* = 7.8 Hz, 1H), 7.09 (d, *J* = 7.4 Hz, 1H), 7.02 – 6.91 (m, 4H), 5.07 (s, 1H), 4.77 (d, *J* = 14.0 Hz, 1H), 4.71 (d, *J* = 14.0 Hz, 1H), 2.71 (d, *J* = 18.6 Hz, 1H), 2.54 (d, *J* = 18.6 Hz, 1H). ^{13}C NMR (101 MHz, $CDCl_3$) δ 175.9, 172.8, 158.4, 137.1, 135.2, 129.2, 129.2, 128.8, 128.8, 128.7, 128.3, 128.2, 128.1, 125.5, 122.1, 110.0, 89.2, 53.8, 42.7, 38.3. HRMS (ESI), *m/z* calcd for $C_{24}H_{20}NO_3$ $[M+H]^+$ 370.1438 found 370.1432.

*(2R/S,3R/S)-4,6-Dimethyl-1',3-diphenyl-3H-spiro[benzofuran-2,3'-pyrrolidine]-2',5'-dione (syn-**7h**)*

Prepared according to the general procedure from compound **5h** (0.43 mmol). Total yield: 85 mg (52%), dr = 92:8. Individual major diastereomer *syn-**7h*** was obtained by recrystallization from *n*-hexane/acetone. White solid, m.p. 224.1-224.6 °C. ^{1}H NMR (400 MHz, $CDCl_3$) δ 7.51 (t, *J* = 7.5 Hz, 2H), 7.46 – 7.31 (m, 6H), 7.09 (s, 2H), 6.63 (d, *J* = 12.6 Hz, 2H), 4.92 (s, 1H), 2.88 (d, *J* = 18.7 Hz, 1H), 2.65 (d, *J* = 18.7 Hz, 1H), 2.35 (s, 3H), 1.92 (s, 3H). ^{13}C NMR (101 MHz, $CDCl_3$) δ 175.1, 172.4, 158.4, 139.5, 138.1, 135.1, 131.4, 129.3, 129.1, 128.8, 128.6, 128.2, 126.3, 124.9, 124.6, 107.9, 89.1, 52.7, 38.6, 21.5, 18.5. HRMS (ESI), *m/z* calcd for $C_{25}H_{22}NO_3$ $[M+H]^+$ 384.1594 found 384.1598.

*(2R/S,3R/S)-3-Phenyl-1'-(p-tolyl)-3H-spiro[benzofuran-2,3'-pyrrolidine]-2',5'-dione (syn-**7i**)*

Prepared according to the general procedure from compound **5i** (0.51 mmol). Total yield: 86 mg (46%), dr = 79:21. Individual major diastereomer *syn-**7i*** was obtained by recrystallization from *n*-hexane/acetone. White solid, m.p. 194.1-194.8 °C. ^{1}H NMR (400 MHz, $CDCl_3$) δ 7.46 – 7.36 (m, 3H), 7.32 (m, 3H), 7.25 (m, 2H), 7.20 – 7.10 (m, 3H), 7.06 – 6.93 (m, 2H), 5.25 (s, 1H), 2.86 (d, *J* = 18.6 Hz, 1H), 2.71 (d, *J* = 18.6 Hz, 1H), 2.42 (s, 3H). ^{13}C NMR (101 MHz, $CDCl_3$) δ 175.1, 172.3, 158.3, 139.0, 137.3, 129.8, 129.4, 129.2, 128.9, 128.7, 128.5, 128.3, 126.0, 125.6, 122.2, 110.0, 89.4, 53.8, 38.4, 21.2. HRMS (ESI), *m/z* calcd for $C_{24}H_{20}NO_3$ $[M+H]^+$ 370.1438 found 370.1436.

*Methyl 4-((2R/S,3R/S)-2',5'-dioxo-1'-phenyl-3H-spiro[benzofuran-2,3'-pyrrolidin]-3-yl)benzoate (syn-**7j**)*

Prepared according to the general procedure from compound **5j** (0.68 mmol). Total yield: 83 mg (30%), dr = 67:33. Individual major diastereomer *syn-**7j*** was obtained by recrystallization from *n*-hexane/acetone. White solid, m.p. 191.1-191.8 °C. ^{1}H NMR (400 MHz, $CDCl_3$) δ 8.09 (d, *J* = 8.3 Hz, 2H), 7.52 (m, 2H), 7.48 – 7.42 (m, 1H), 7.40 – 7.34 (m, 2H), 7.32 (d, *J* = 7.5 Hz, 1H), 7.26 (d, *J* = 8.3 Hz, 2H), 7.10 (d, *J* = 7.5 Hz, 1H), 7.02 (m, 2H), 5.28 (s, 1H), 3.96 (s, 3H), 2.90 (d, *J* = 18.6 Hz, 1H), 2.64 (d, *J* = 18.6 Hz, 1H). ^{13}C NMR (101 MHz, $CDCl_3$) δ 174.6, 171.8, 166.3, 158.2, 142.5, 131.2, 130.6, 130.4, 129.6, 129.2, 128.9, 128.9, 127.9, 126.2, 125.5, 122.5, 110.2, 89.1, 53.4, 52.3, 38.4. HRMS (ESI), *m/z* calcd for $C_{25}H_{20}NO_5$ $[M+H]^+$ 414.1336 found 414.1333.

*(2R/S,3R/S)-1'-Phenyl-3-(p-tolyl)-3H-spiro[benzofuran-2,3'-pyrrolidine]-2',5'-dione (syn-**7k**)*

Prepared according to the general procedure from compound **5k** (0.65 mmol). Total yield: 101 mg (43%), dr = 82:18. Individual major diastereomer *syn-**7k*** was obtained by recrystallization from *n*-hexane/acetone. White solid, m.p. 225.9-226.3 °C. ^{1}H NMR (400 MHz, $CDCl_3$) δ 7.52 (t, *J* = 7.7 Hz, 2H), 7.44 (t, *J* = 7.3 Hz, 1H), 7.37 (d, *J* = 7.7 Hz, 2H), 7.29 (s, 1H), 7.22 (d, *J* = 7.8 Hz, 2H), 7.12 (d, *J* = 7.4 Hz, 1H), 7.10 – 6.94 (m, 4H), 5.22 (s, 1H), 2.86 (d, *J* = 18.6 Hz, 1H), 2.75 (d, *J* = 18.6 Hz, 1H), 2.39 (s, 3H). ^{13}C NMR (101 MHz, $CDCl_3$) δ 175.2, 172.3, 158.3, 138.4, 134.1, 131.4, 130.1, 129.2, 129.2, 128.9, 128.8, 128.4, 126.3, 125.6, 122.3, 110.0, 89.4, 53.7, 38.5, 21.1. HRMS (ESI), *m/z* calcd for $C_{24}H_{20}NO_3$ $[M+H]^+$ 370.1438 found 370.1435.

*(2R/S,3R/S)-3-(2-Methoxyphenyl)-1'-phenyl-3H-spiro[benzofuran-2,3'-pyrrolidine]-2',5'-dione (syn-**7l**)*

Prepared according to the general procedure from compound **5l** (0.45 mmol). Total yield: 82 mg (47%), dr = 80:20. Individual major diastereomer *syn-**7l*** was obtained by recrystallization from *n*-hexane/acetone. Amorphous solid. ^{1}H NMR (400 MHz, $CDCl_3$) δ 7.57 – 7.50 (m, 2H), 7.48 – 7.42

(m, 3H), 7.35 (m, 2H), 7.20 (d, *J* = 7.5 Hz, 1H), 7.05 (dd, *J* = 7.5, 1.0 Hz, 2H), 7.03 – 6.94 (m, 3H), 5.55 (s, 1H), 3.78 (s, 3H), 2.84 (d, *J* = 18.6 Hz, 1H), 2.60 (d, *J* = 18.6 Hz, 1H). ^{13}C NMR (101 MHz, $CDCl_3$) δ 175.7, 172.8, 158.9, 157.0, 131.7, 129.5, 129.1, 129.1, 128.9, 128.6, 126.5, 126.2, 126.1, 126.0, 121.7, 121.3, 110.4, 109.8, 88.5, 55.5, 47.9, 38.6. HRMS (ESI), *m/z* calcd for $C_{24}H_{20}NO_4$ $[M+H]^+$ 386.1387 found 386.1393.

III. NMR spectra

Copies of ^{1}H (400.13 MHz, $CDCl_3$) and ^{13}C (100.61 MHz, $CDCl_3$) spectra of **5a**

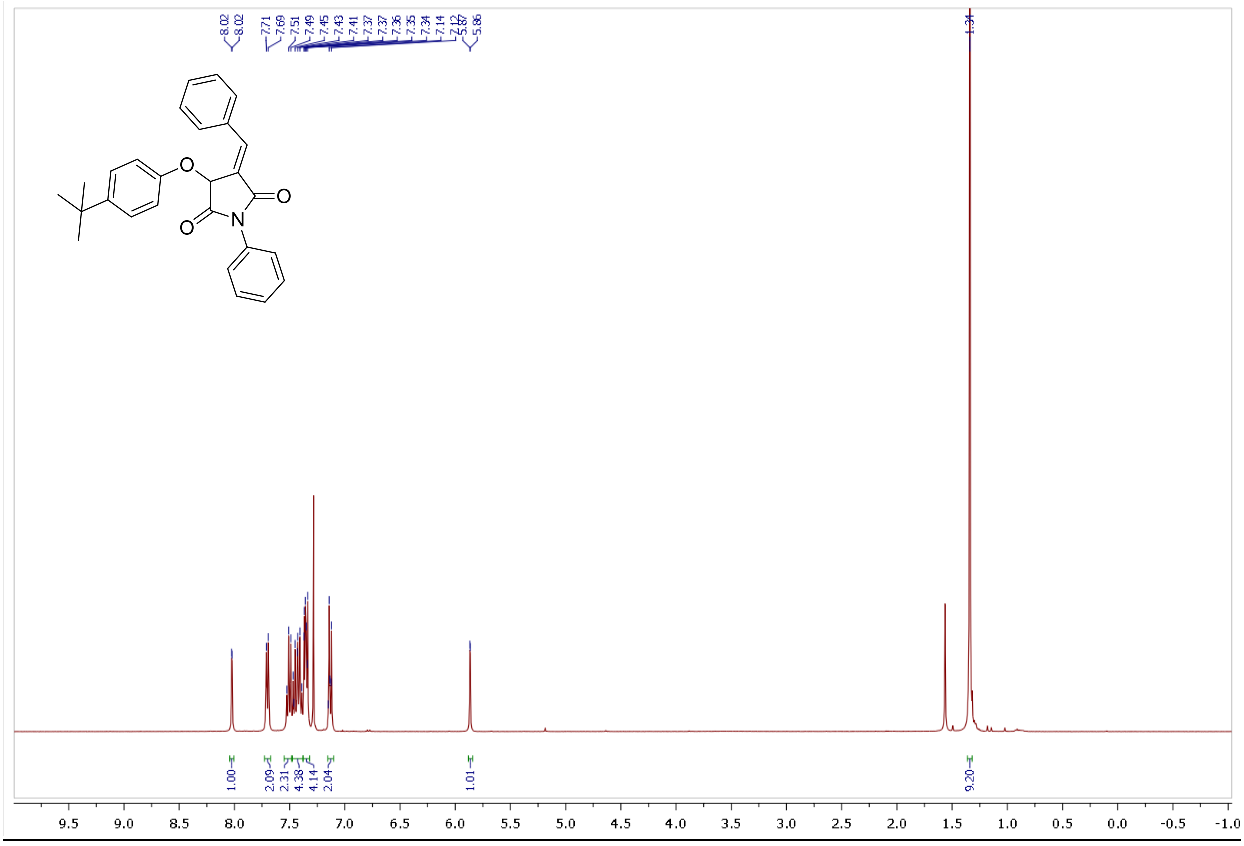

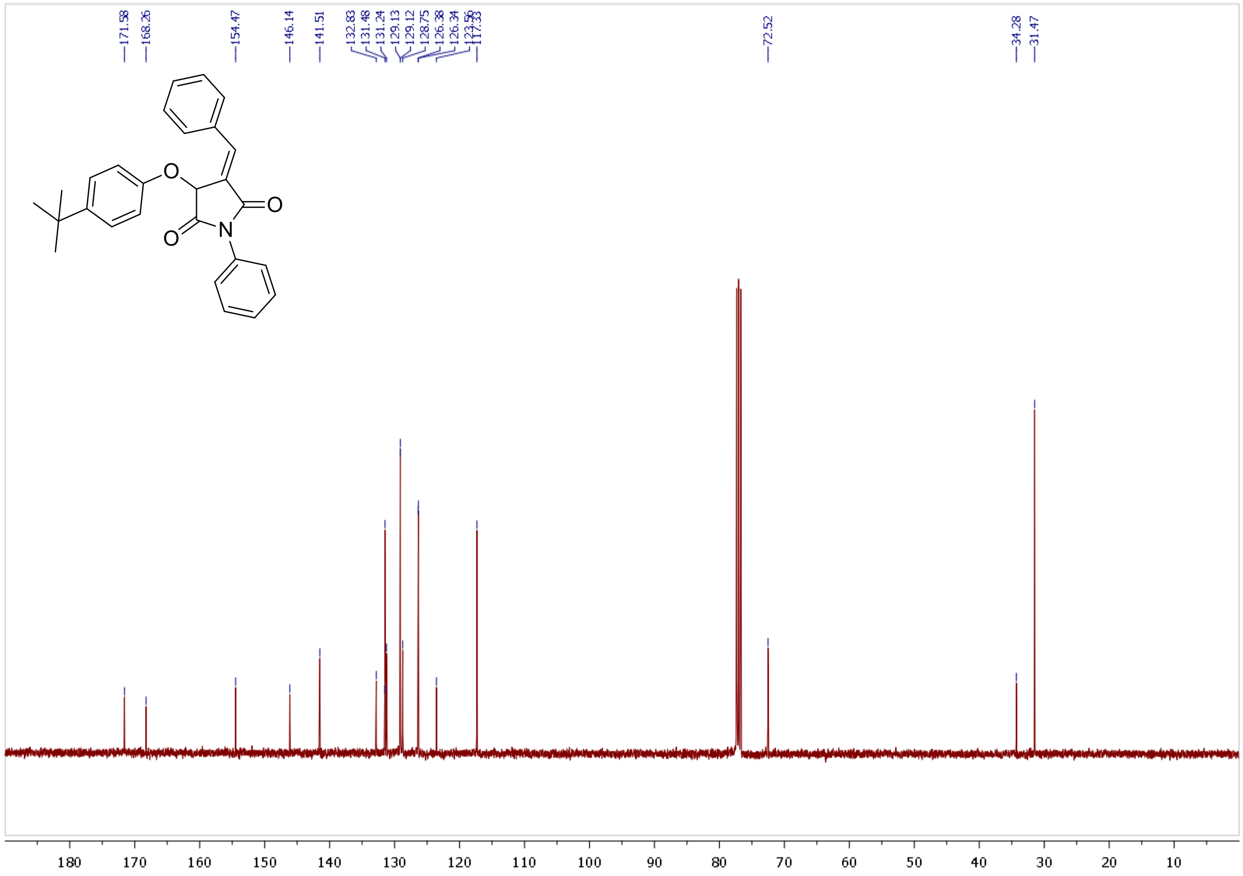

Copies of ^{1}H (400.13 MHz, $CDCl_3$) and ^{13}C (100.61 MHz, $CDCl_3$) spectra of **5b**

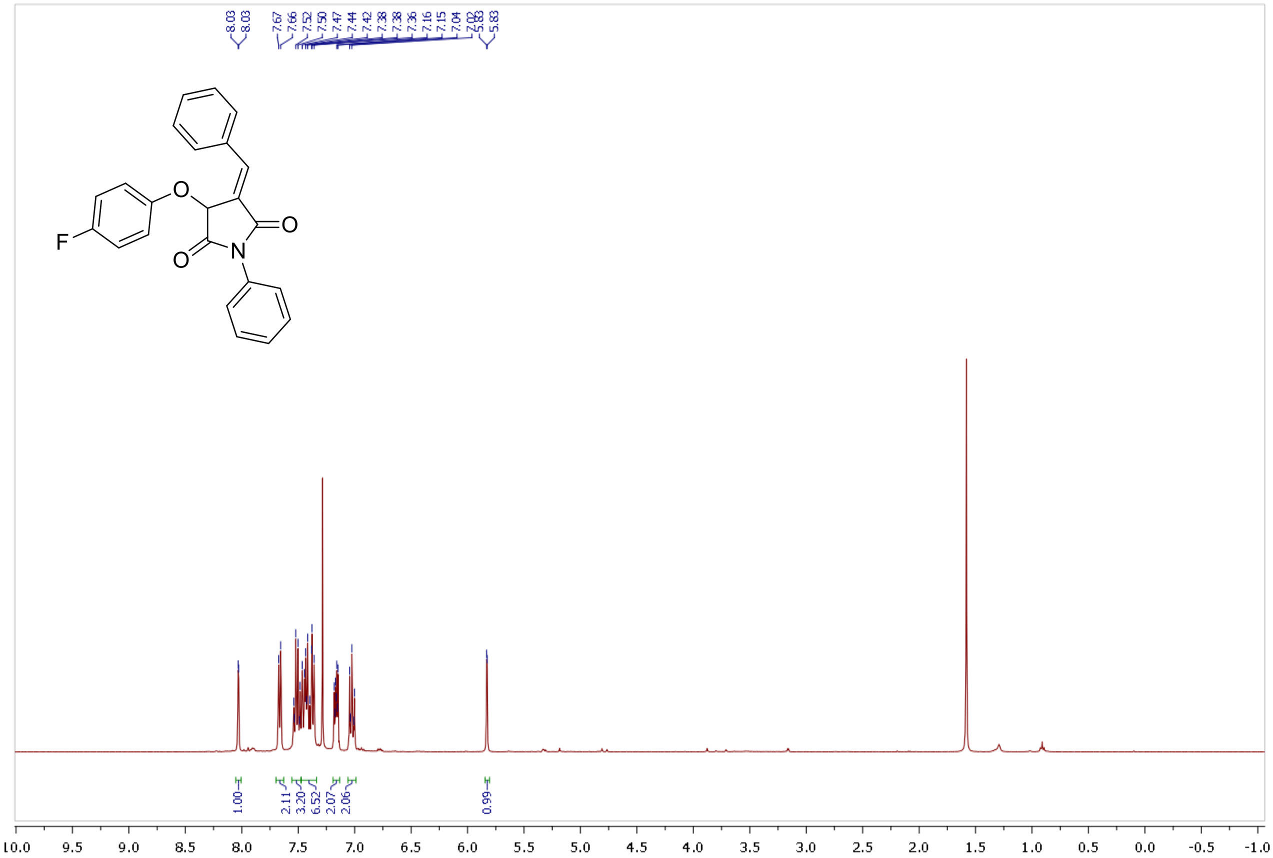

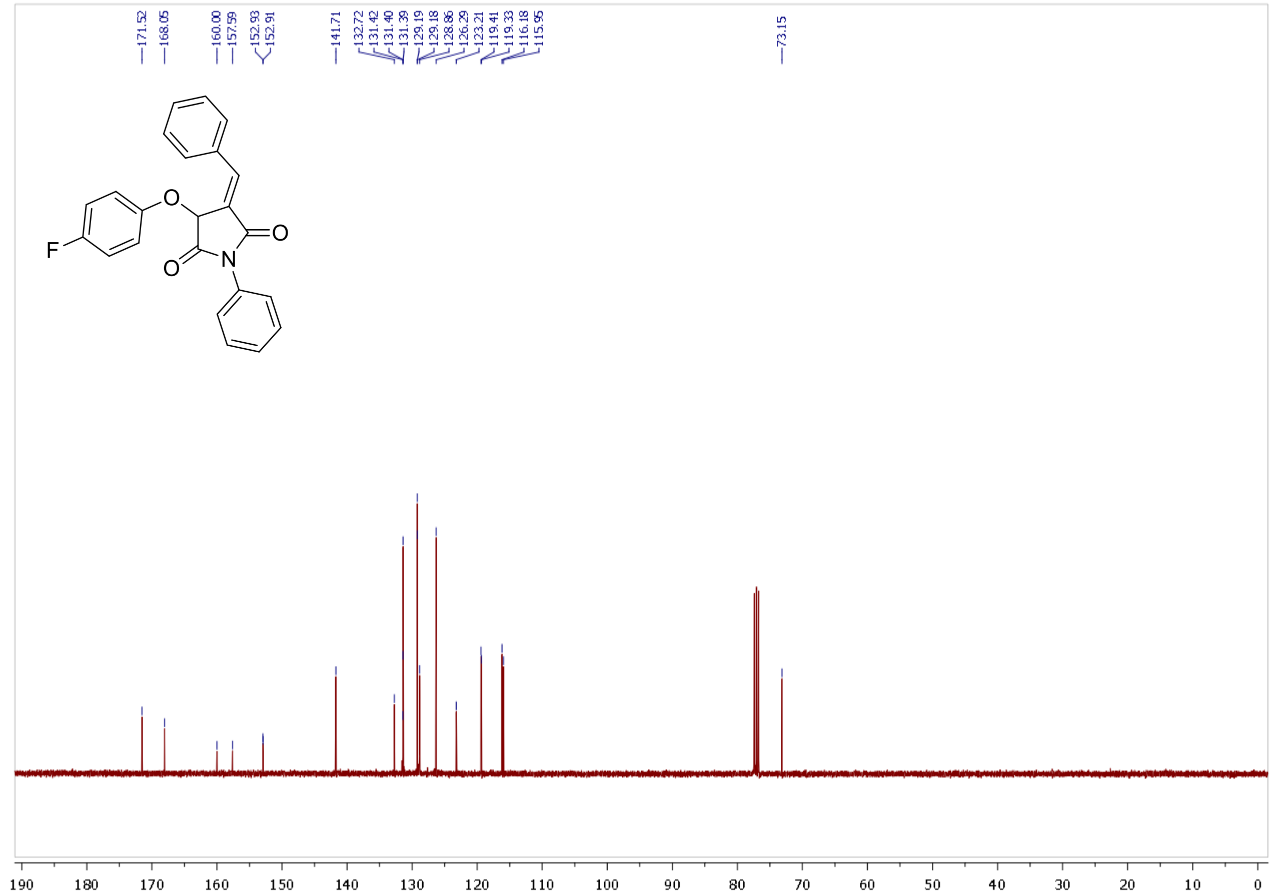

Copy of ^{19}F (376.50 MHz, $CDCl_3$) spectrum of **5b**

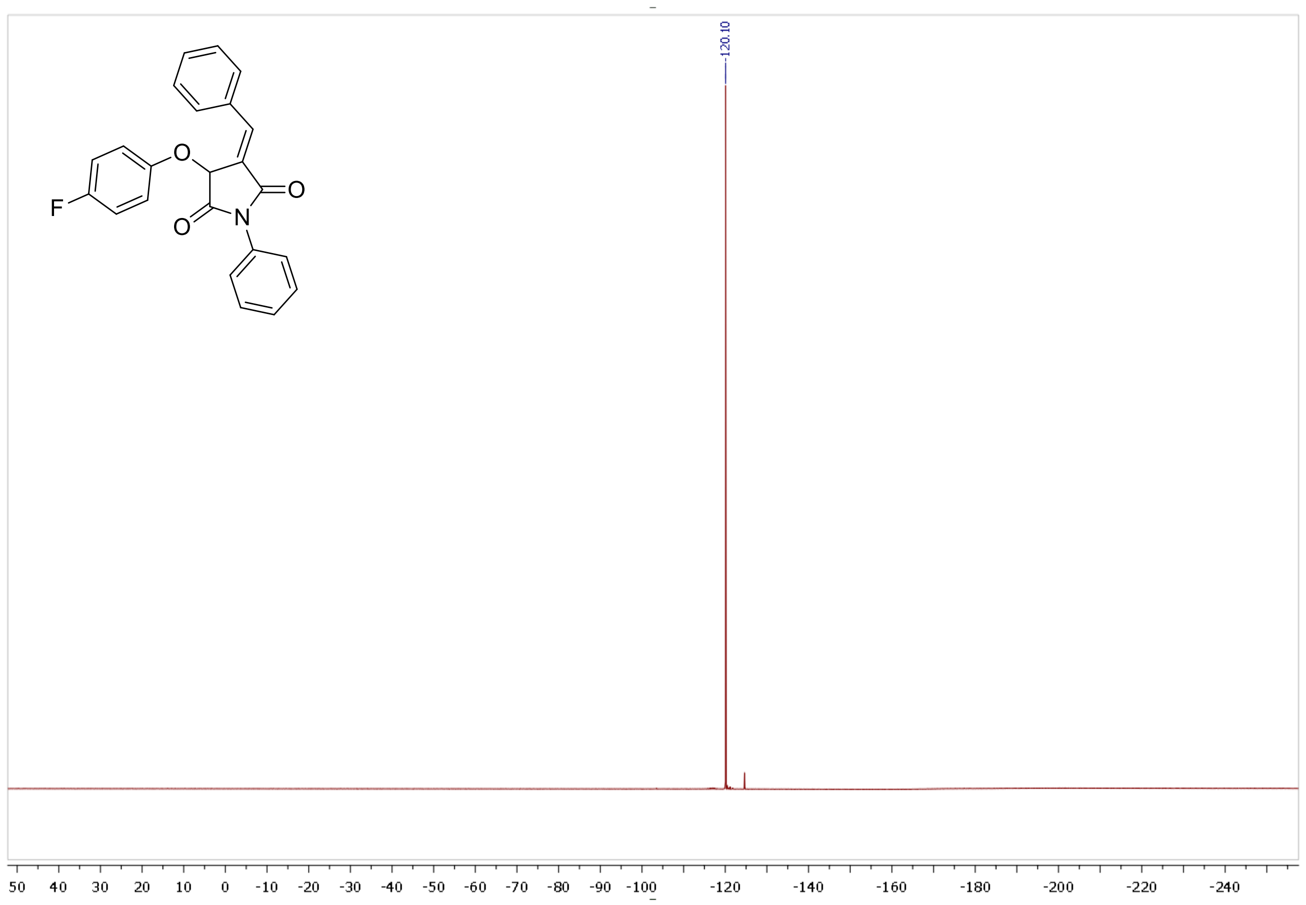

Copies of ^{1}H (400.13 MHz, $CDCl_3$) and ^{13}C (100.61 MHz, $CDCl_3$) spectra of **5c**

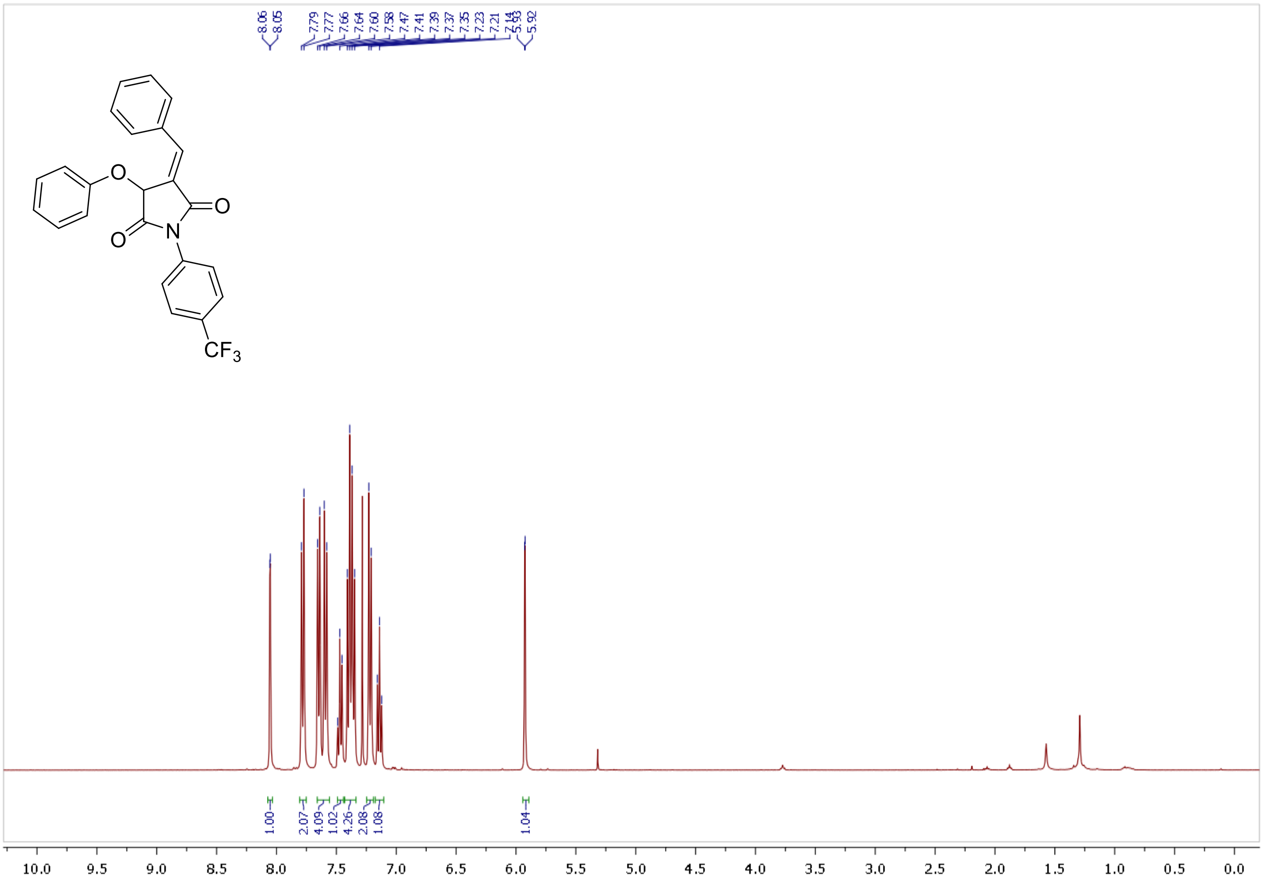

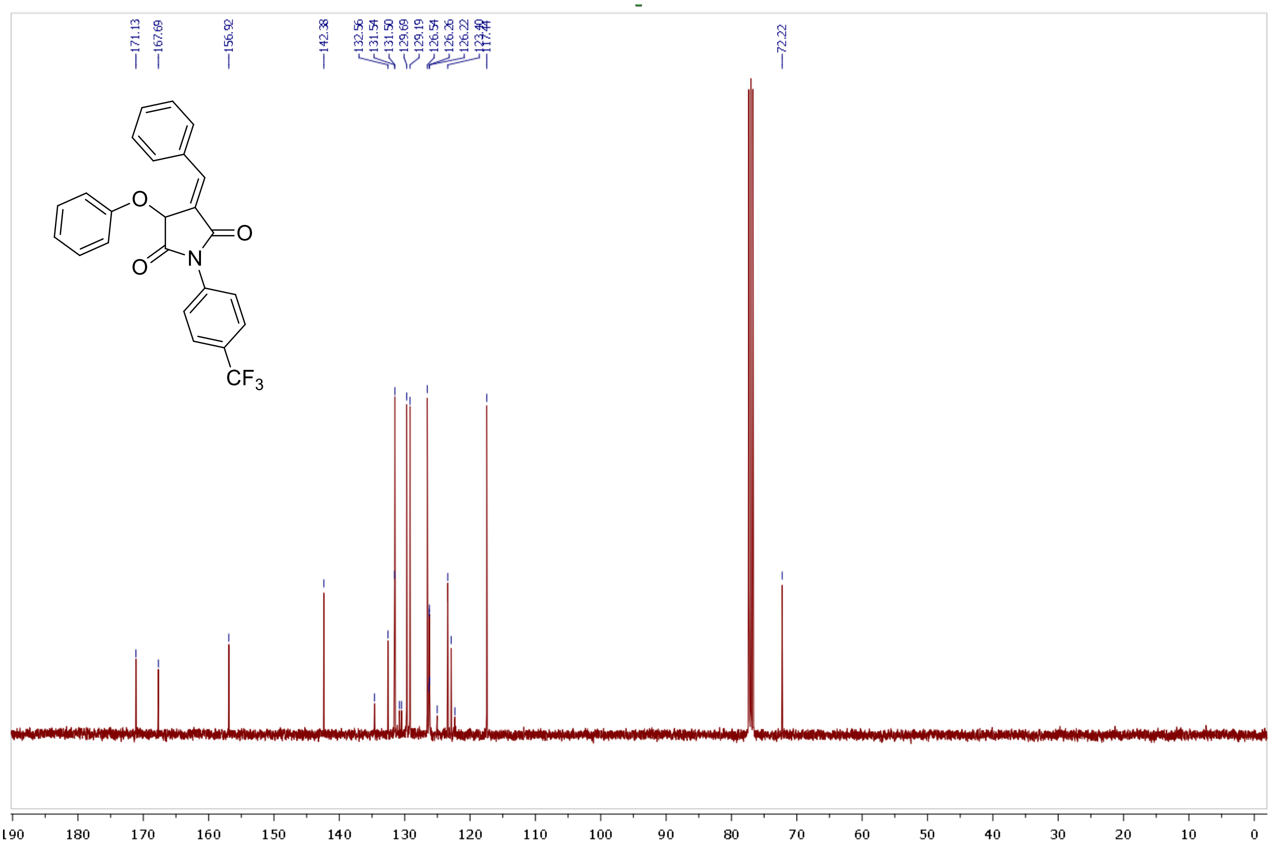

Copy of ^{19}F (376.50 MHz, $CDCl_3$) spectrum of **5c**

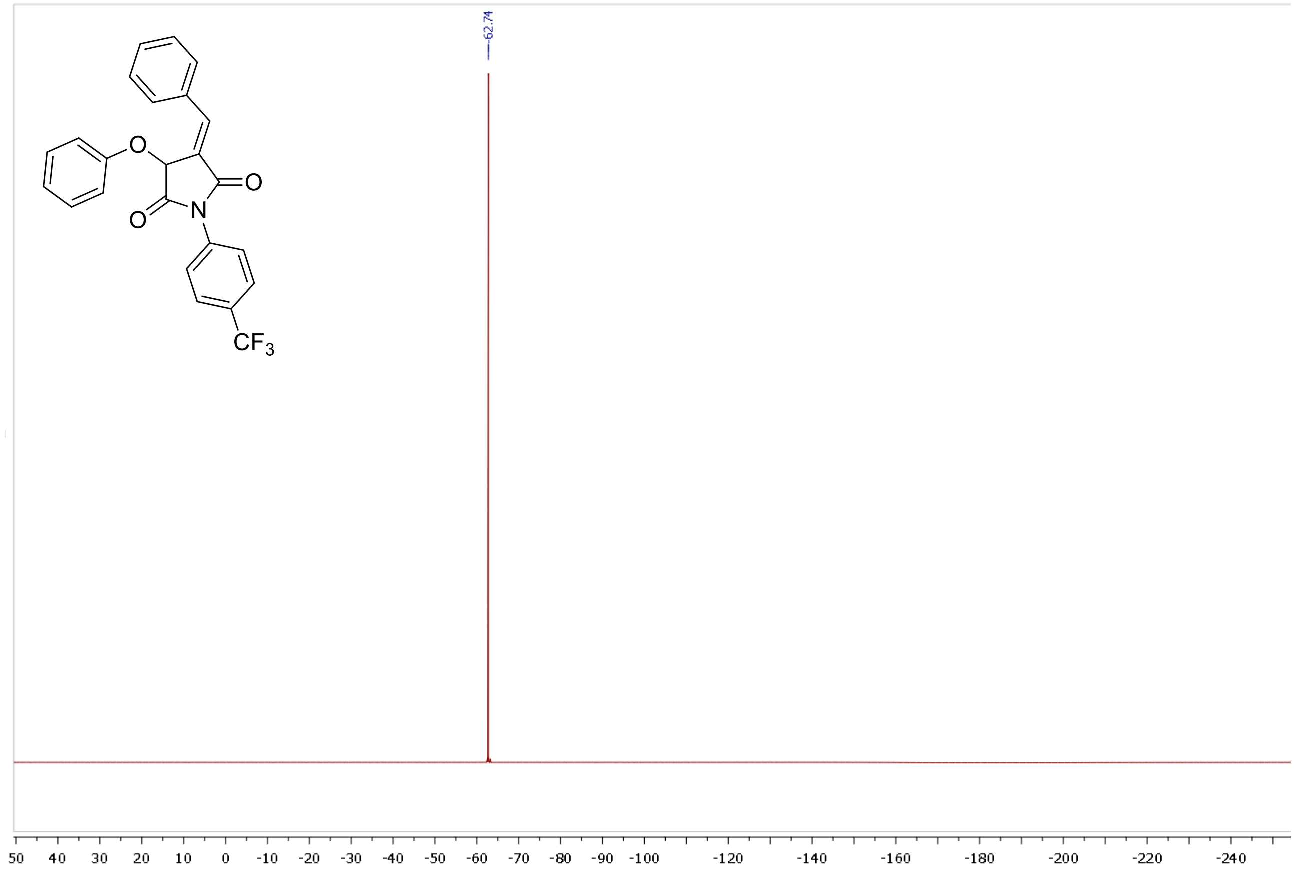

Copies of ^{1}H (400.13 MHz, $CDCl_3$) and ^{13}C (100.61 MHz, $CDCl_3$) spectra of **5d**

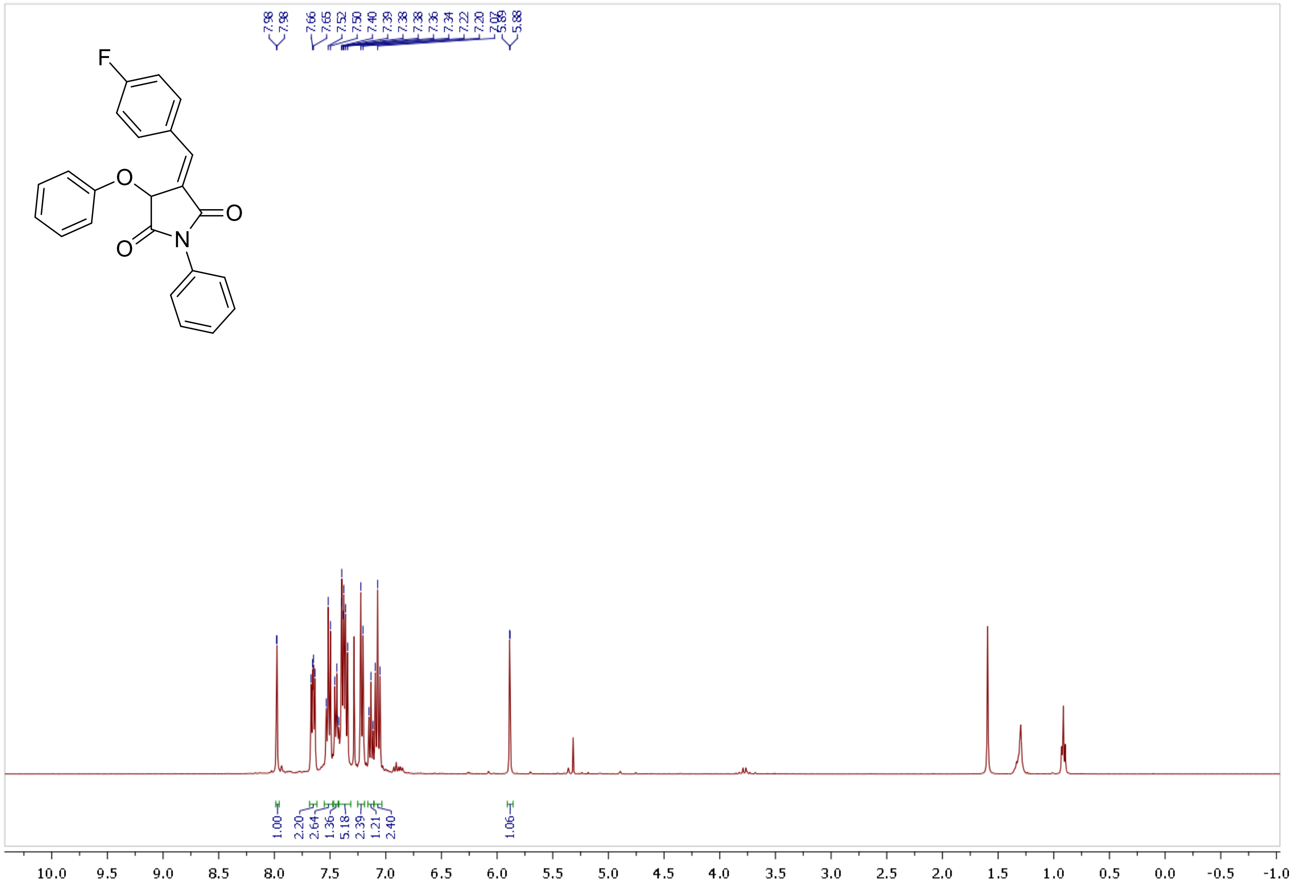

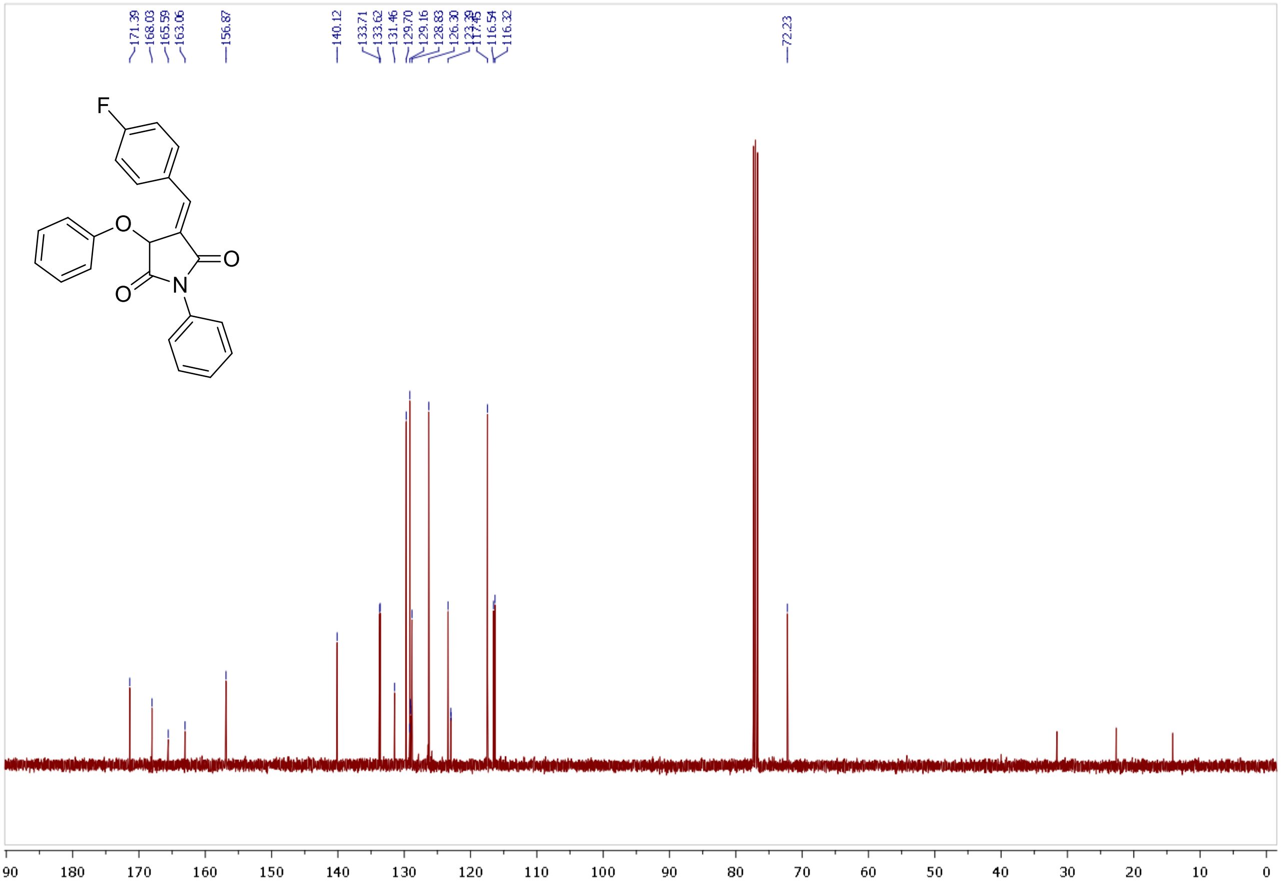

Copy of ^{19}F (376.50 MHz, $CDCl_3$) spectrum of **5d**

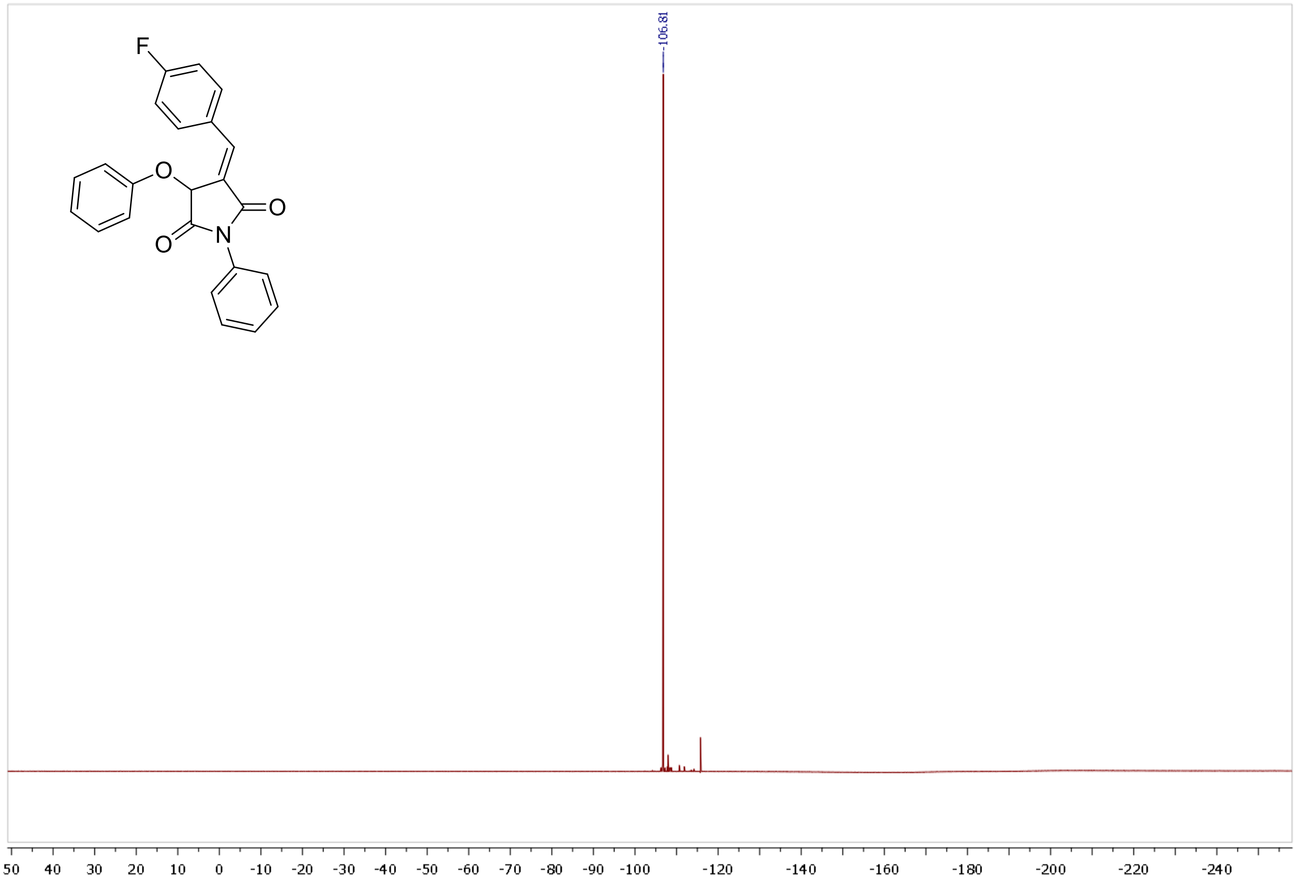

Copies of ^{1}H (400.13 MHz, $CDCl_3$) and ^{13}C (100.61 MHz, $CDCl_3$) spectra of **5e**

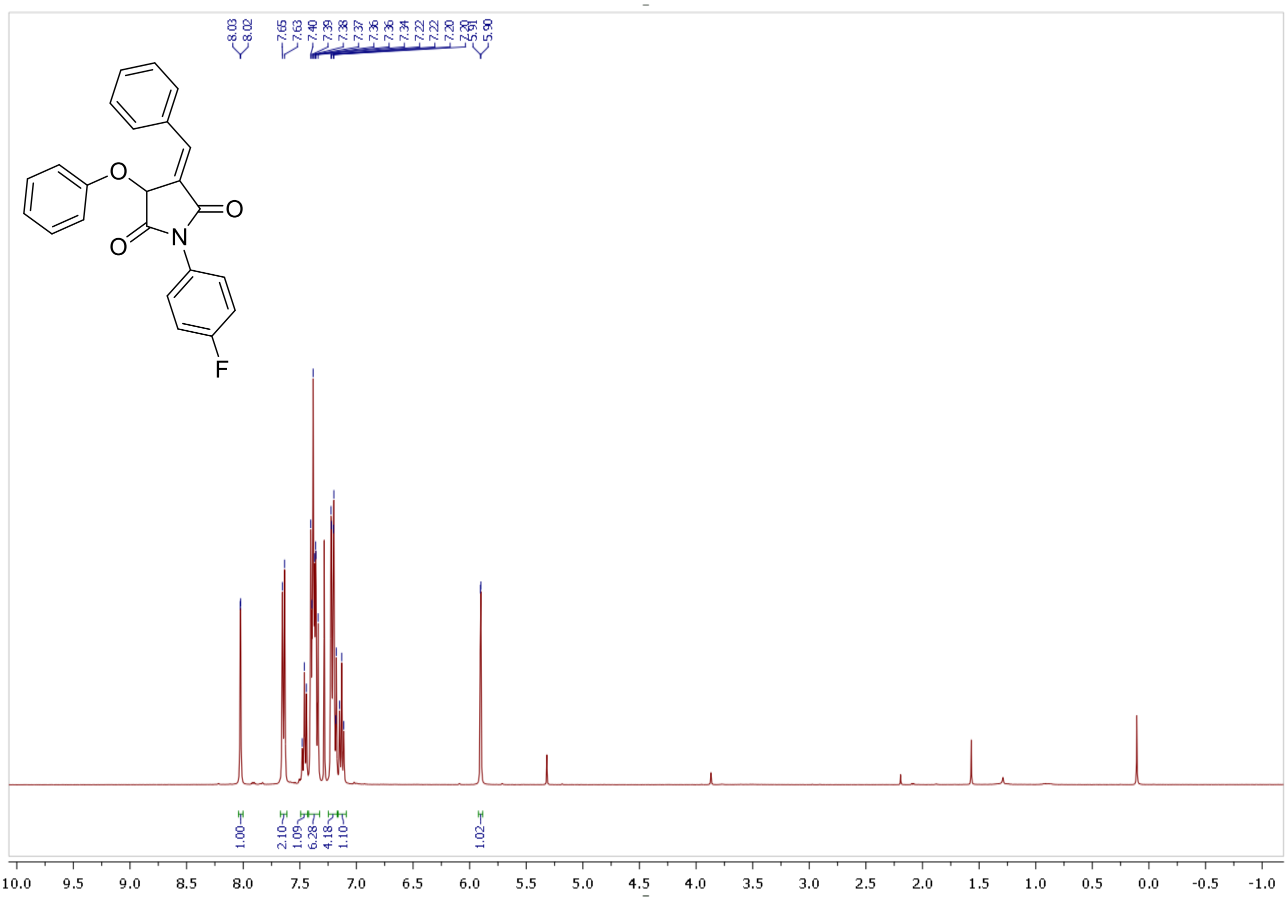

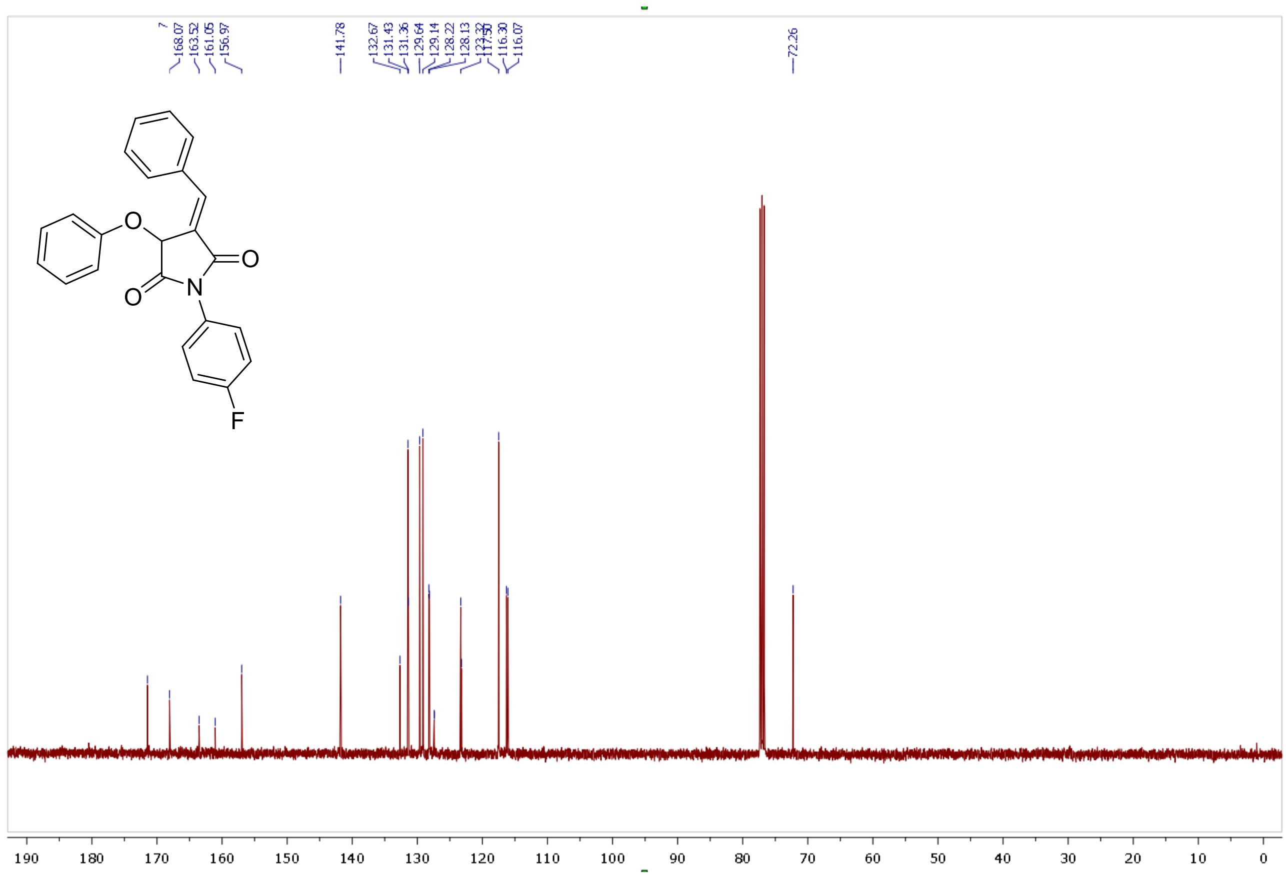

Copy of ^{19}F (376.50 MHz, $CDCl_3$) spectrum of **5e**

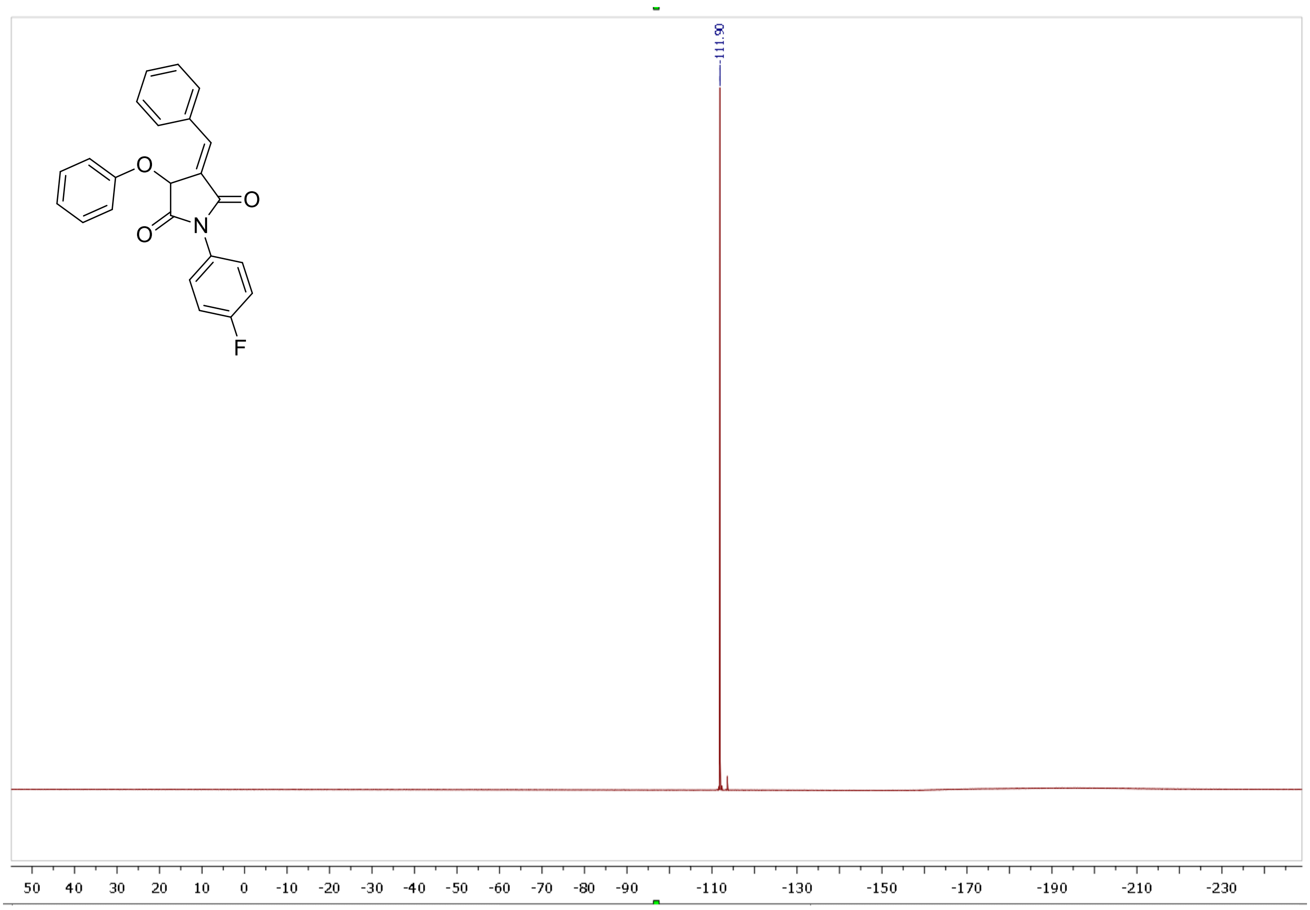

Copies of ^{1}H (400.13 MHz, $CDCl_3$) and ^{13}C (100.61 MHz, $CDCl_3$) spectra of **5f**

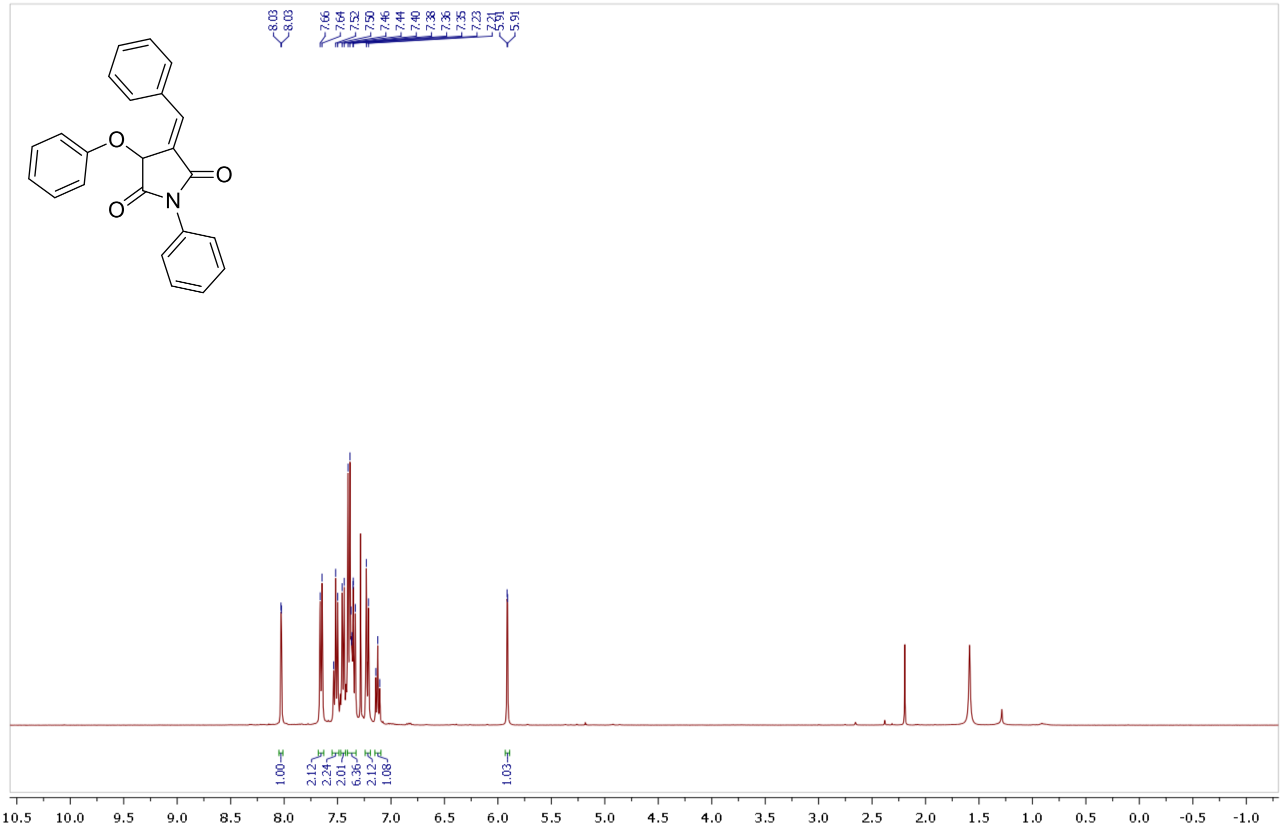

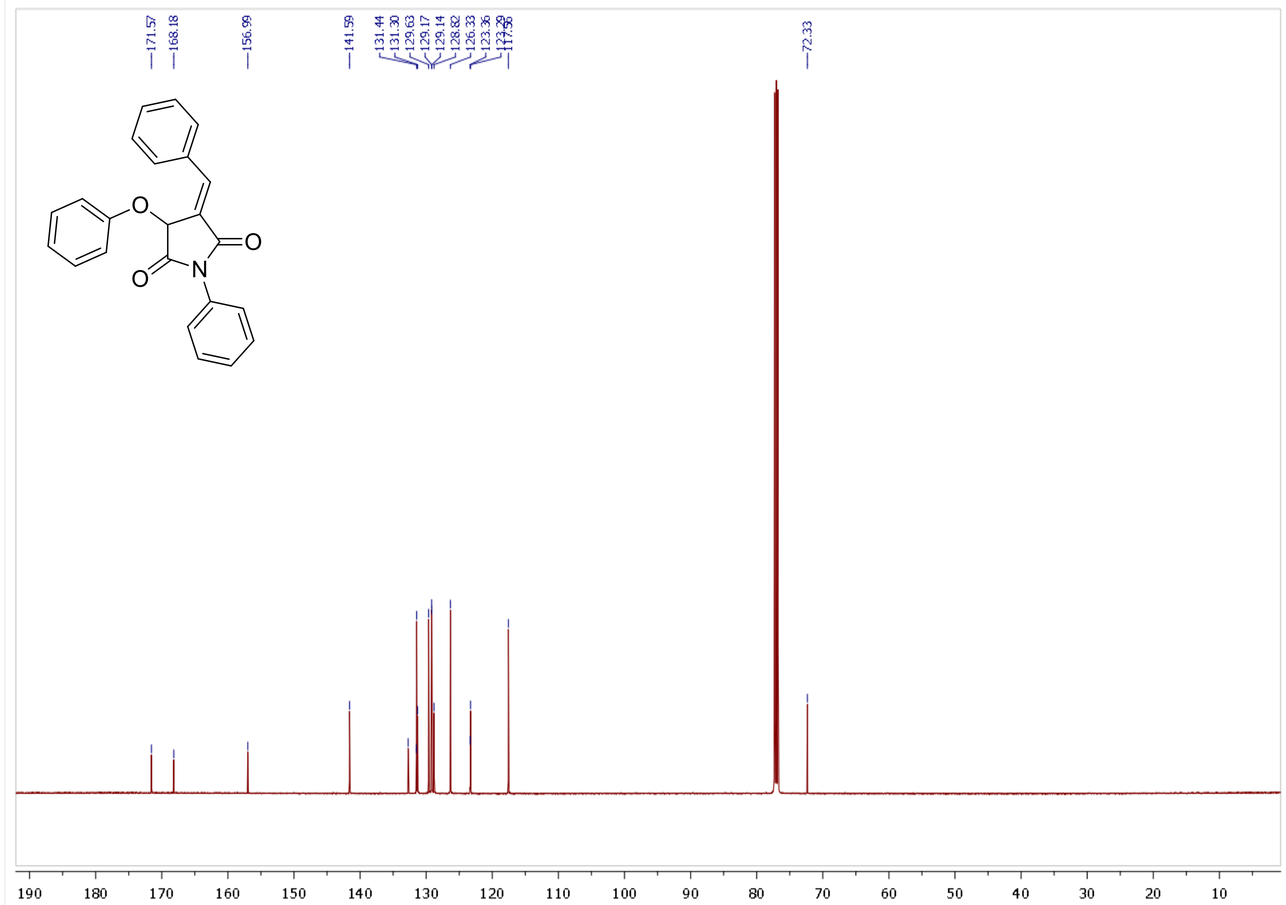

Copies of ^{1}H (400.13 MHz, $CDCl_3$) and ^{13}C (100.61 MHz, $CDCl_3$) spectra of **5g**

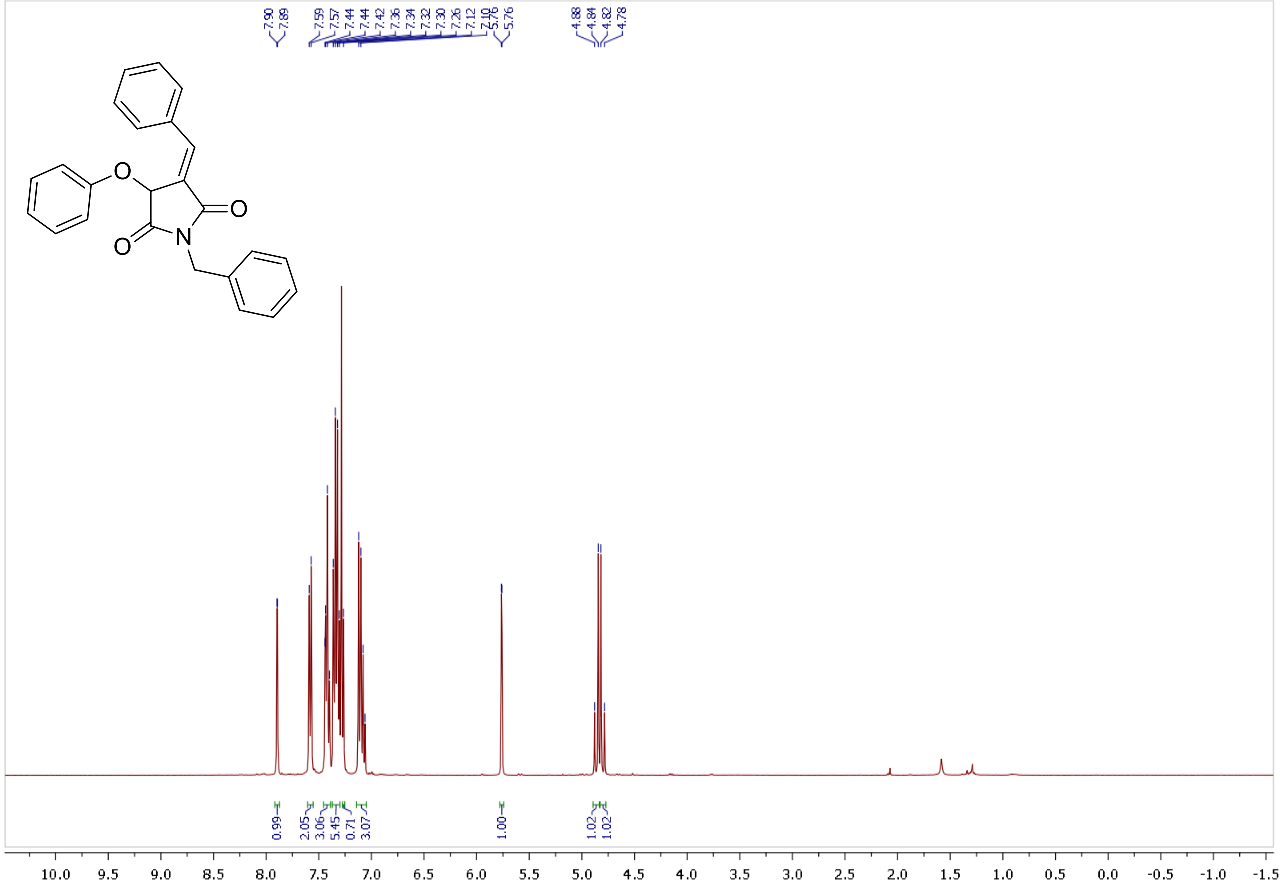

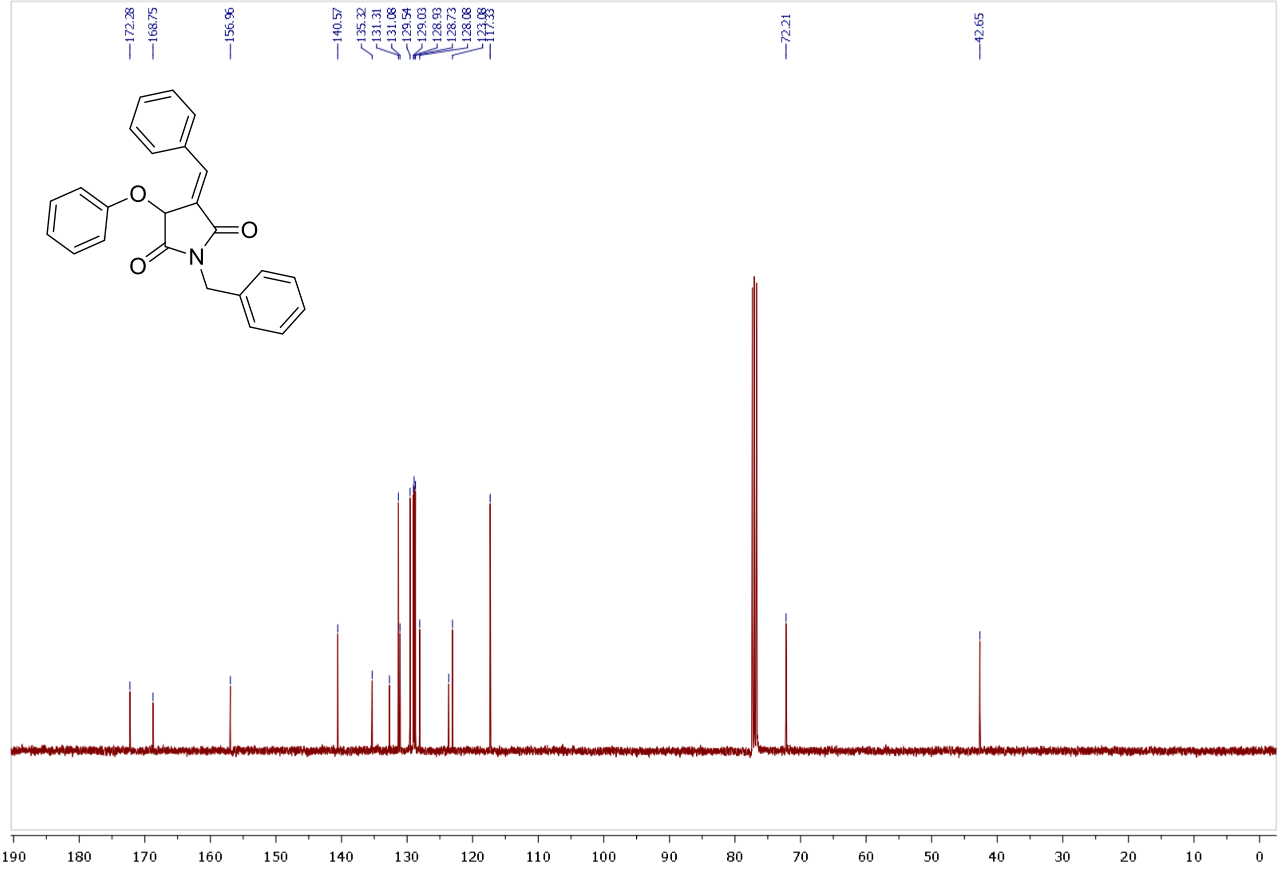

Copies of ^{1}H (400.13 MHz, $CDCl_3$) and ^{13}C (100.61 MHz, $CDCl_3$) spectra of **5h**

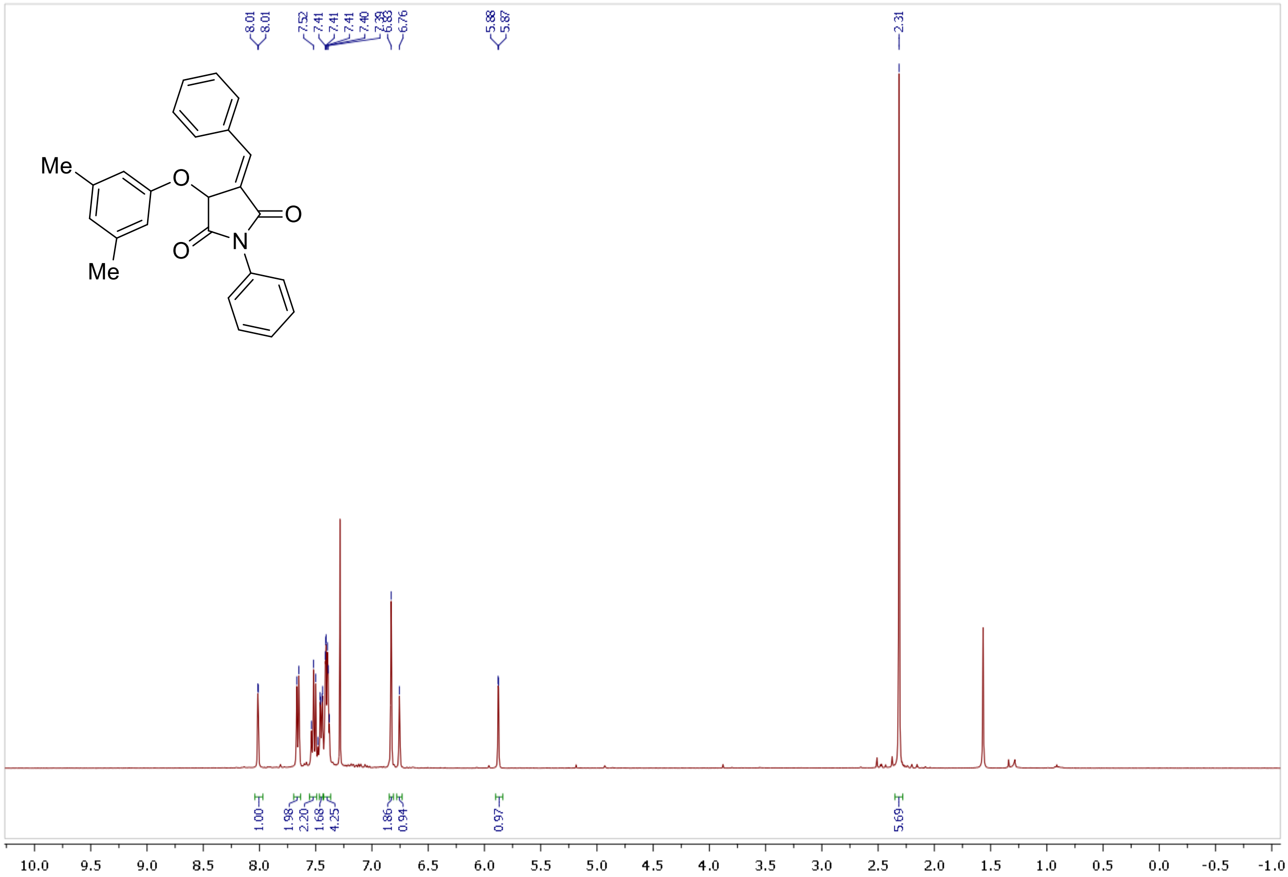

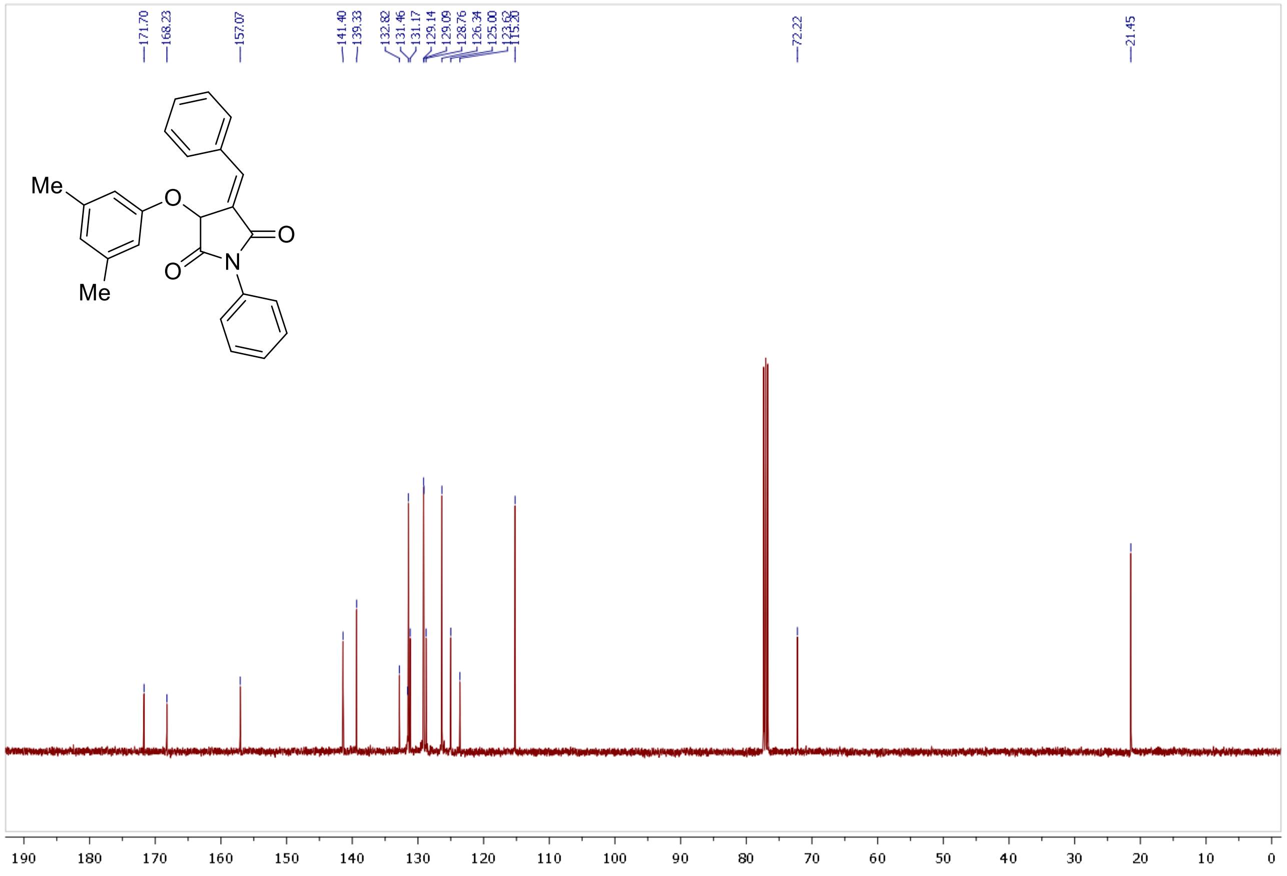

Copies of ^{1}H (400.13 MHz, $CDCl_3$) and ^{13}C (100.61 MHz, $CDCl_3$) spectra of **5i**

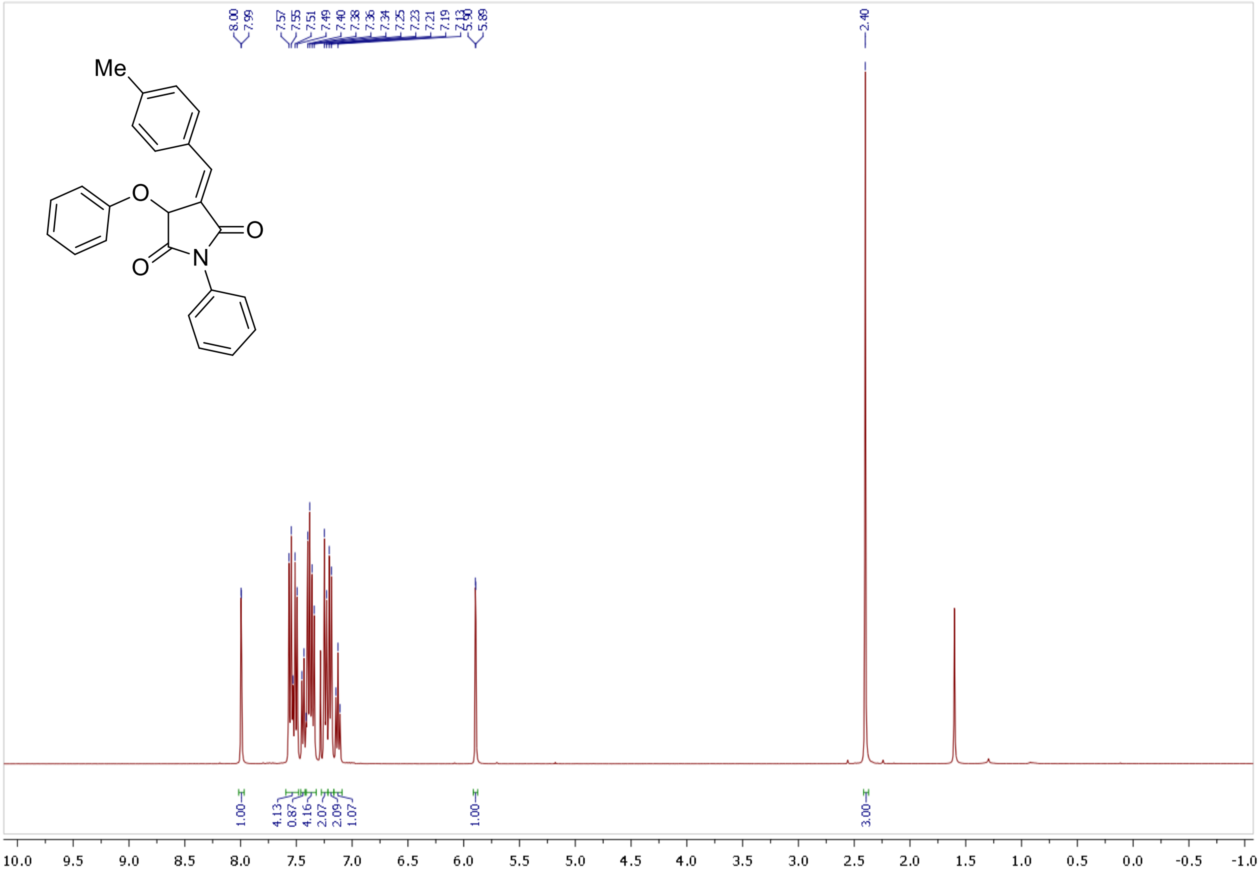

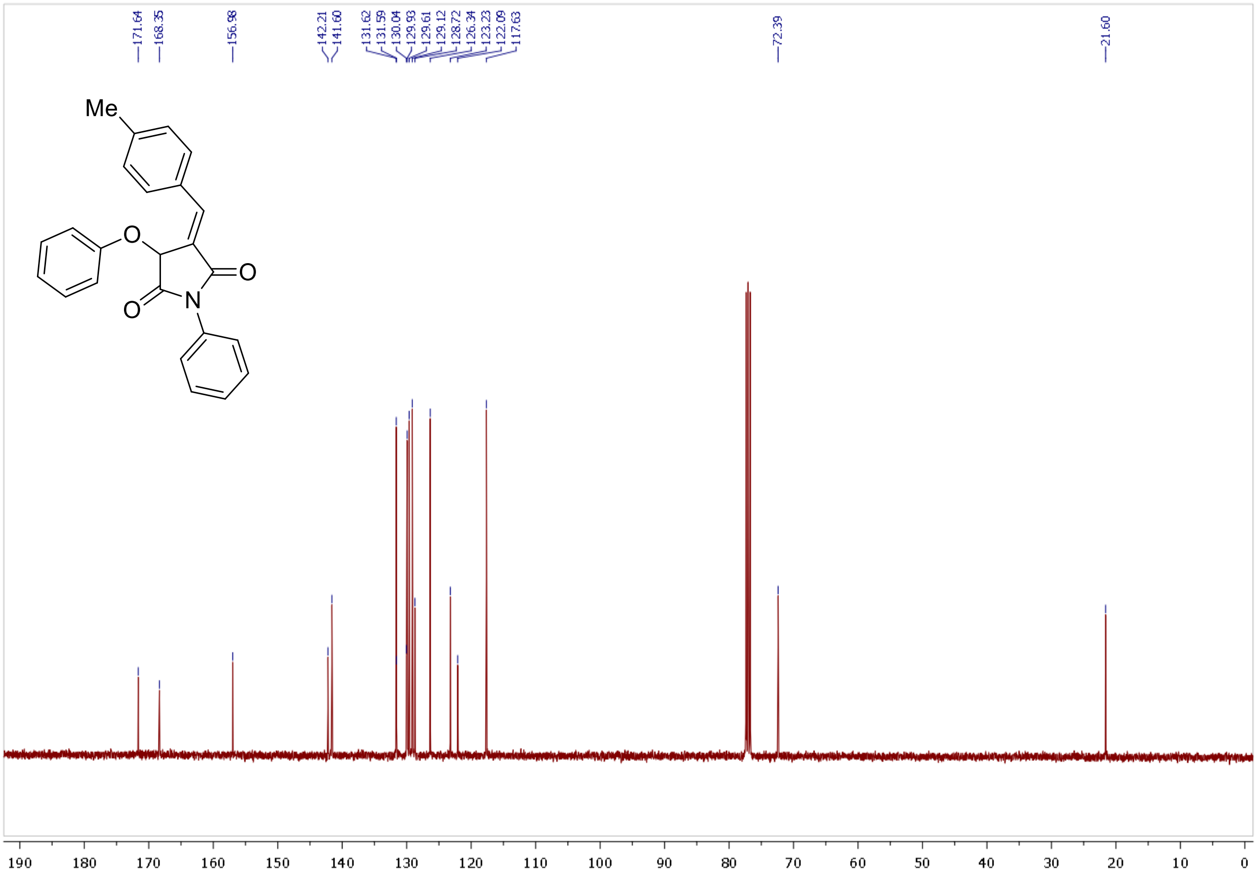

Copies of ^{1}H (400.13 MHz, $CDCl_3$) and ^{13}C (100.61 MHz, $CDCl_3$) spectra of **5j**

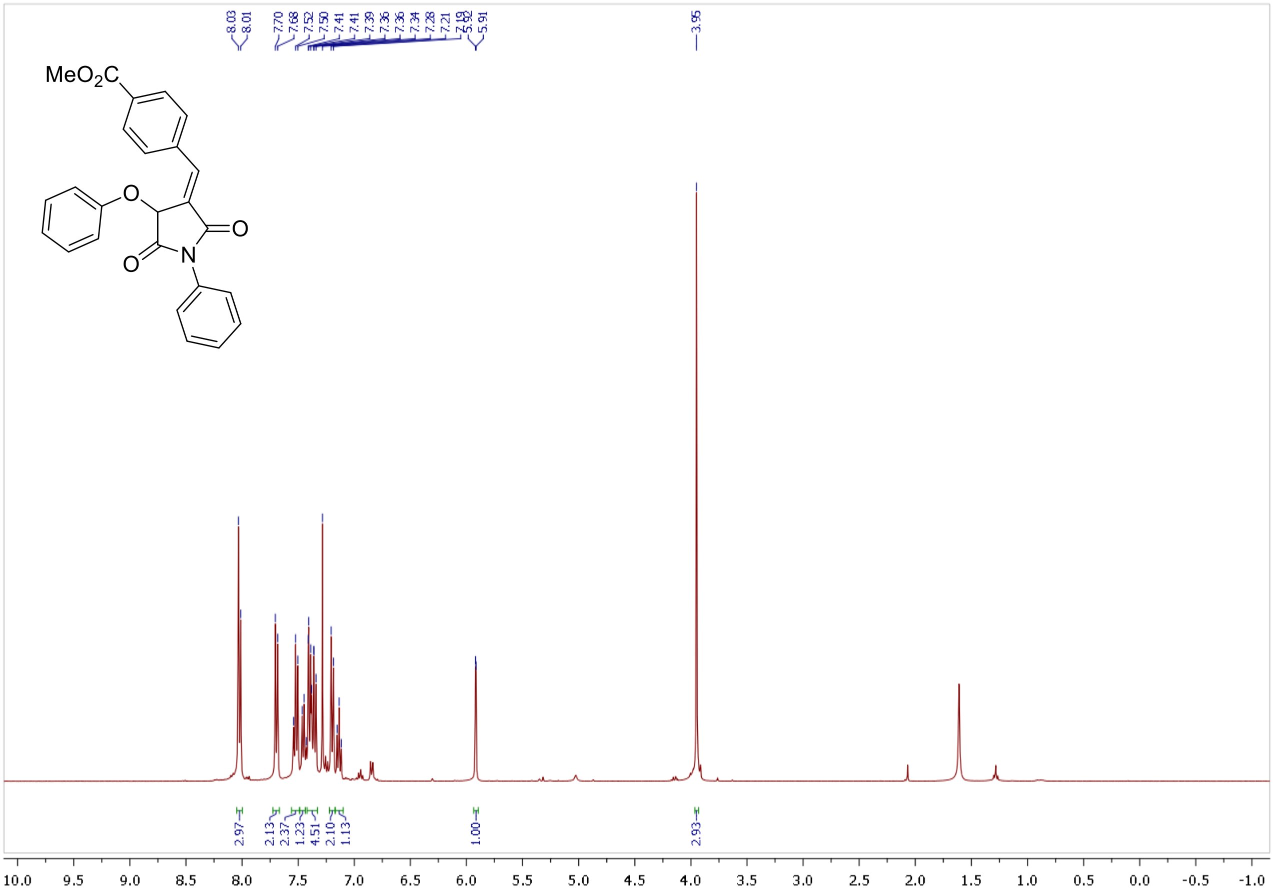

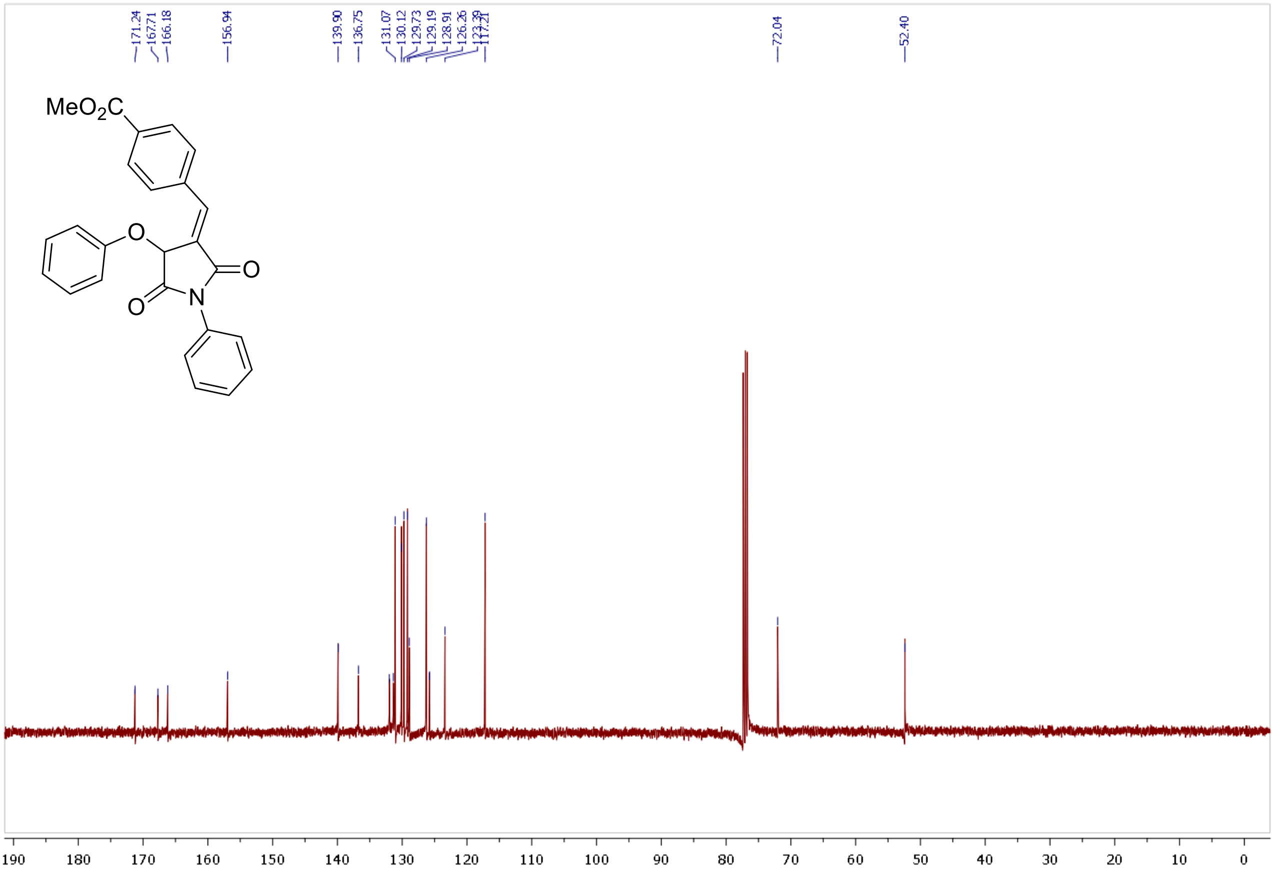

Copies of ^{1}H (400.13 MHz, CDCl$_3$) and ^{13}C (100.61 MHz, CDCl$_3$) spectra of **5k**

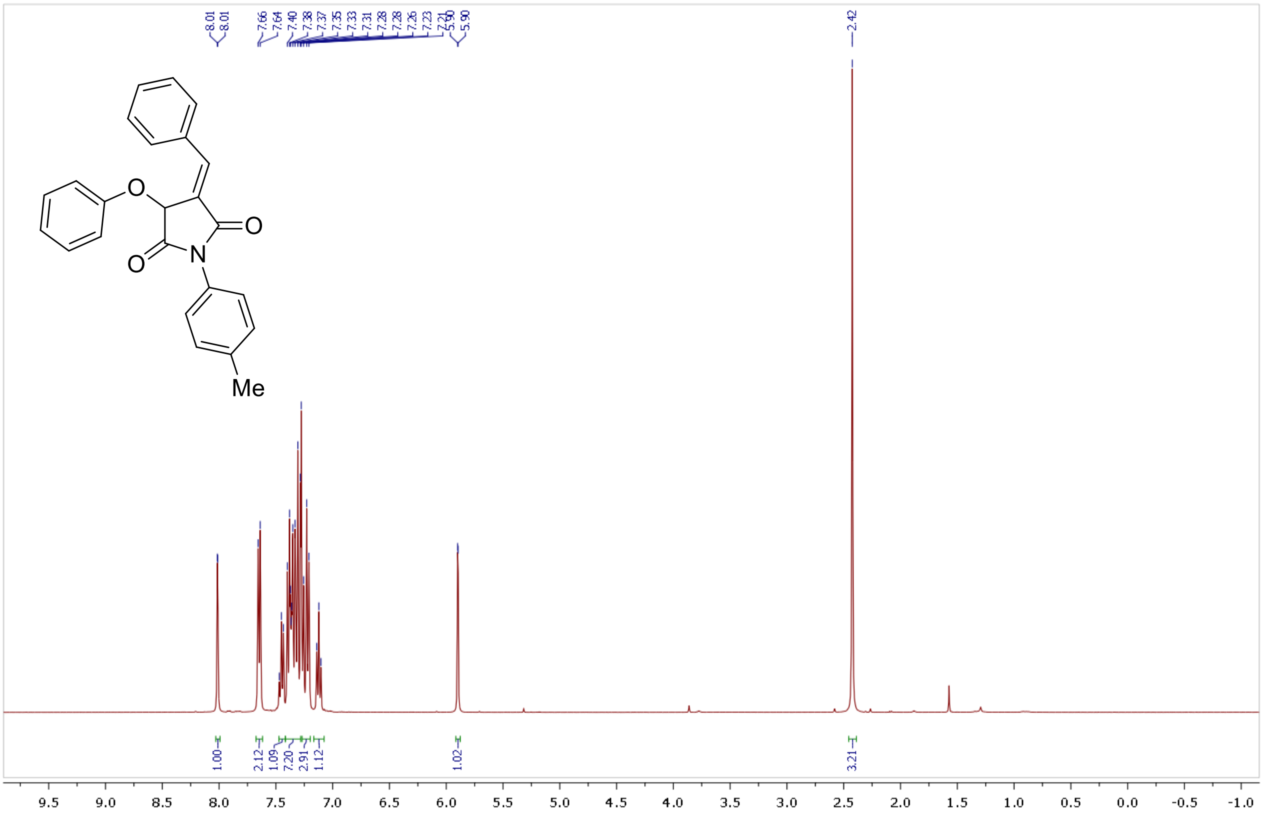

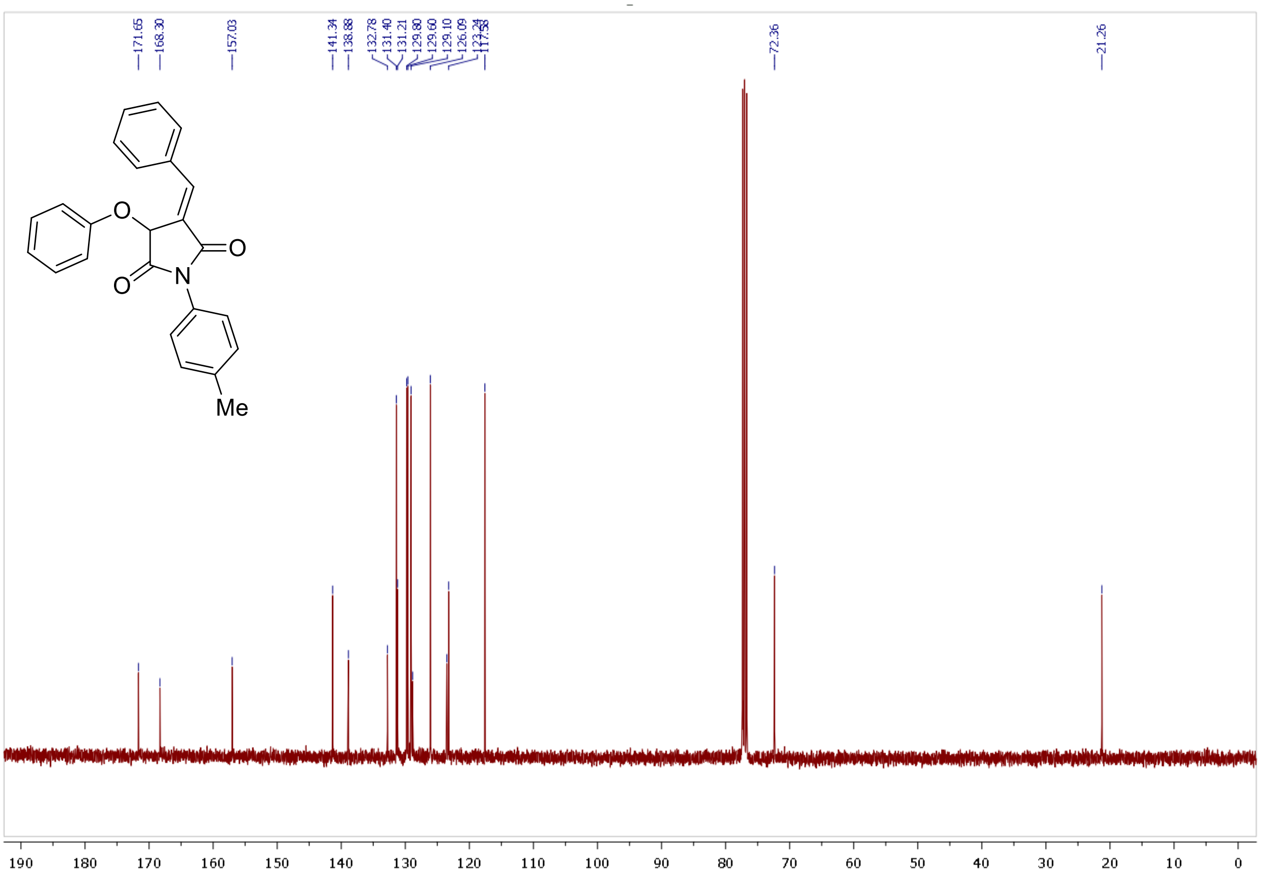

Copies of ^{1}H (400.13 MHz, $CDCl_3$) and ^{13}C (100.61 MHz, $CDCl_3$) spectra of **5l**

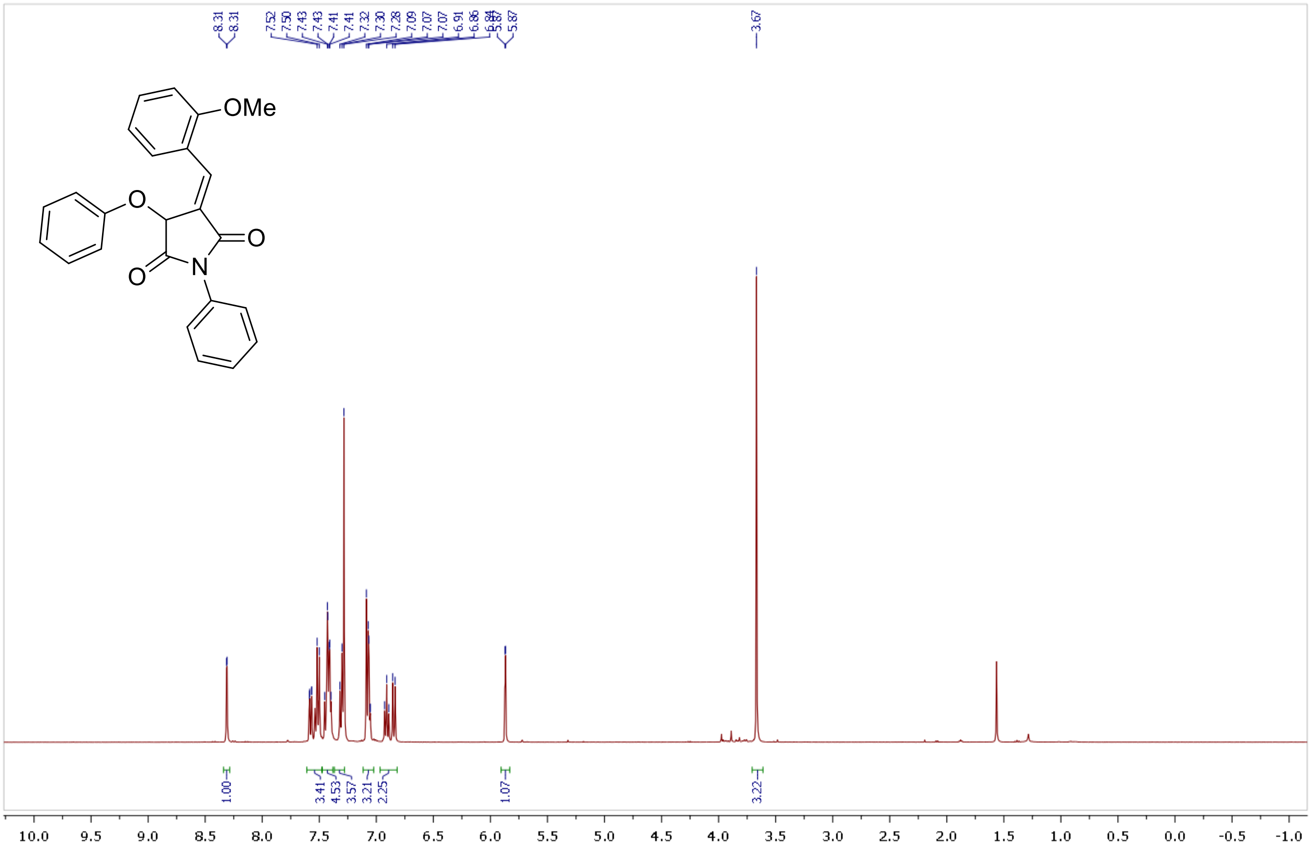

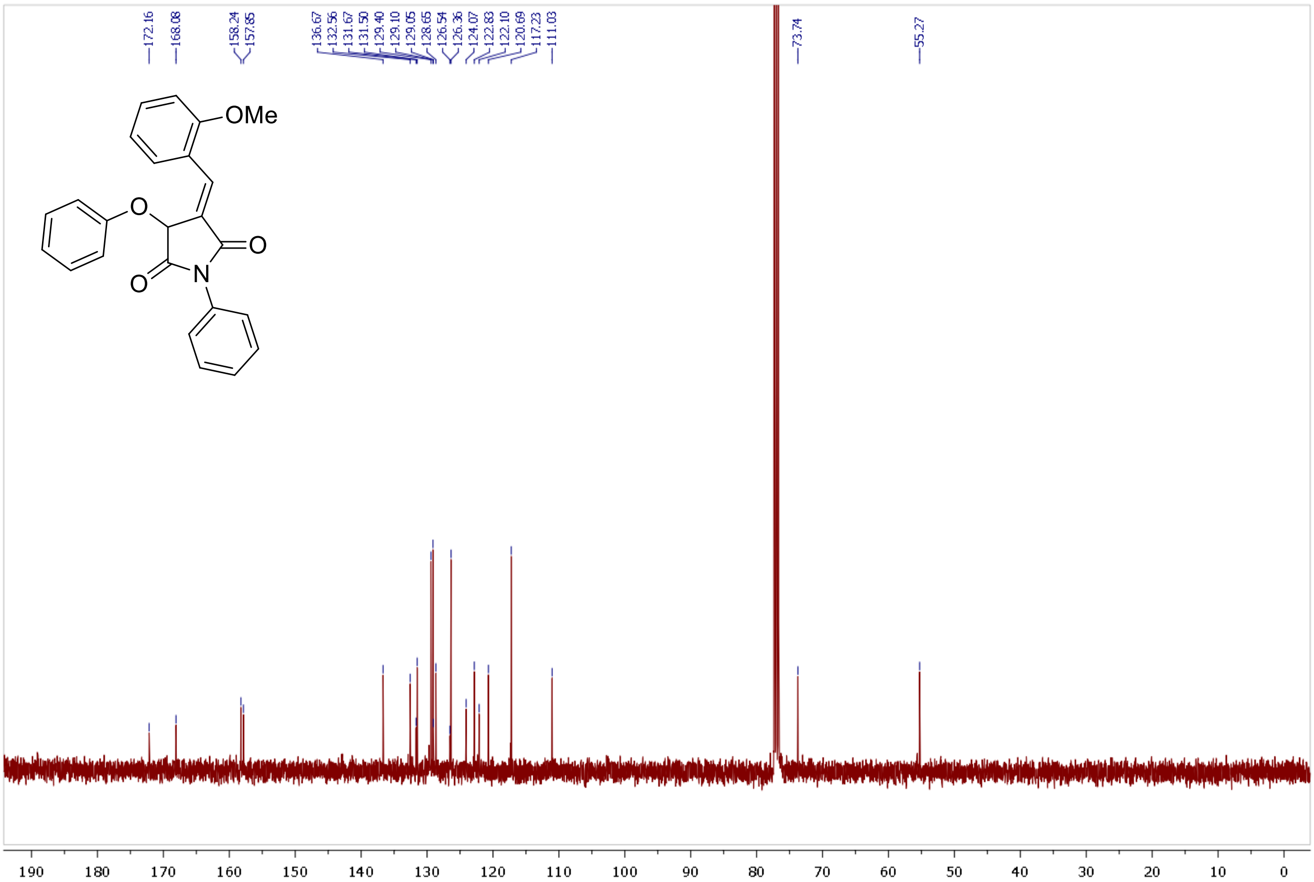

Copies of ^{1}H (400.13 MHz, $CDCl_3$) and ^{13}C (100.61 MHz, $CDCl_3$) spectra of **5m**

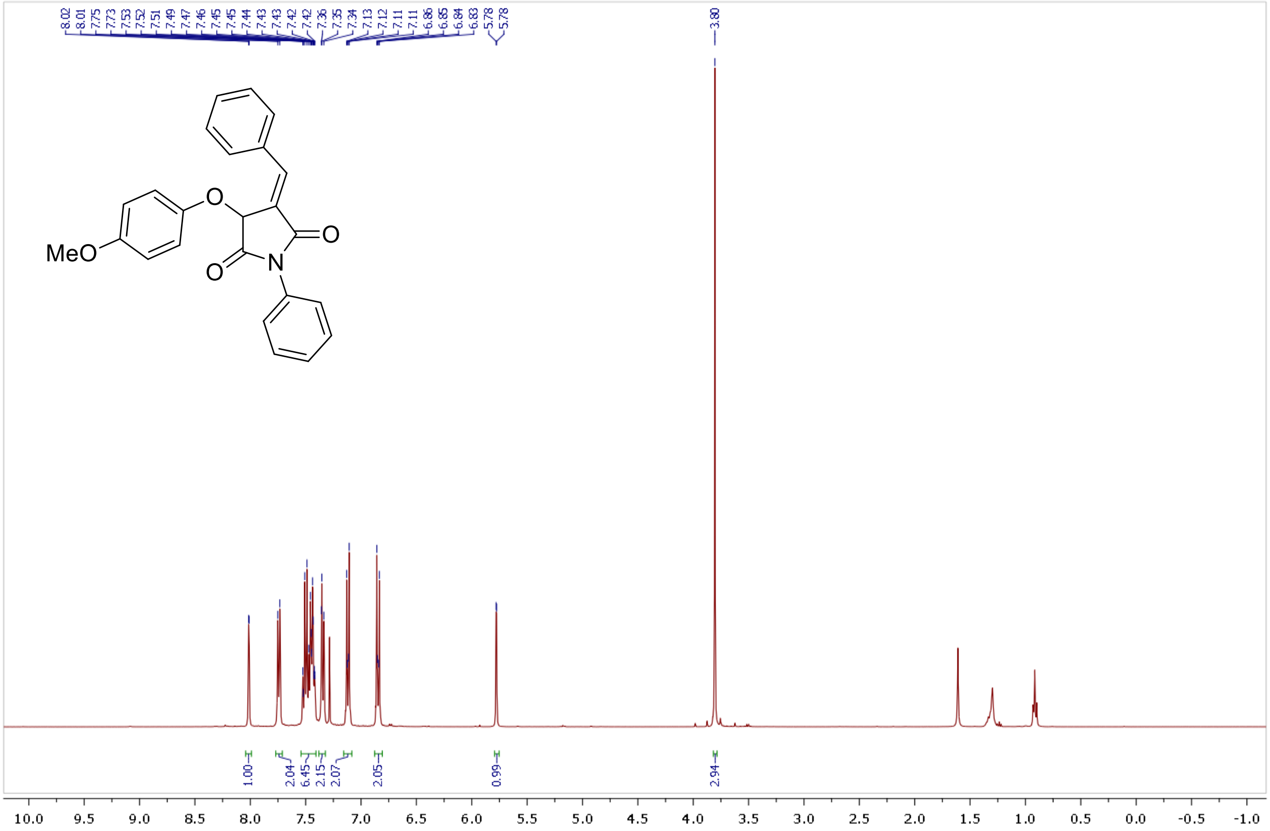

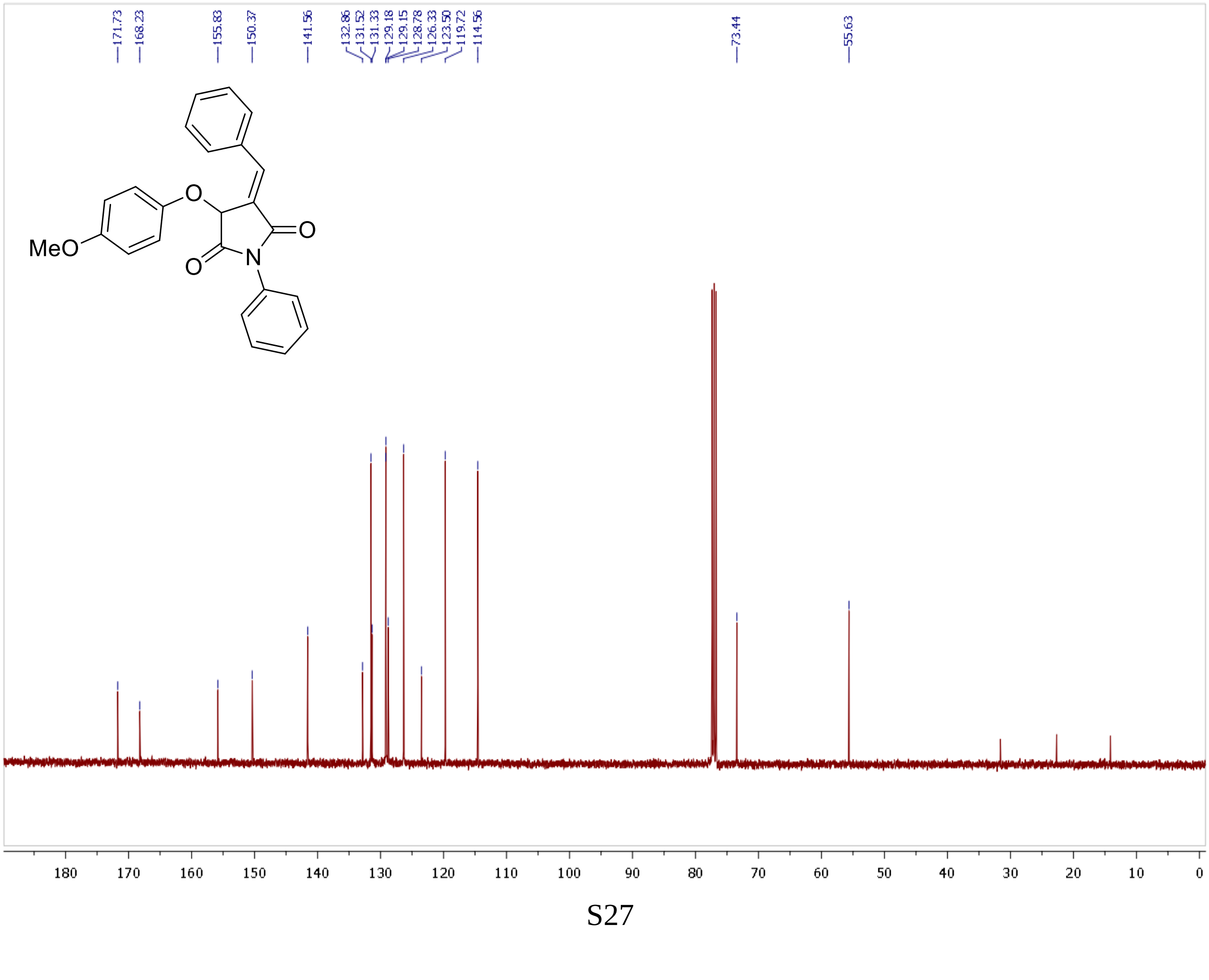

Copies of ^{1}H (400.13 MHz, CDCl$_3$) and ^{13}C (100.61 MHz, CDCl$_3$) spectra of **5n**

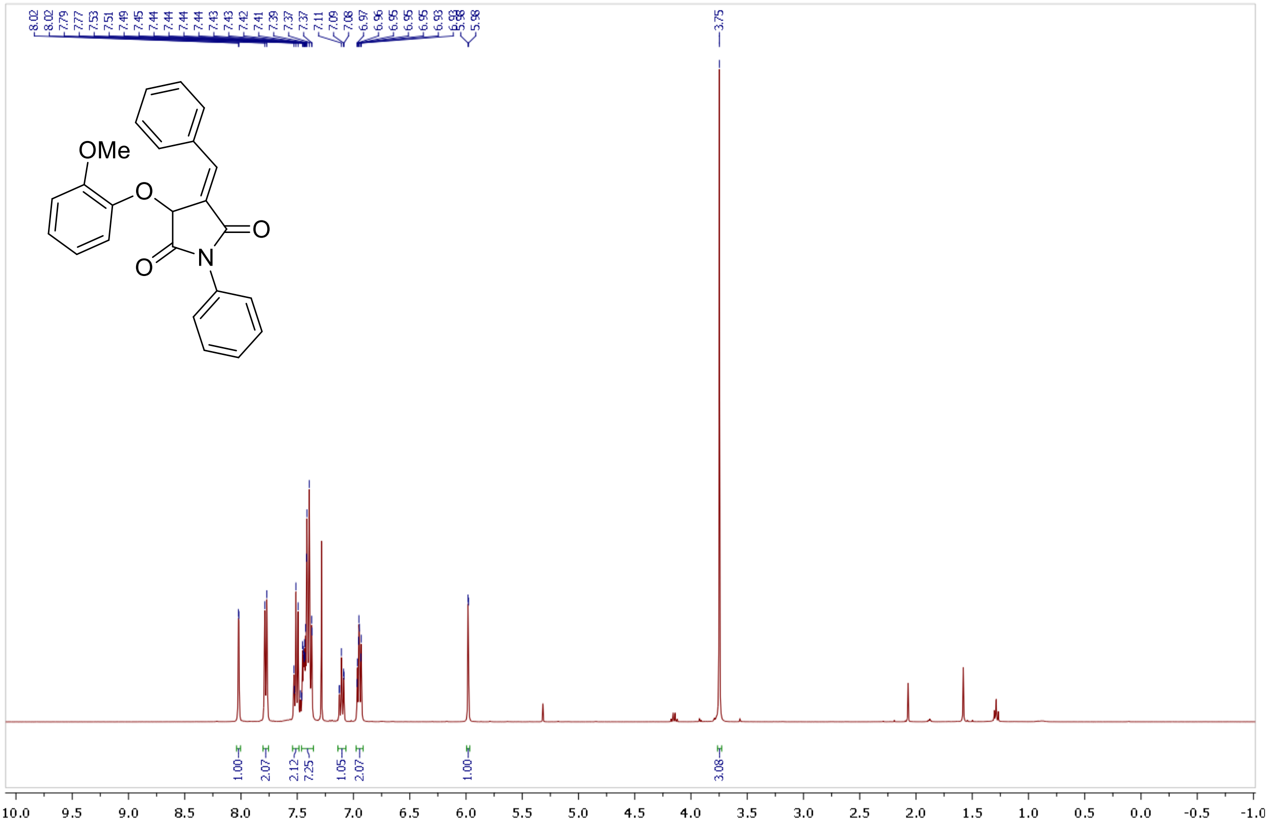

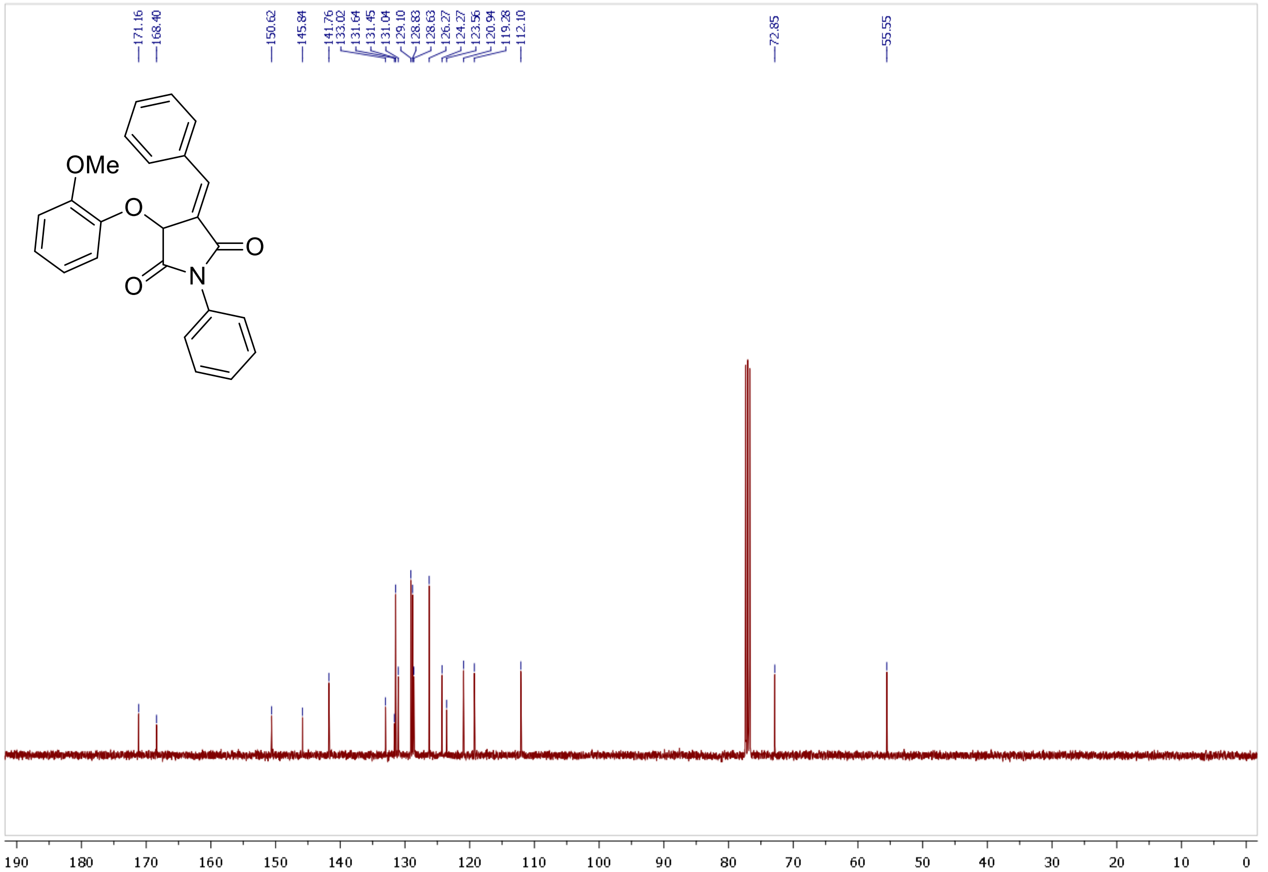

Copies of ^{1}H (400.13 MHz, $CDCl_3$) and ^{13}C (100.61 MHz, $CDCl_3$) spectra of **6a**

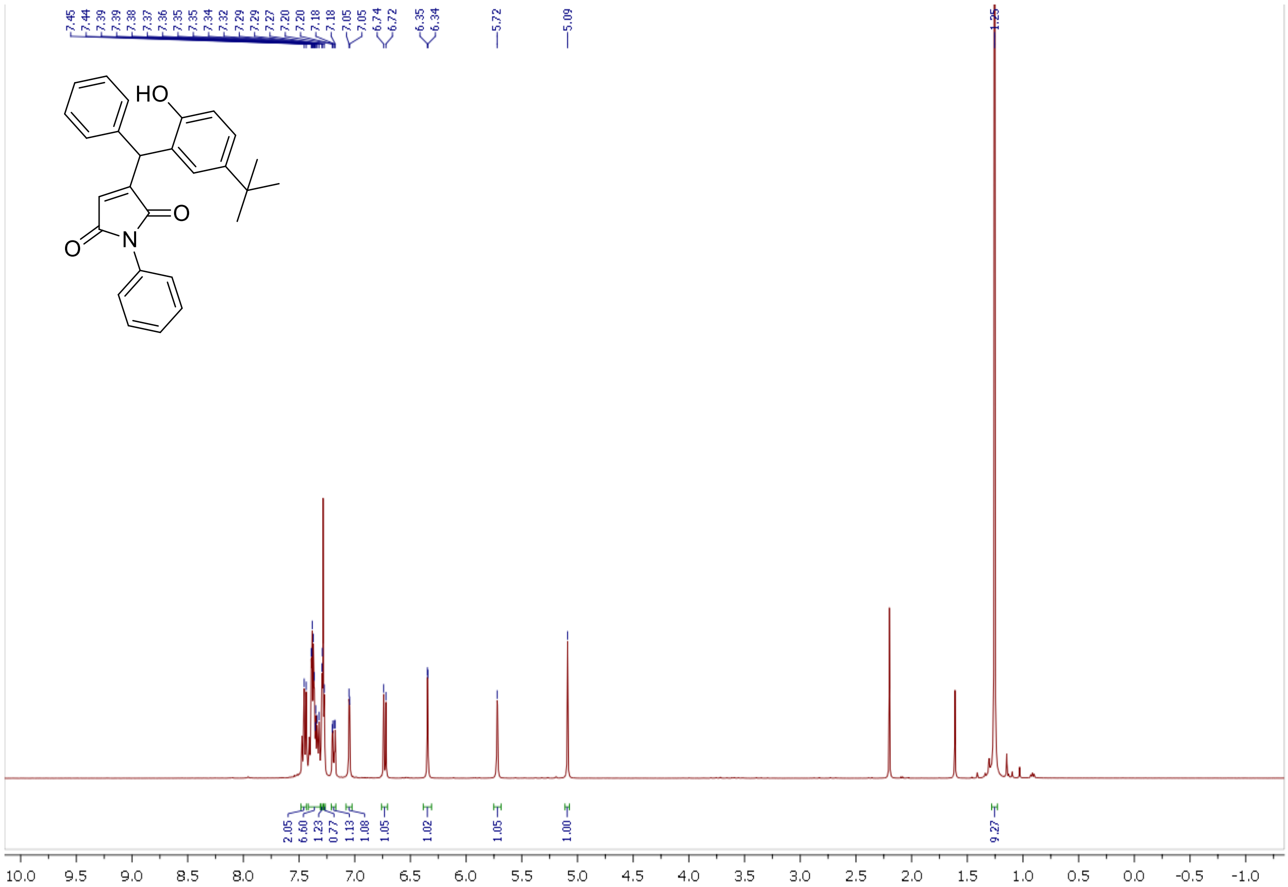

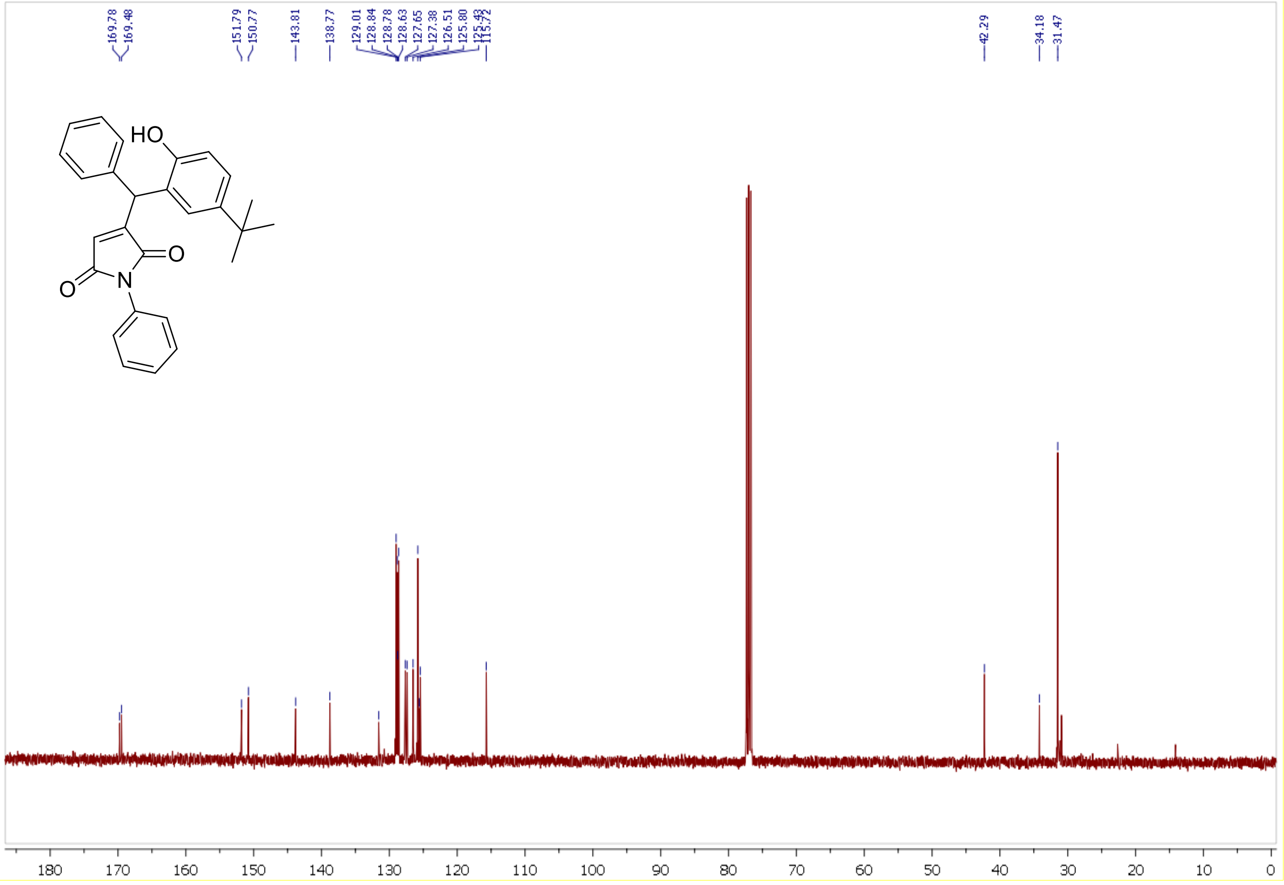

Copies of ^{1}H (400.13 MHz, $CDCl_3$) and ^{13}C (100.61 MHz, $CDCl_3$) spectra of *syn*-**7a**

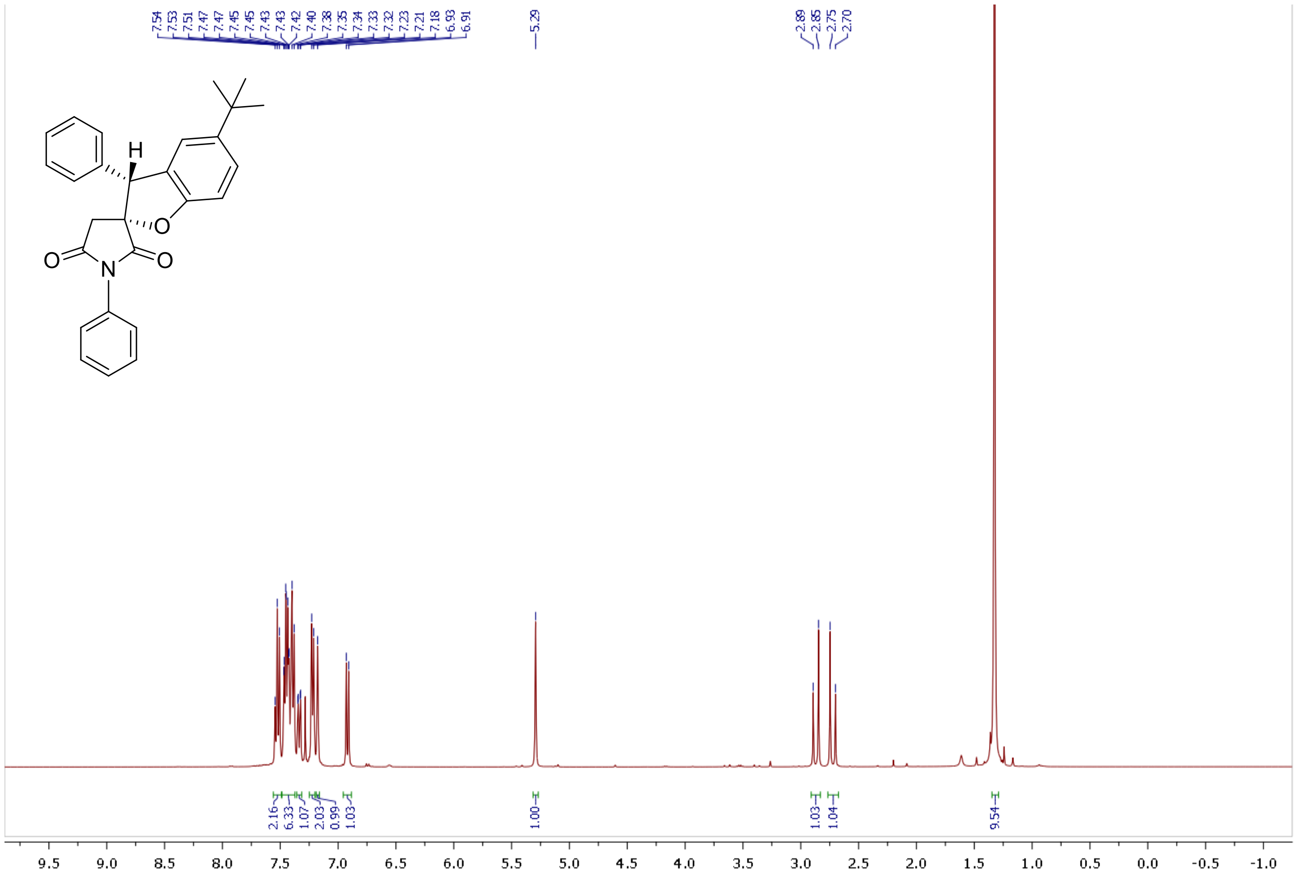

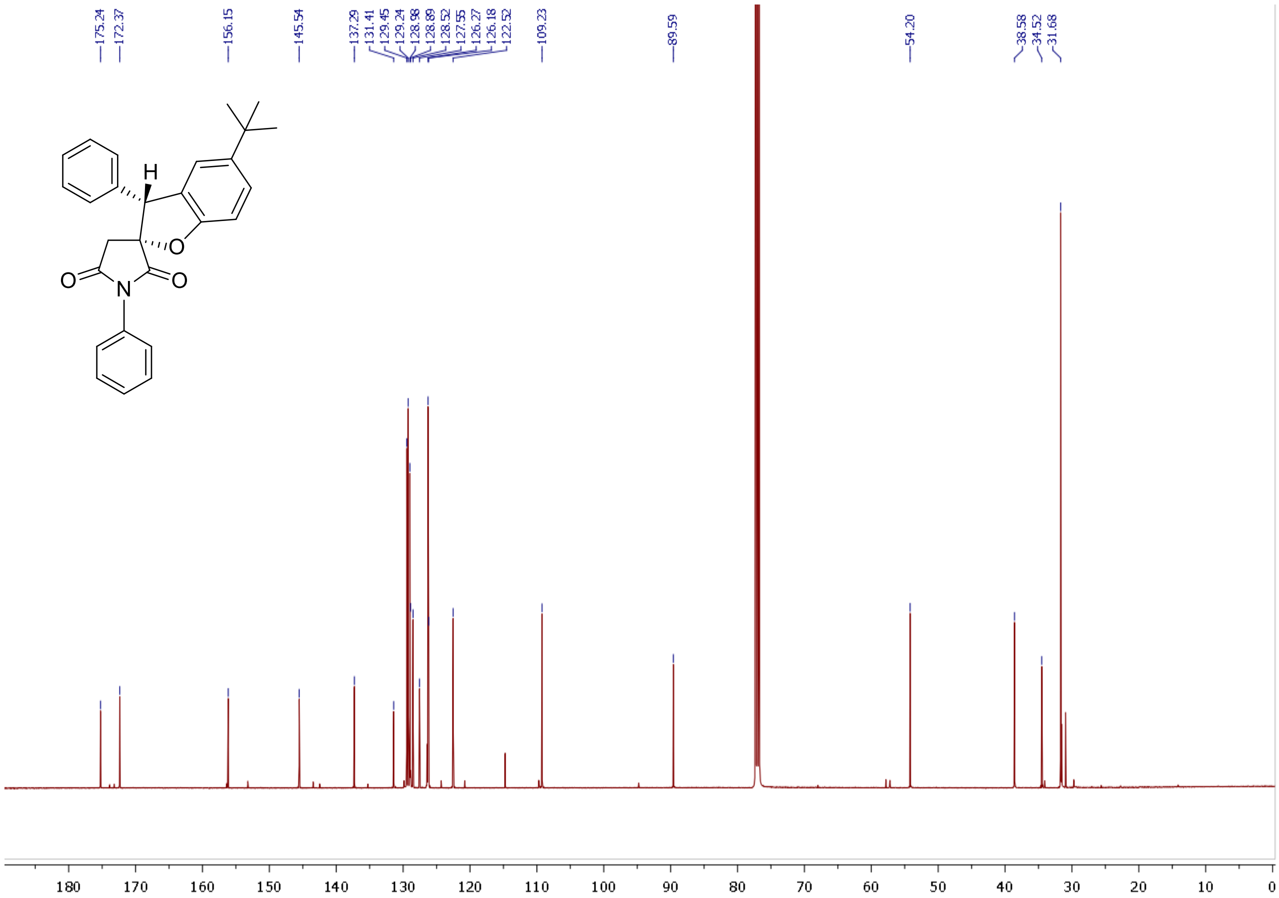

Copies of ^{1}H (400.13 MHz, $CDCl_3$) and ^{13}C (100.61 MHz, $CDCl_3$) spectra of *anti*-**7a**

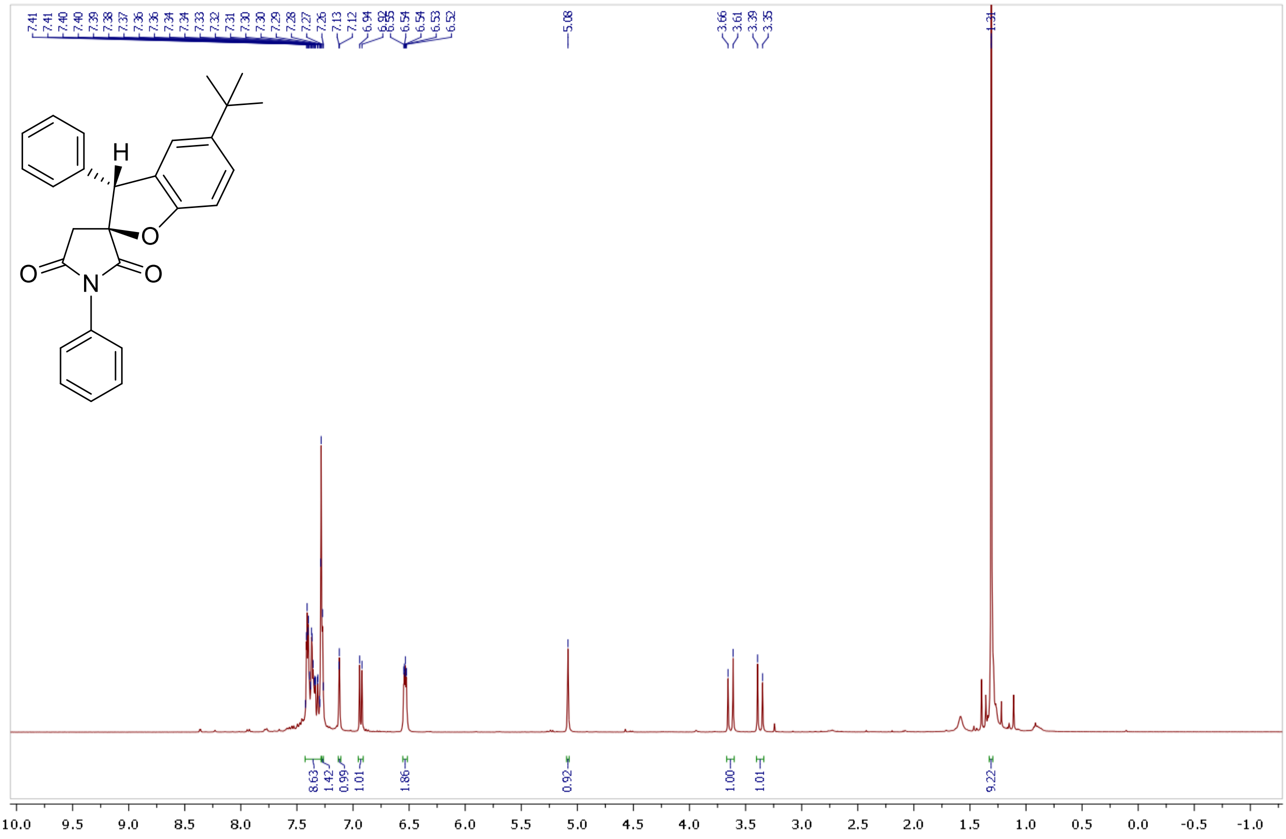

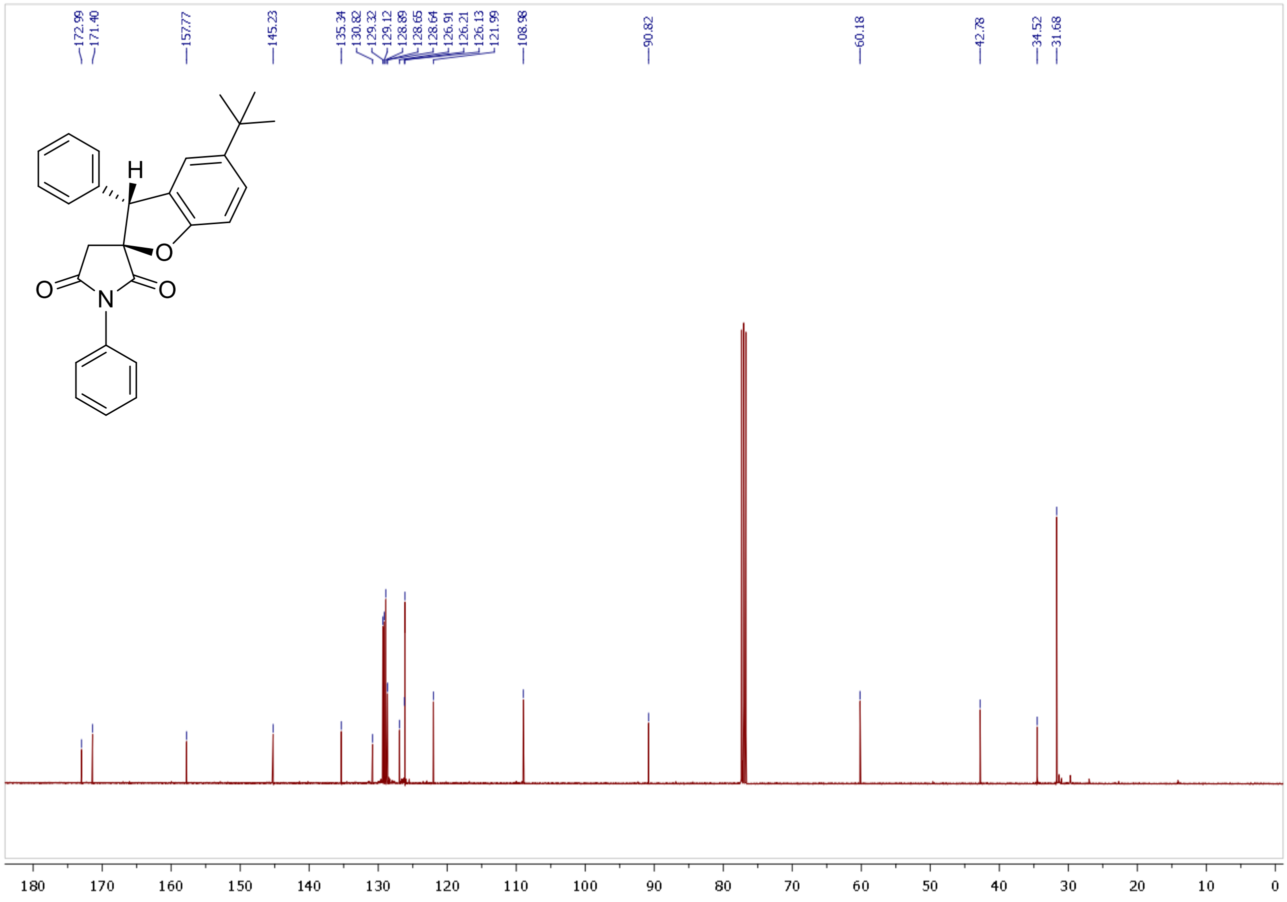

Copies of ^{1}H (400.13 MHz, $CDCl_3$) and ^{13}C (100.61 MHz, $CDCl_3$) spectra of *syn*-**7b**

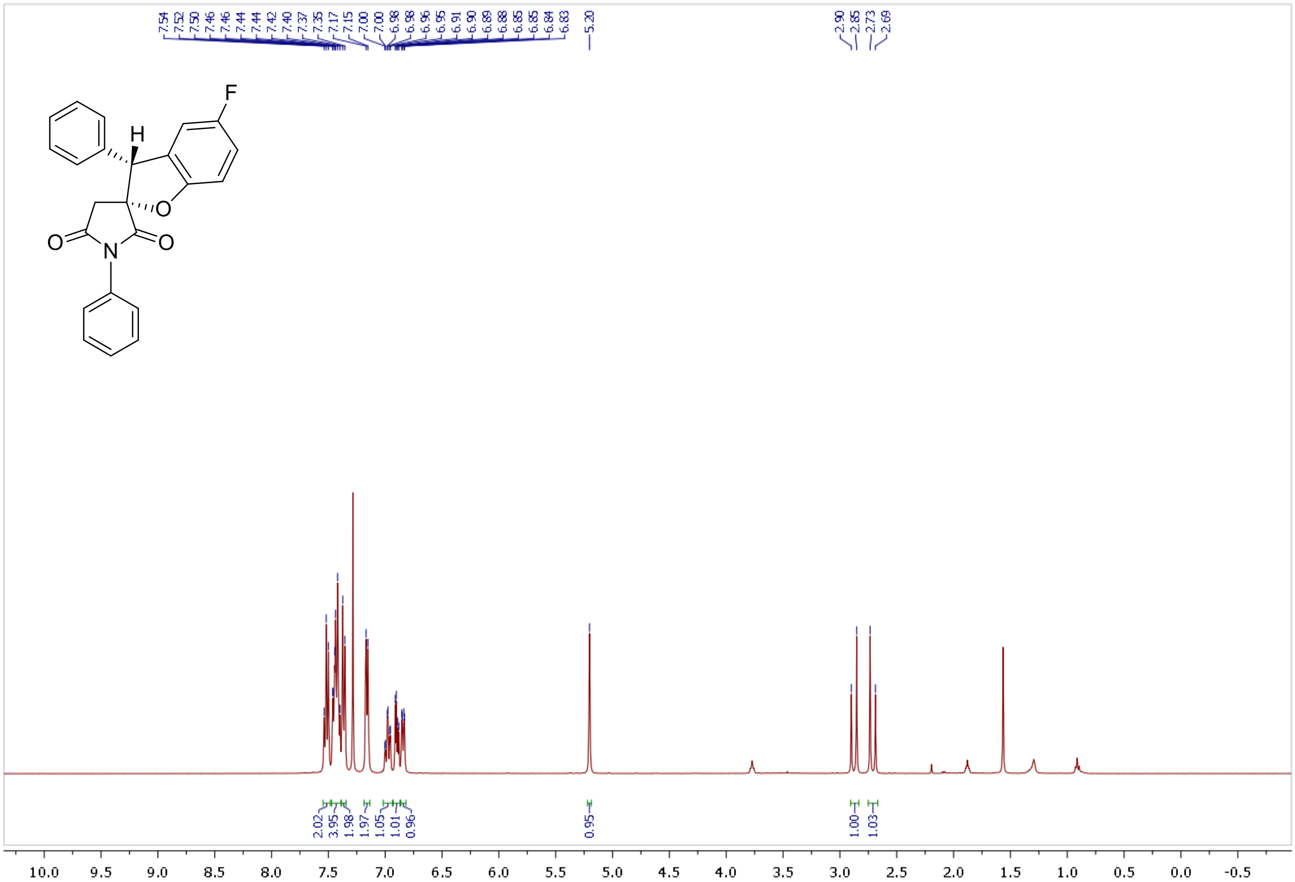

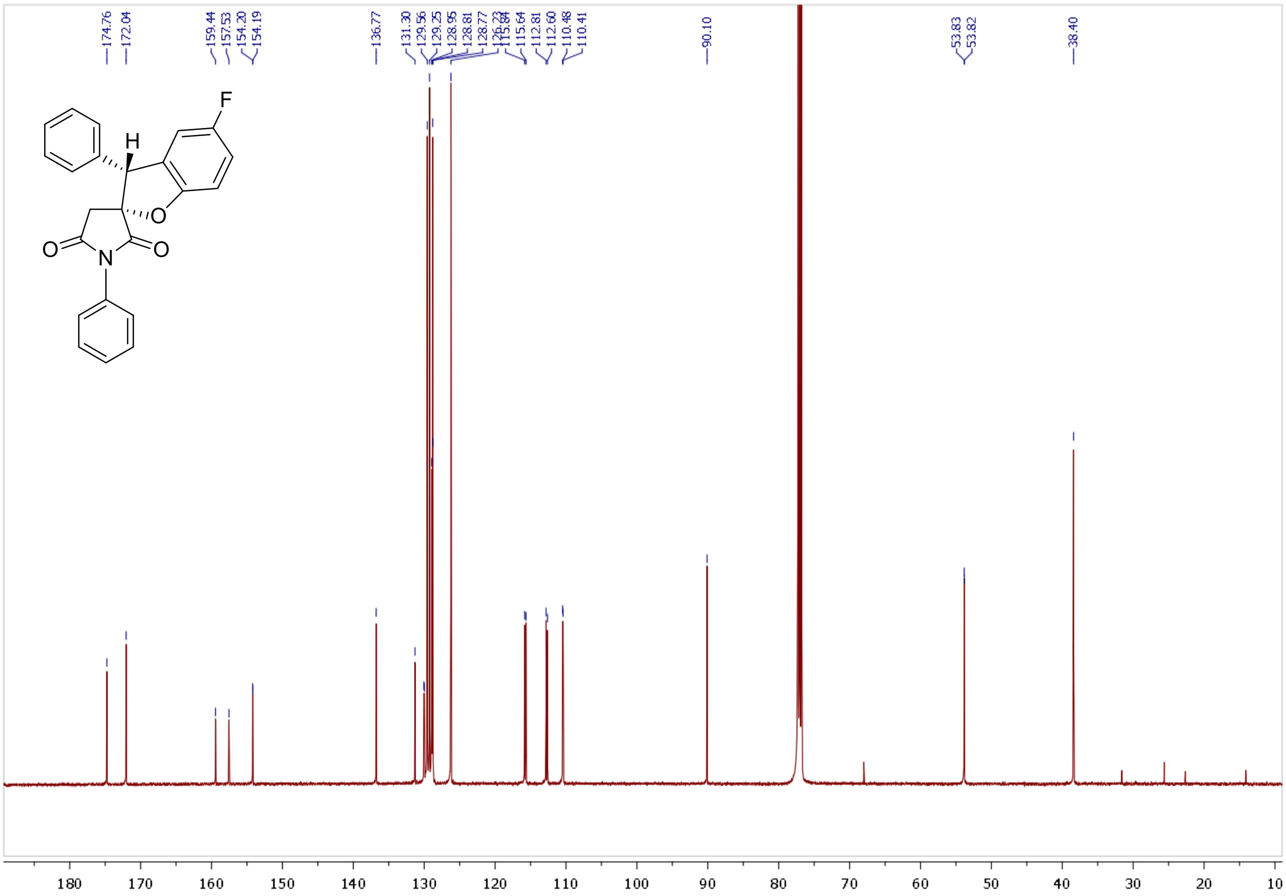

Copy of ^{19}F (376.50 MHz, $CDCl_3$) spectrum of *syn*-**7b**

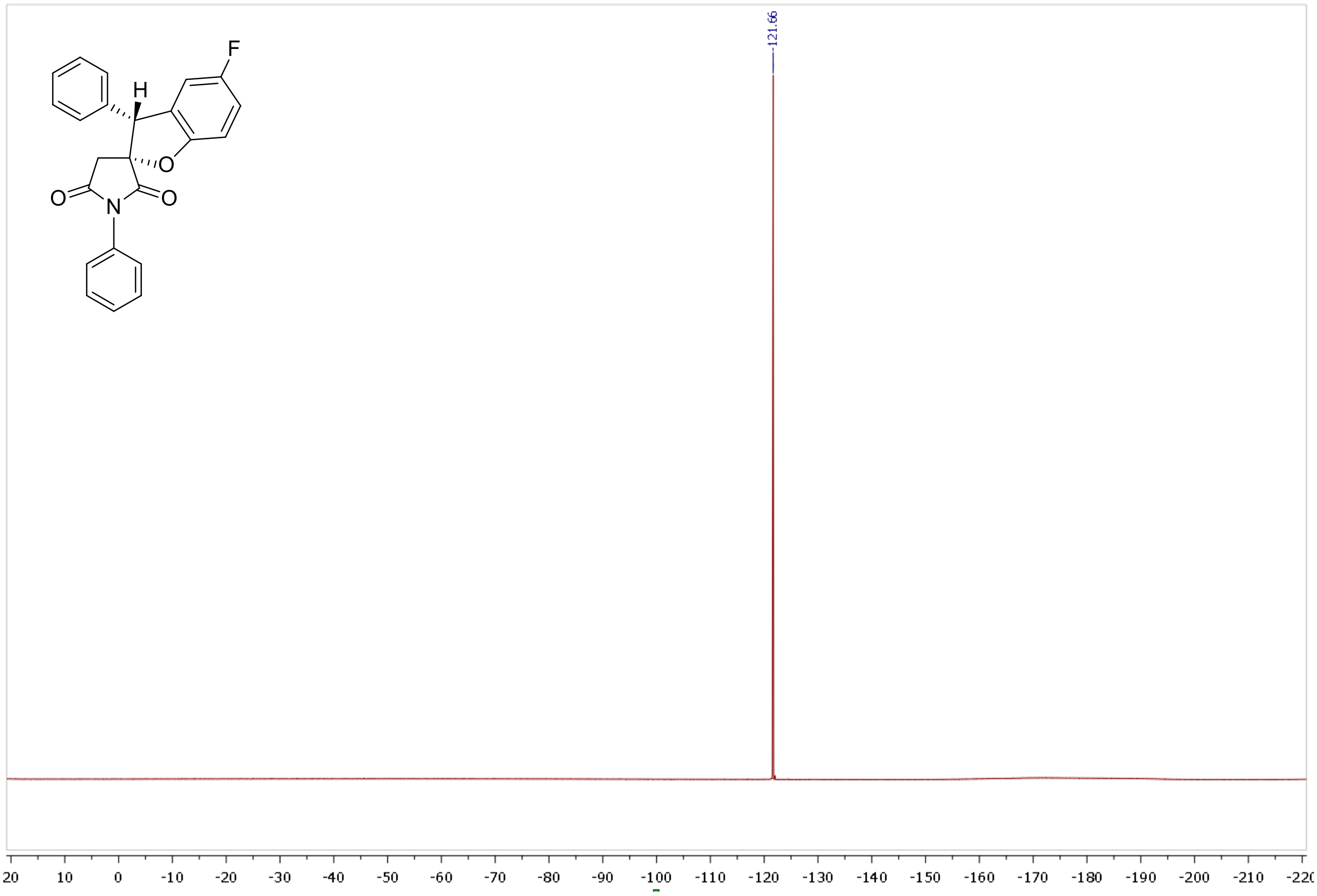

Copies of ^{1}H (400.13 MHz, $CDCl_3$) and ^{13}C (100.61 MHz, $CDCl_3$) spectra of *syn*-**7c**

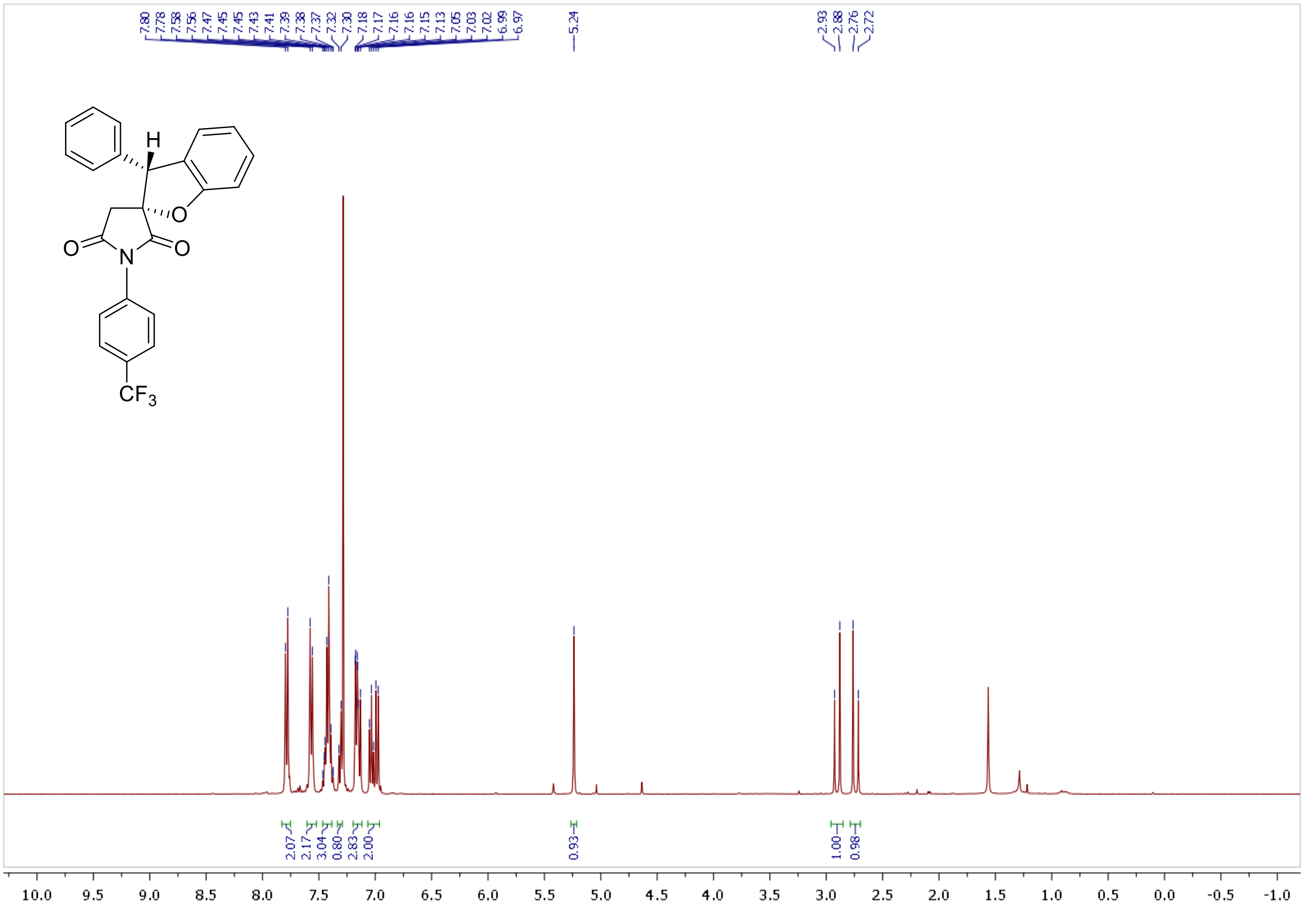

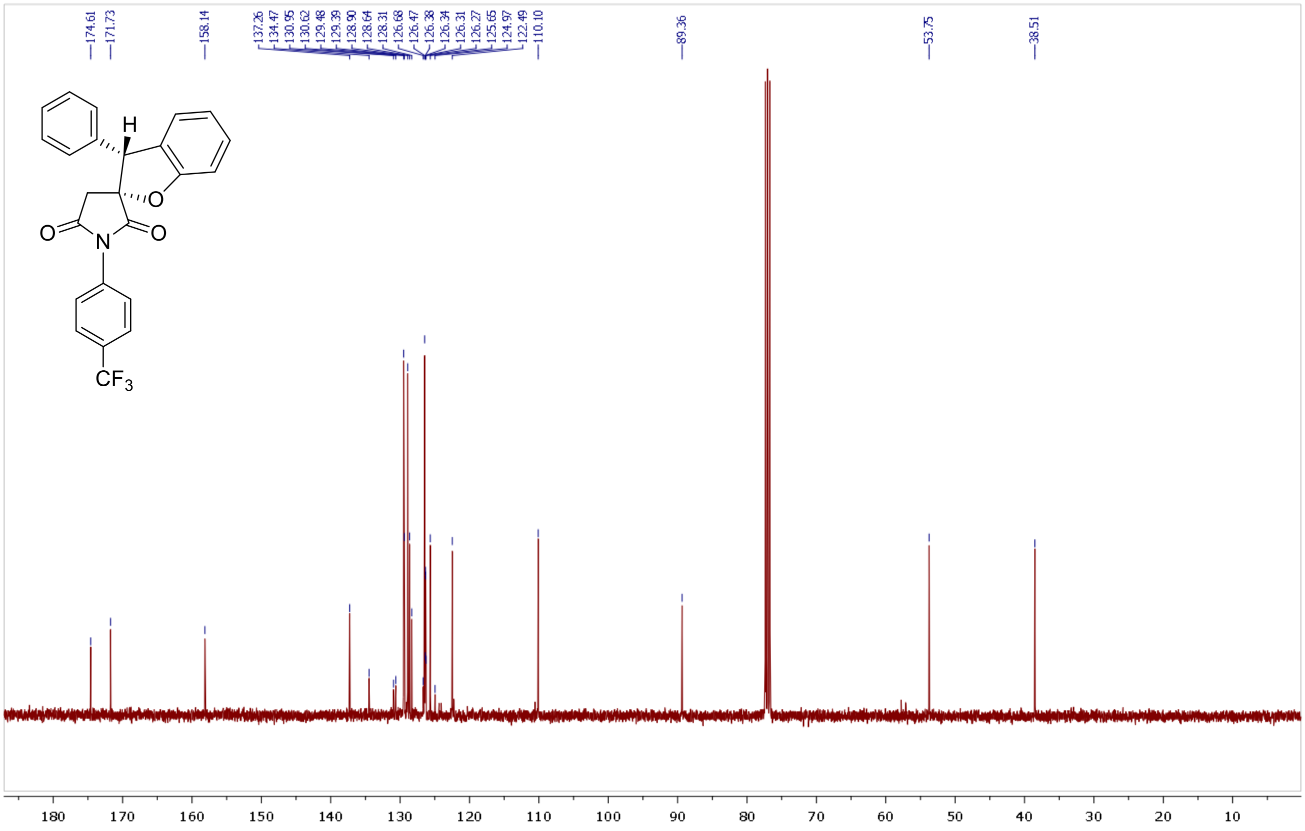

Copy of ^{19}F (376.50 MHz, $CDCl_3$) spectrum of *syn*-**7b**

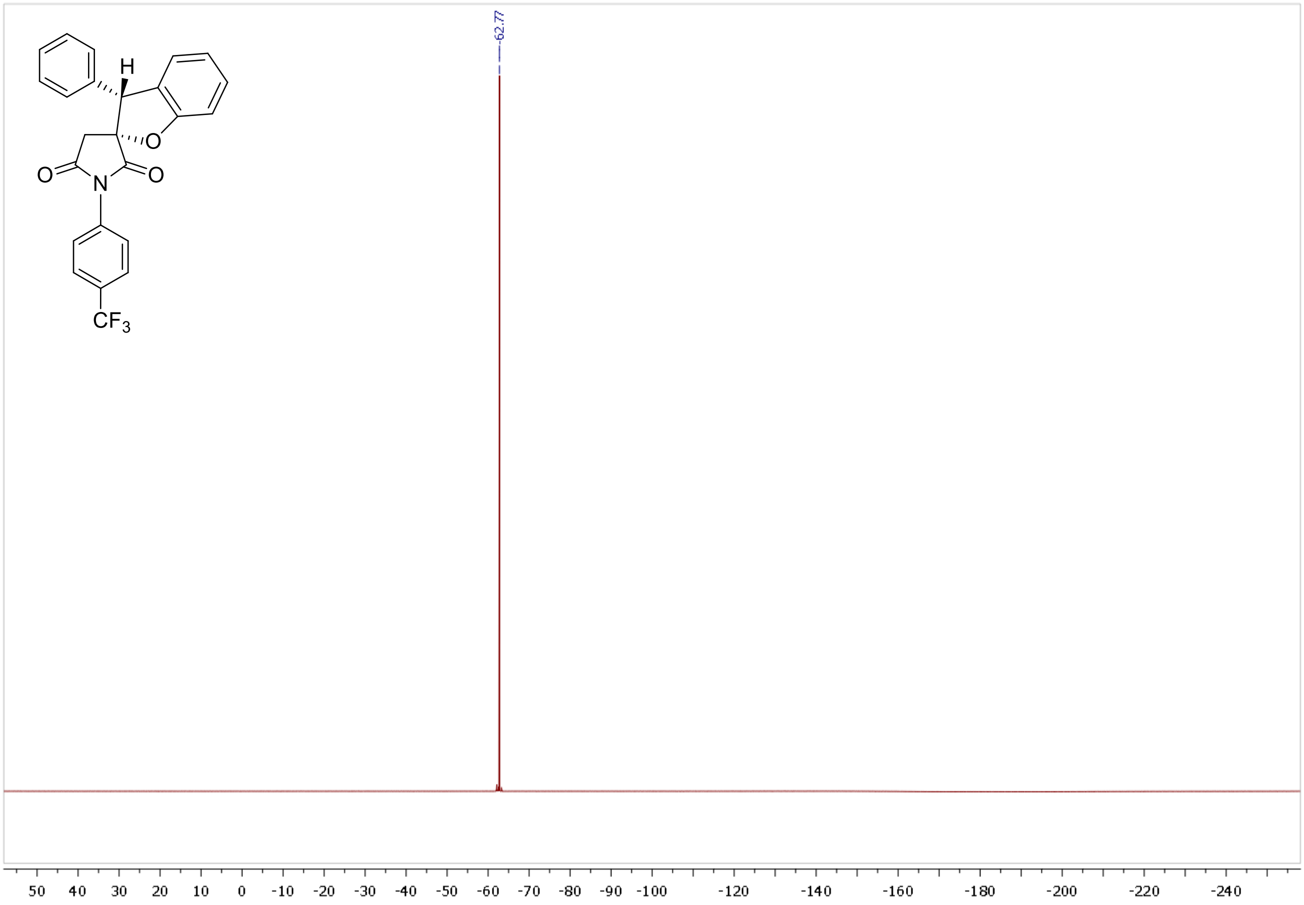

Copies of ^{1}H (400.13 MHz, $CDCl_3$) and ^{13}C (100.61 MHz, $CDCl_3$) spectra of *anti*-**7c**

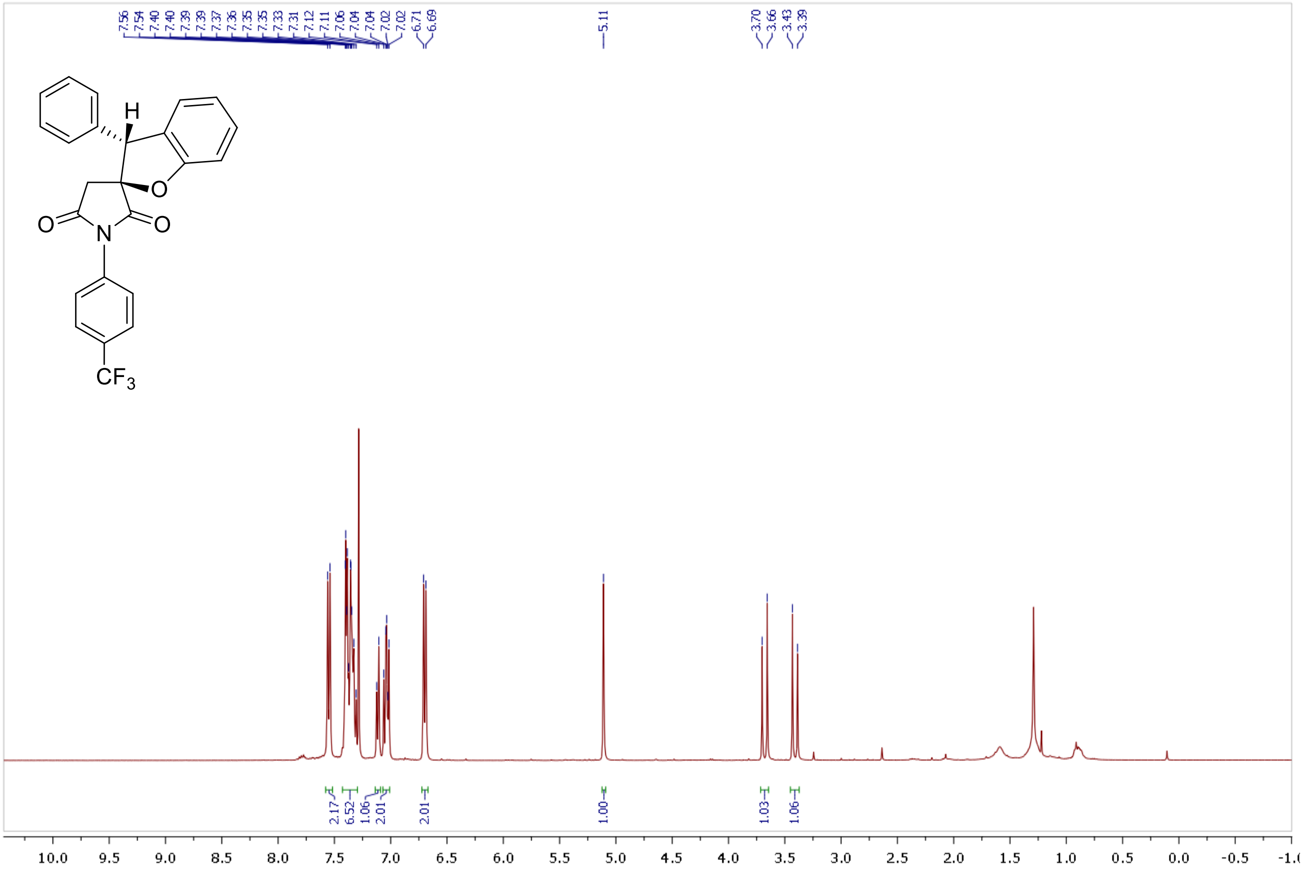

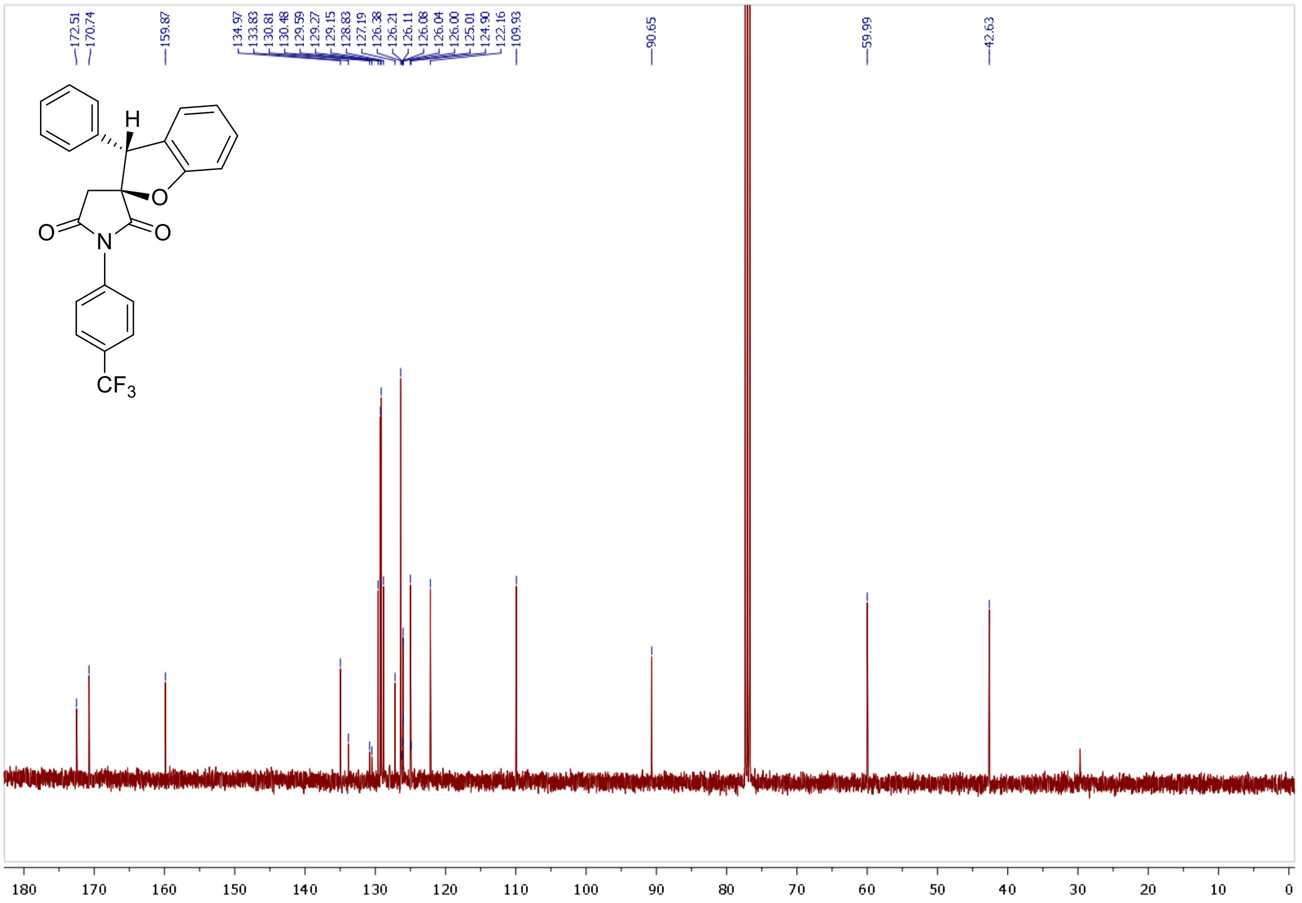

Copy of ^{19}F (376.50 MHz, $CDCl_3$) spectrum of *anti*-**7c**

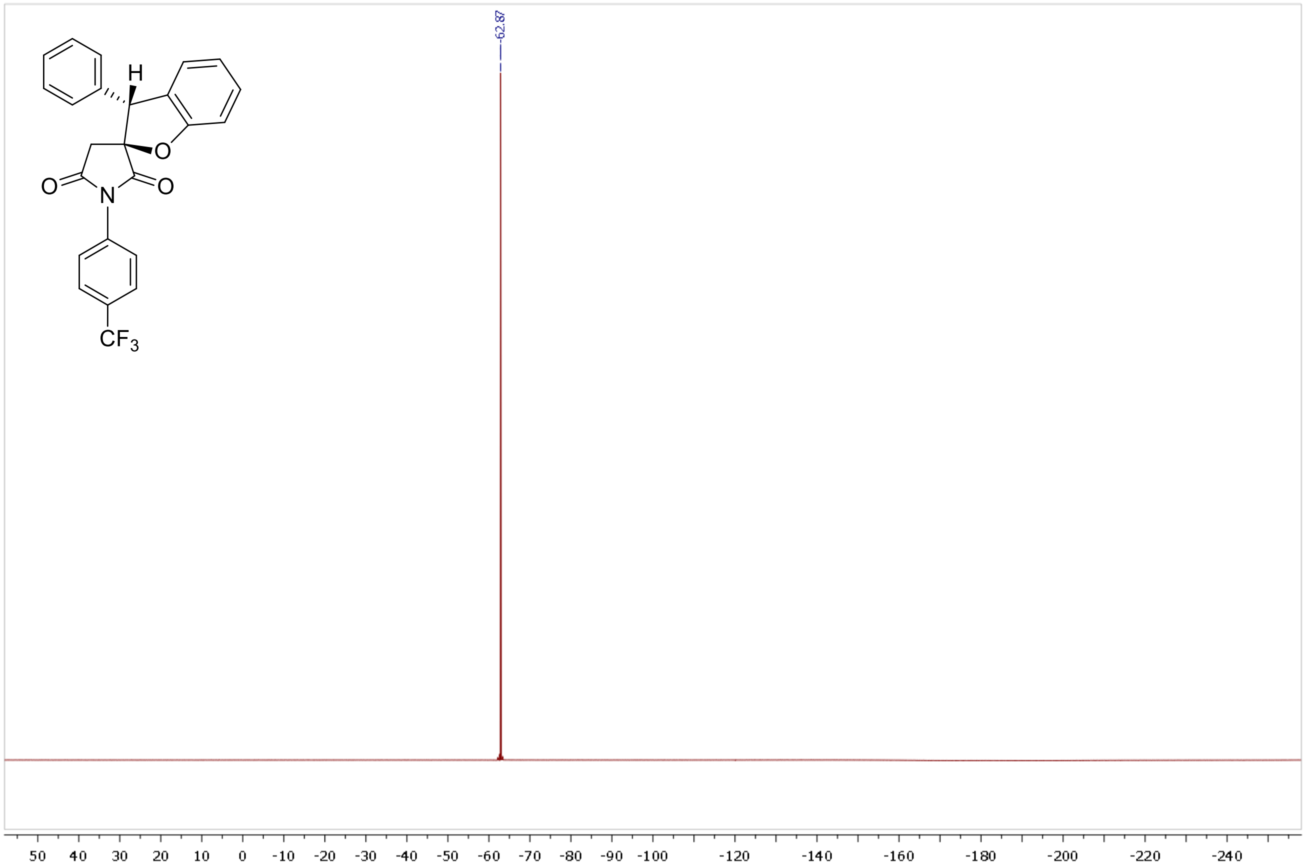

Copies of ^{1}H (400.13 MHz, $CDCl_3$) and ^{13}C (100.61 MHz, $CDCl_3$) spectra of *syn*-**7d**

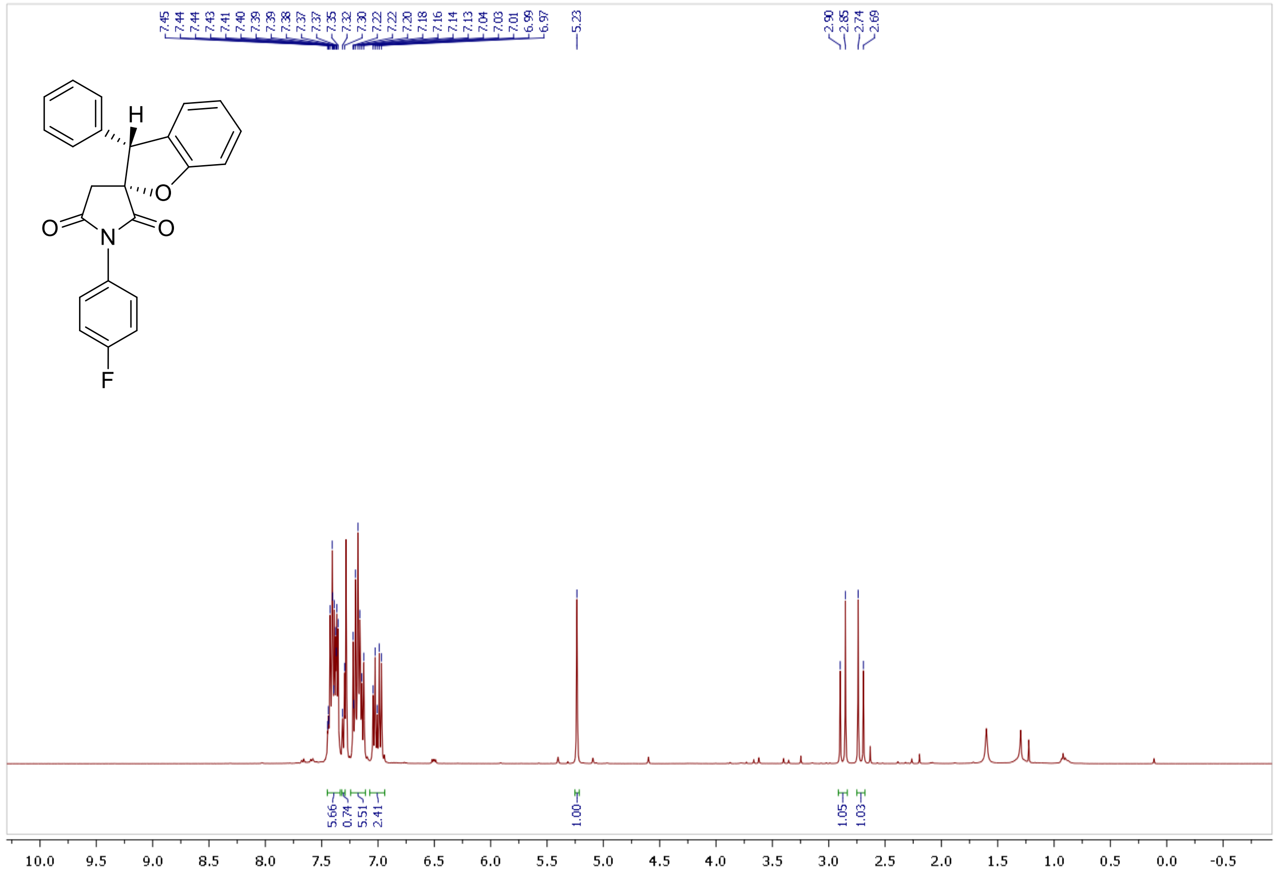

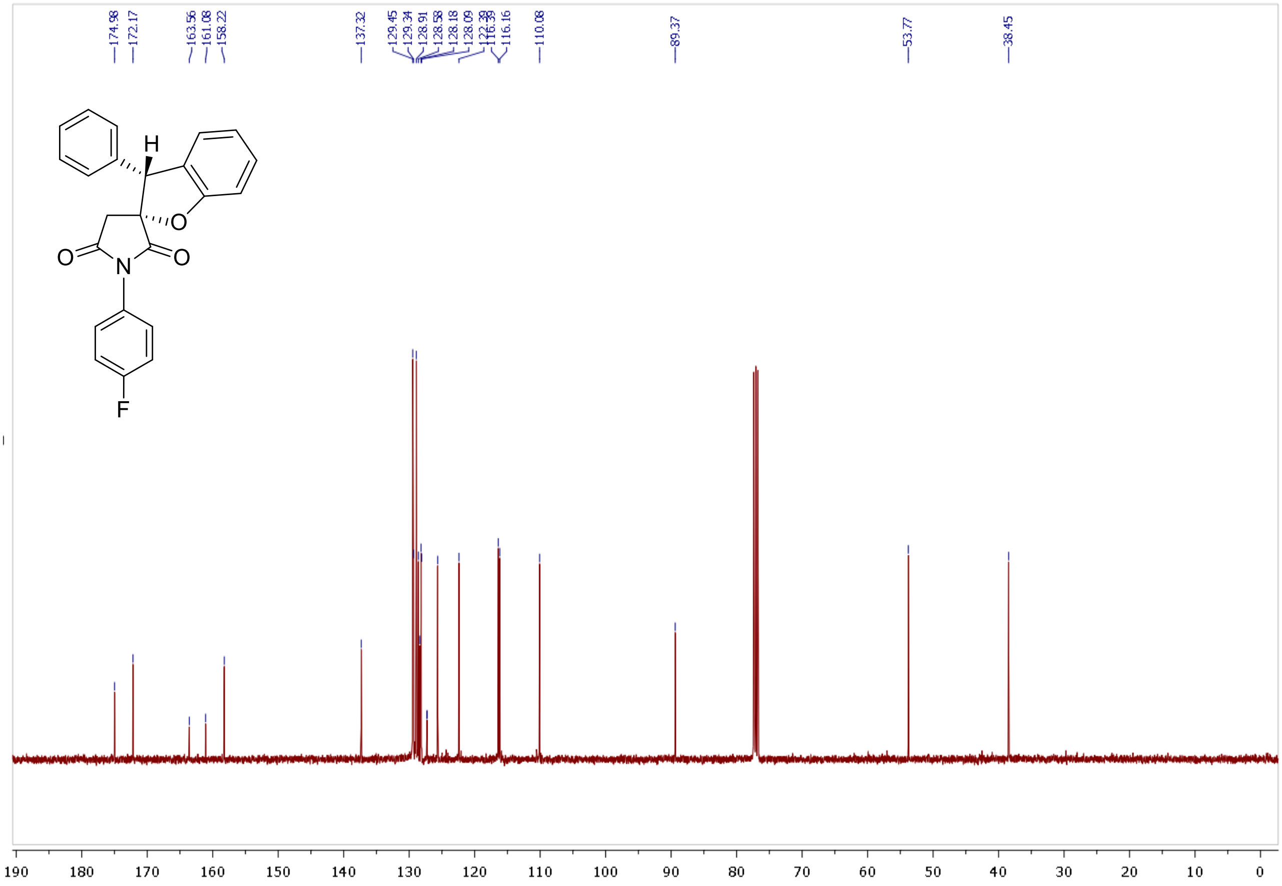

Copy of ^{19}F (376.50 MHz, $CDCl_3$) spectrum of *syn*-**7d**

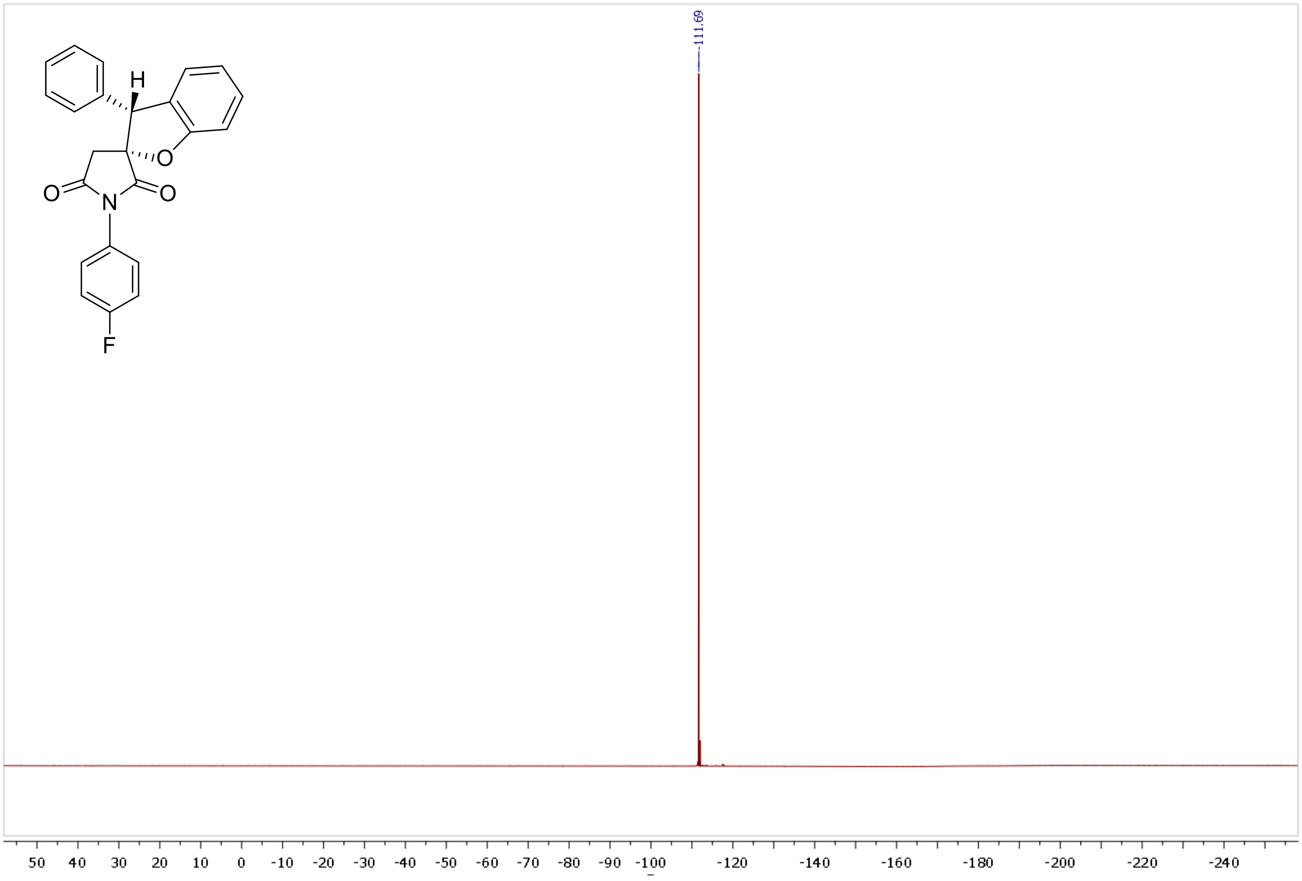

Copies of ^{1}H (400.13 MHz, $CDCl_3$) and ^{13}C (100.61 MHz, $CDCl_3$) spectra of *syn*-**7e**

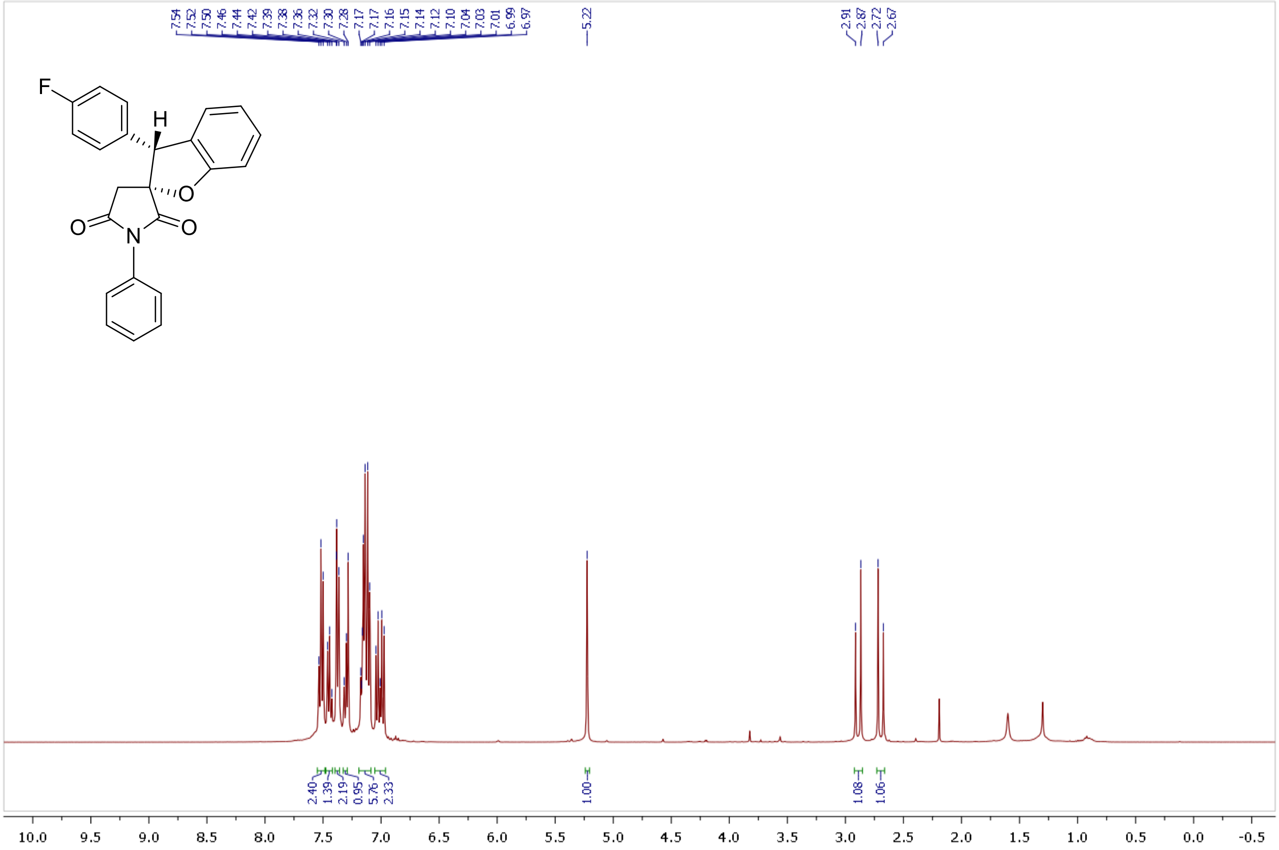

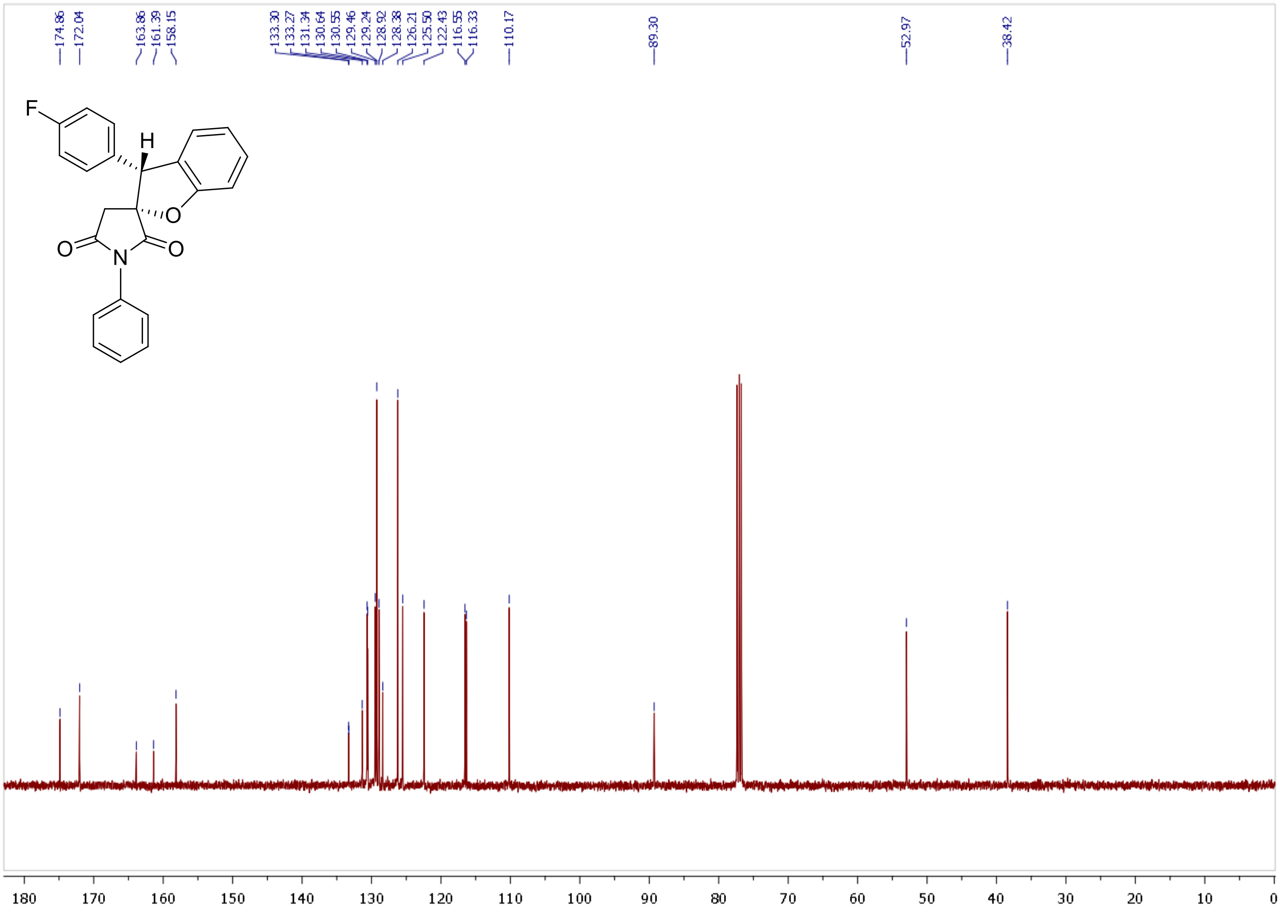

Copy of ^{19}F (376.50 MHz, $CDCl_3$) spectrum of *syn*-**7e**

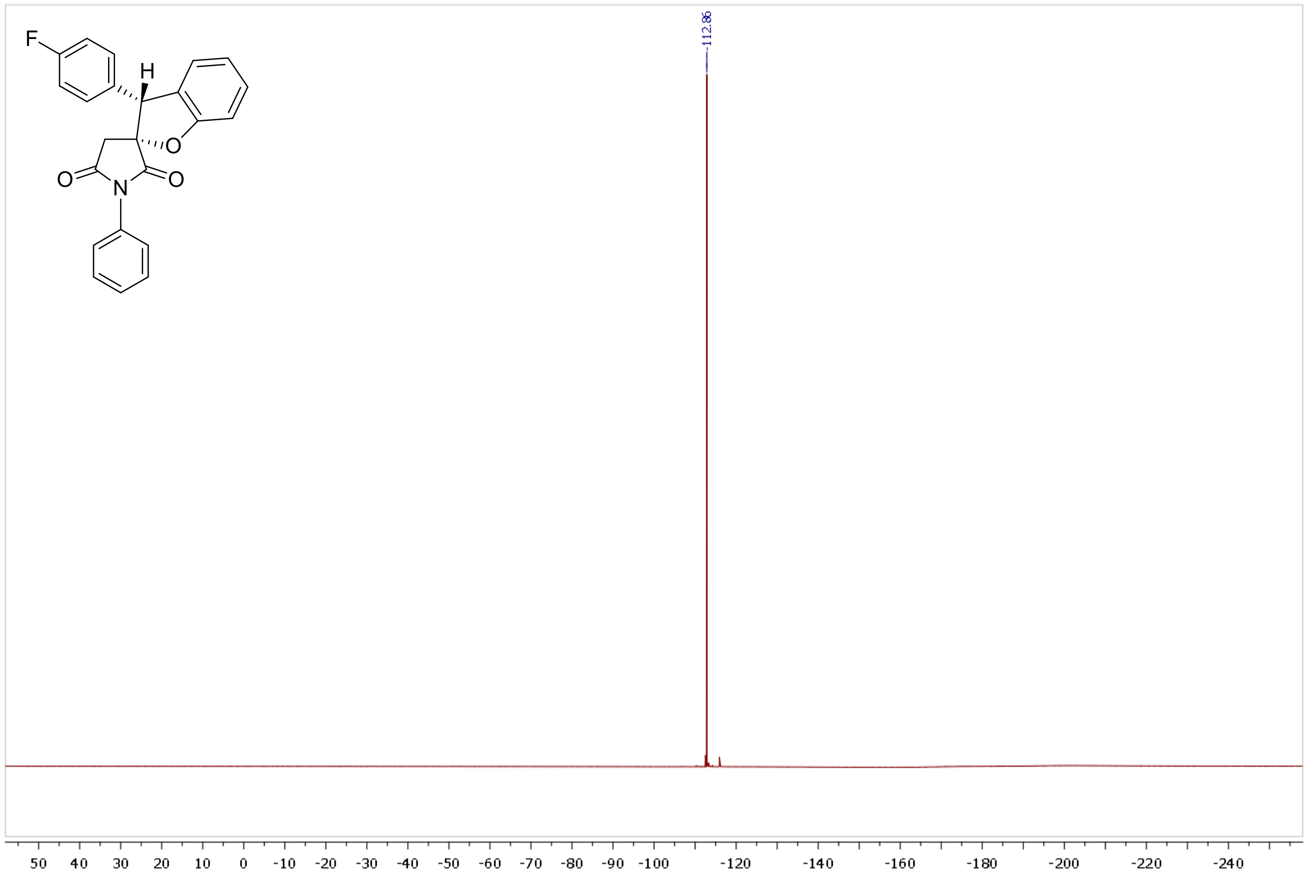

Copies of ^{1}H (400.13 MHz, $CDCl_3$) and ^{13}C (100.61 MHz, $CDCl_3$) spectra of *syn*-**7f**

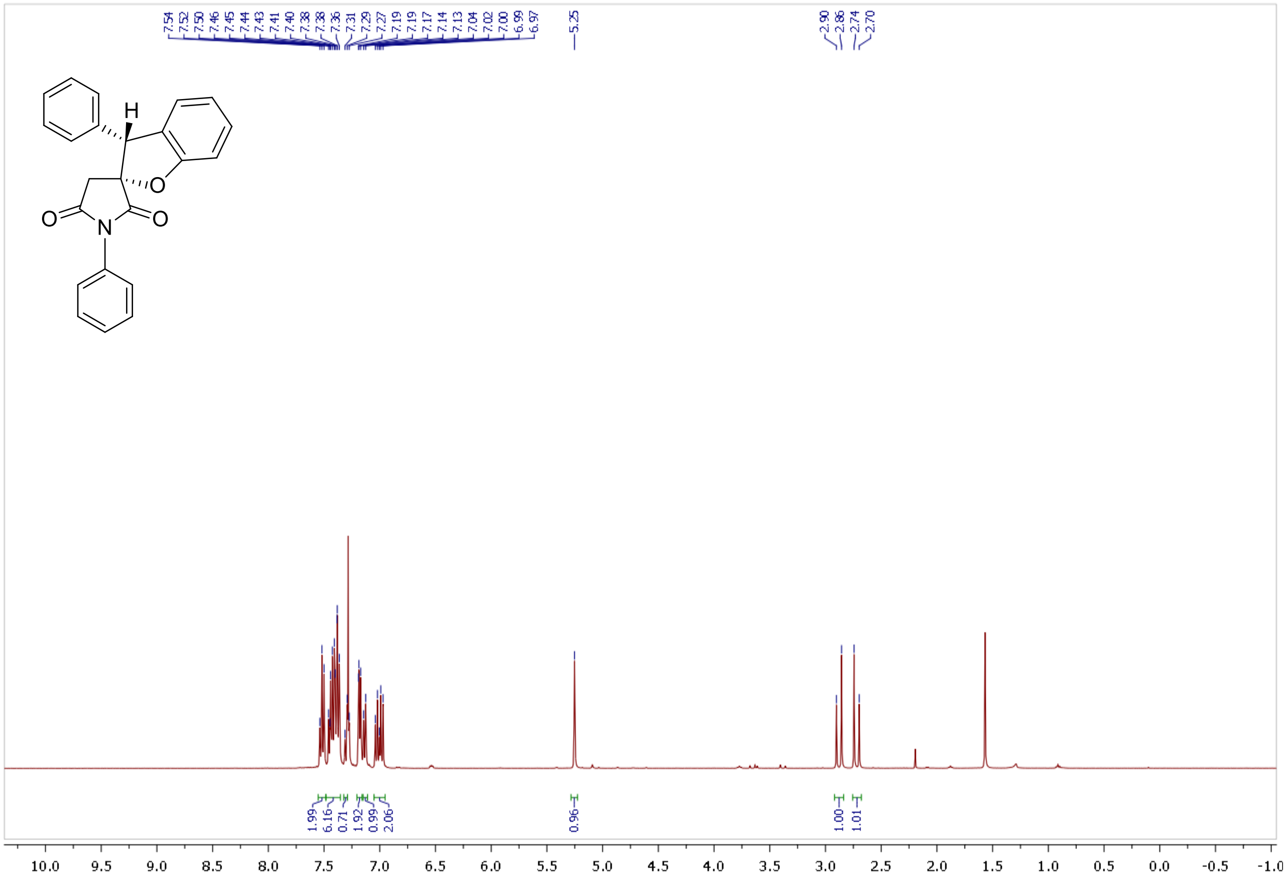

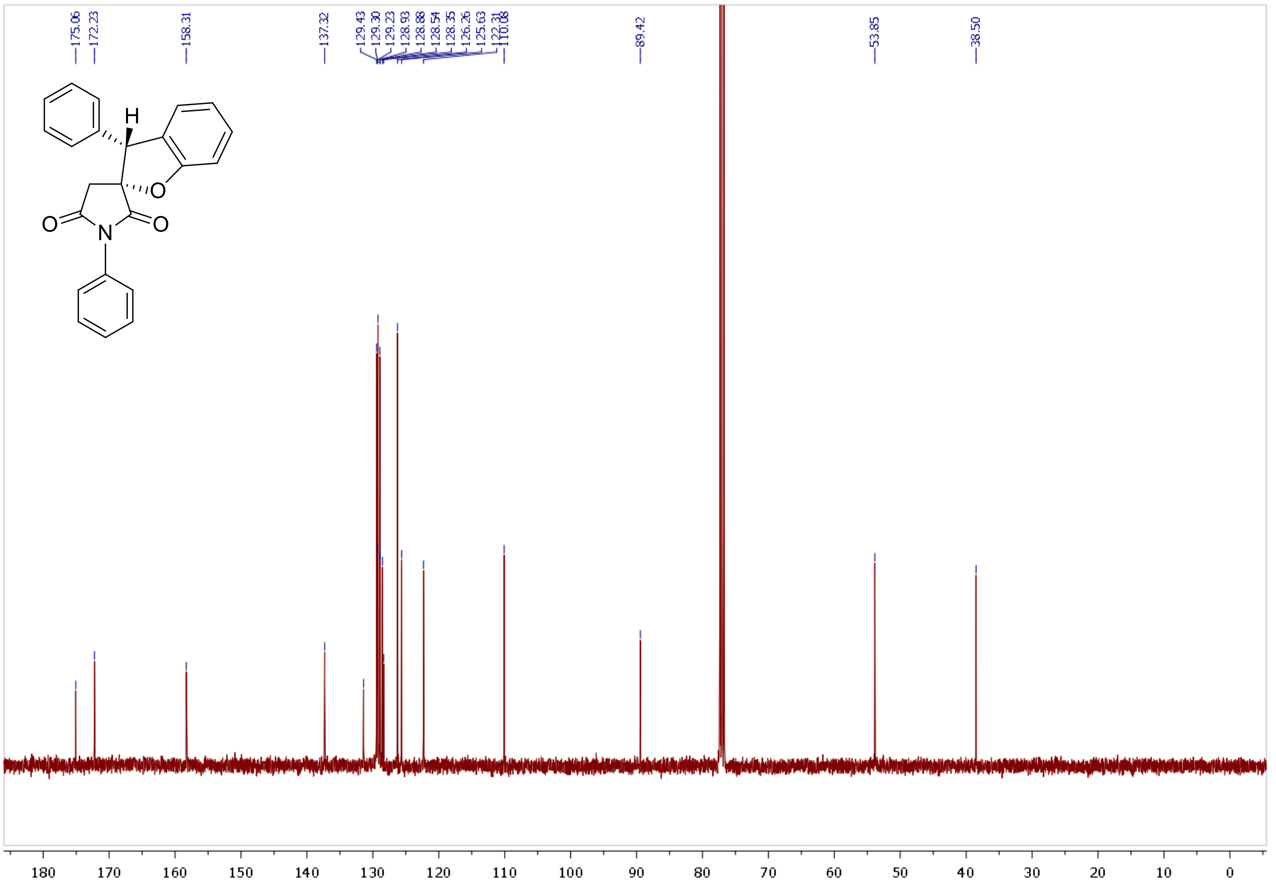

Copies of ^{1}H (400.13 MHz, $CDCl_3$) and ^{13}C (100.61 MHz, $CDCl_3$) spectra of *syn*-**7g**

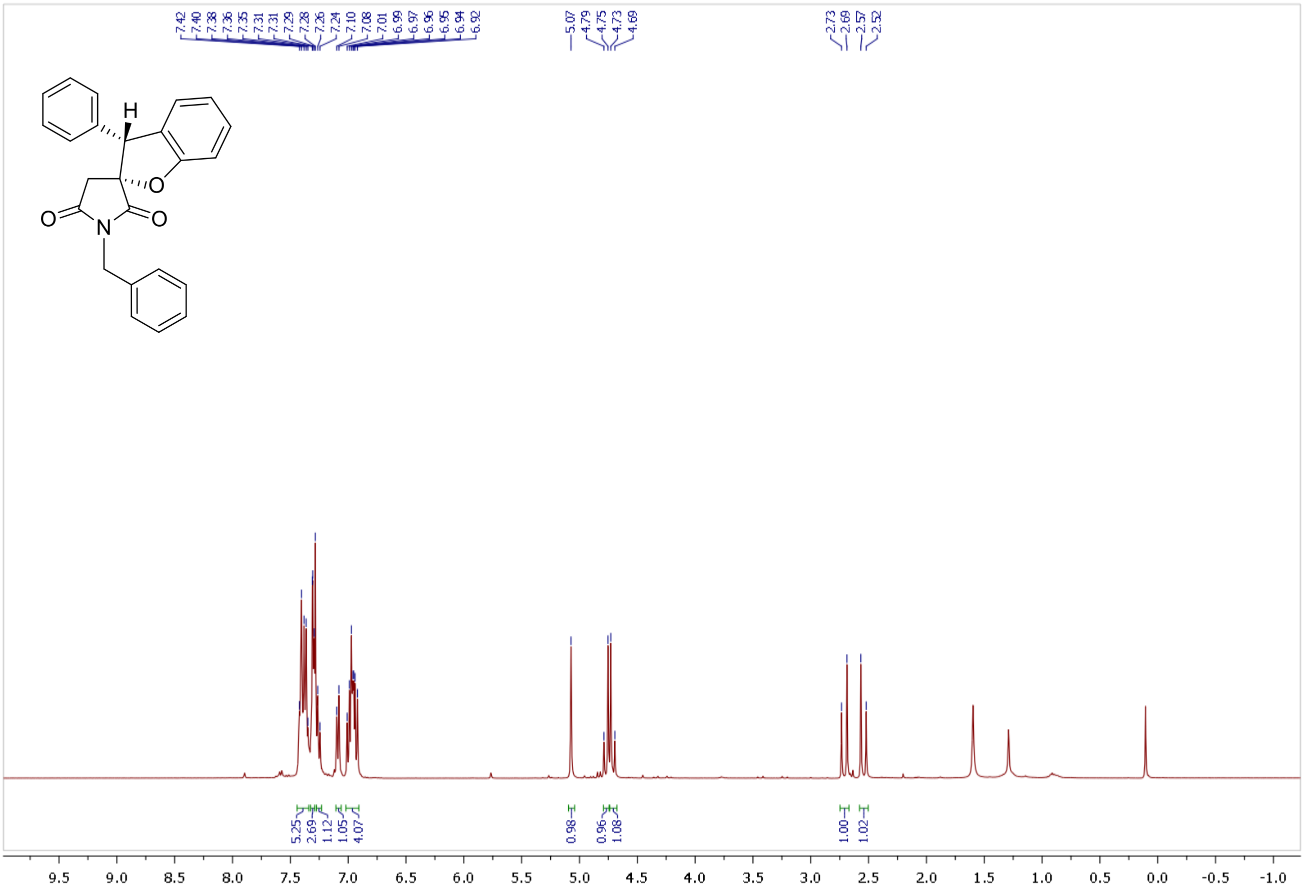

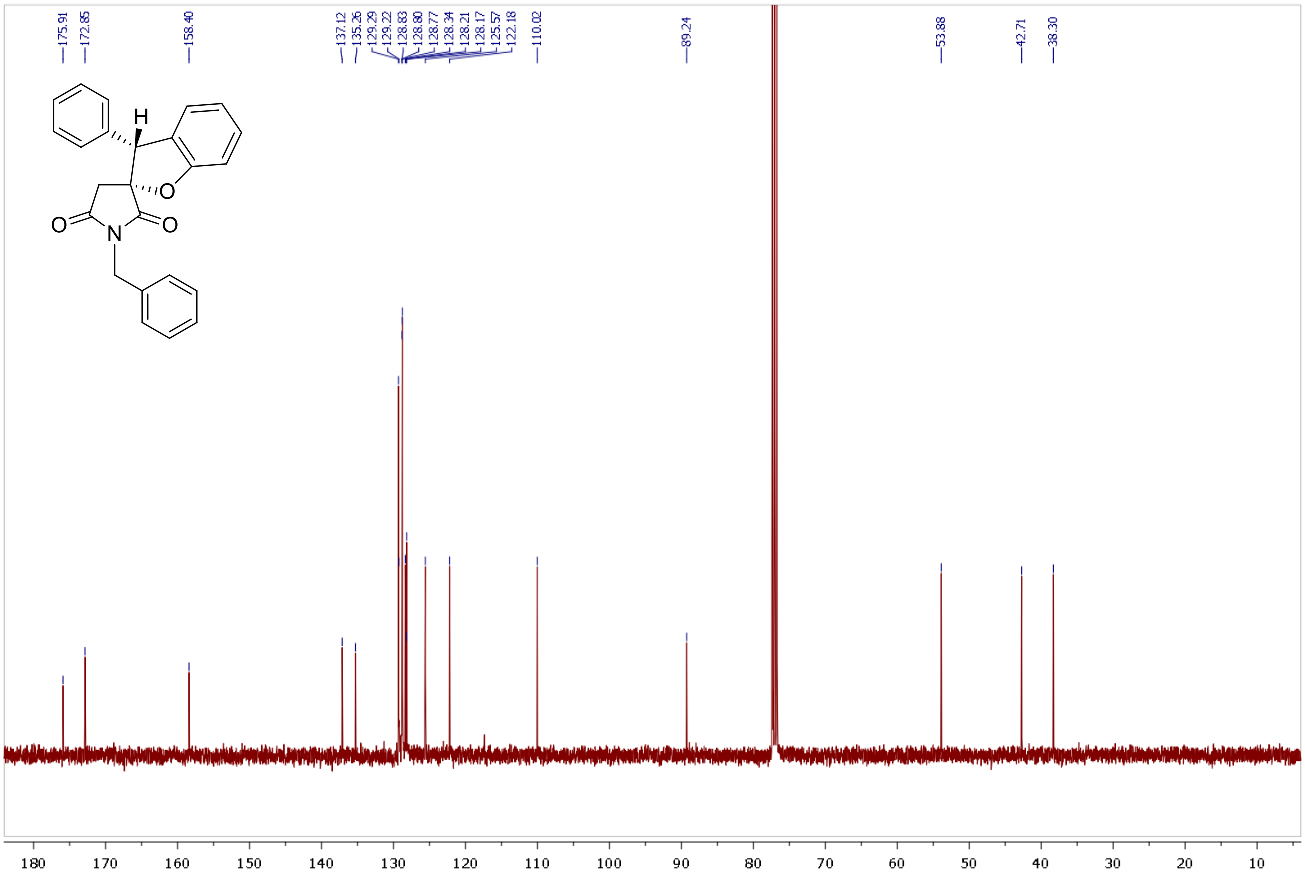

Copies of ^{1}H (400.13 MHz, $CDCl_3$) and ^{13}C (100.61 MHz, $CDCl_3$) spectra of *syn*-**7h**

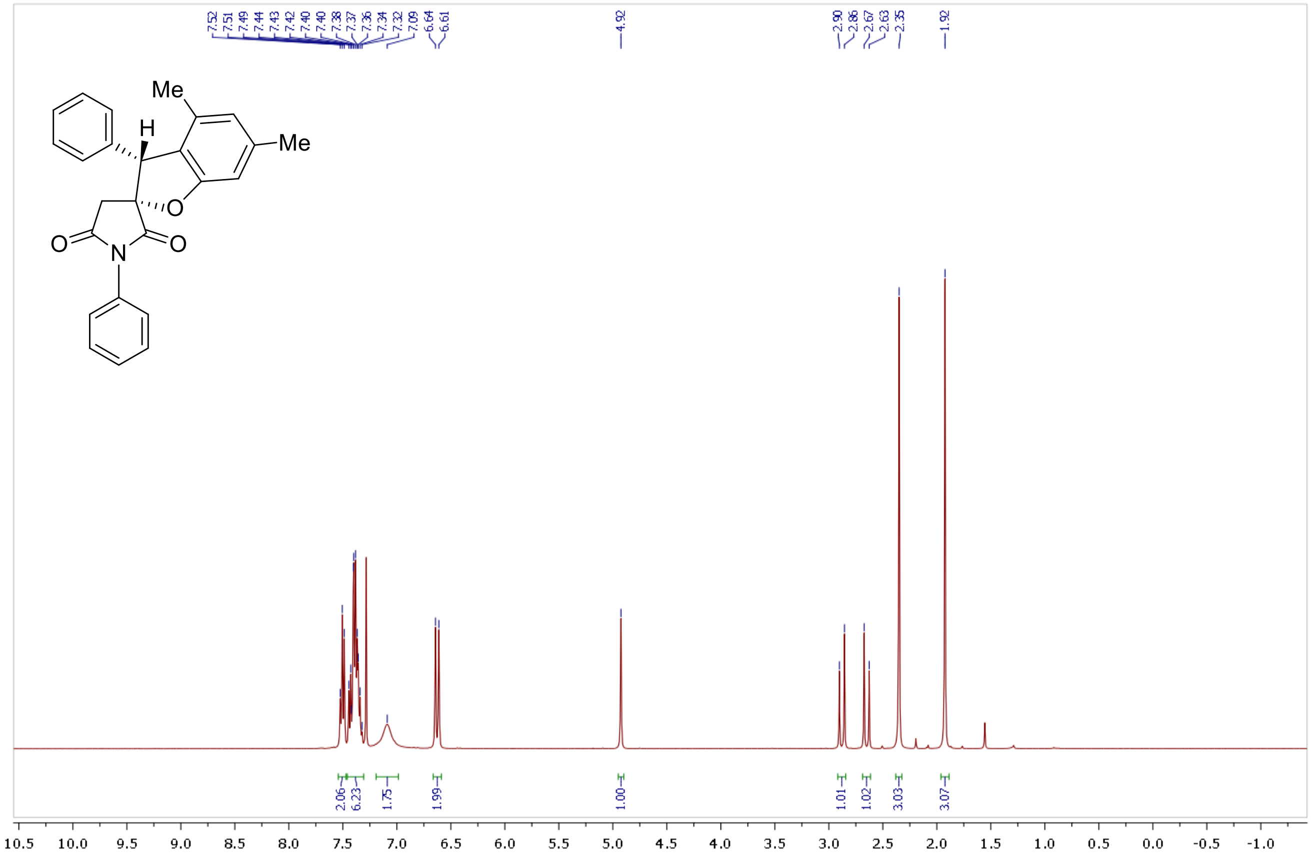

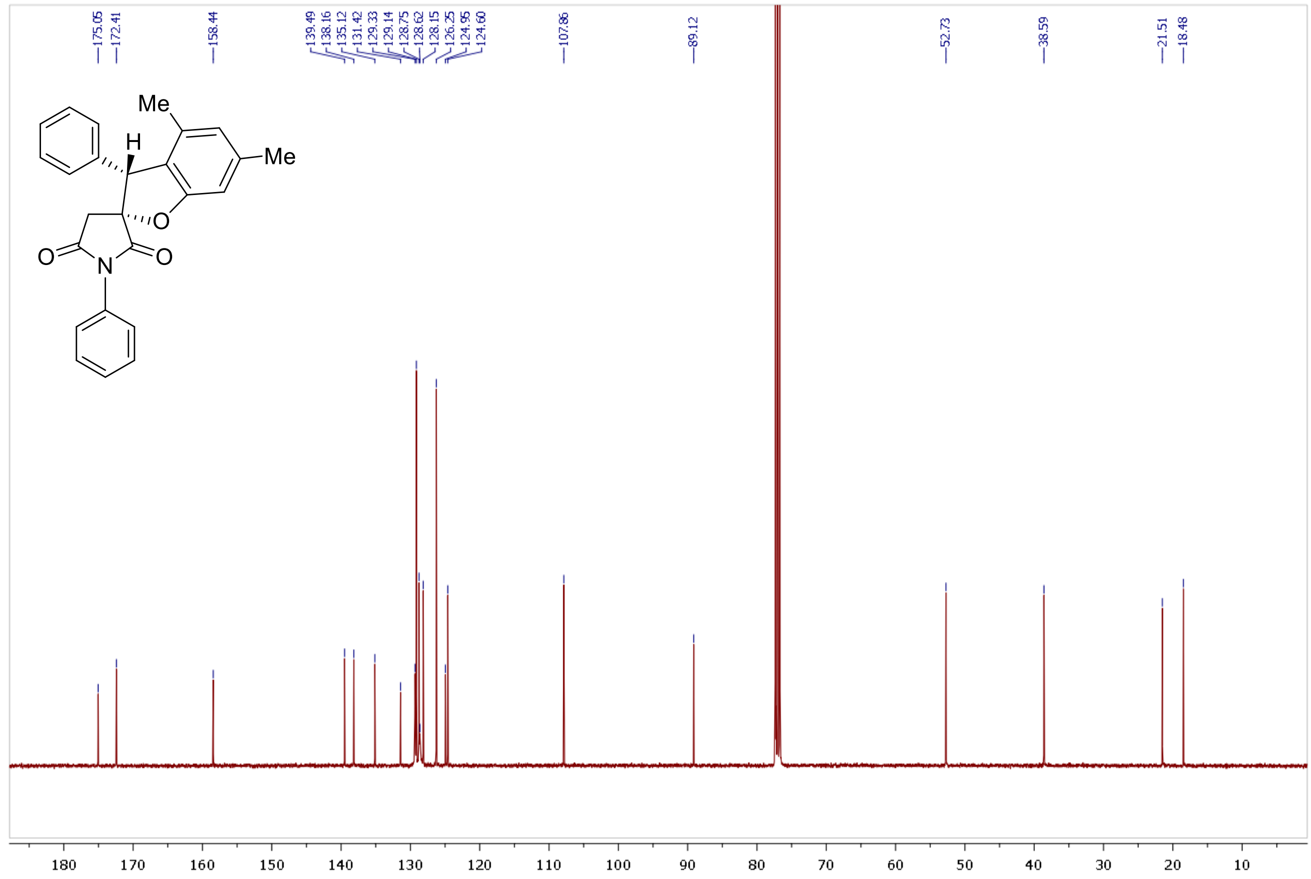

Copies of ^{1}H (400.13 MHz, $CDCl_3$) and ^{13}C (100.61 MHz, $CDCl_3$) spectra of *syn*-**7i**

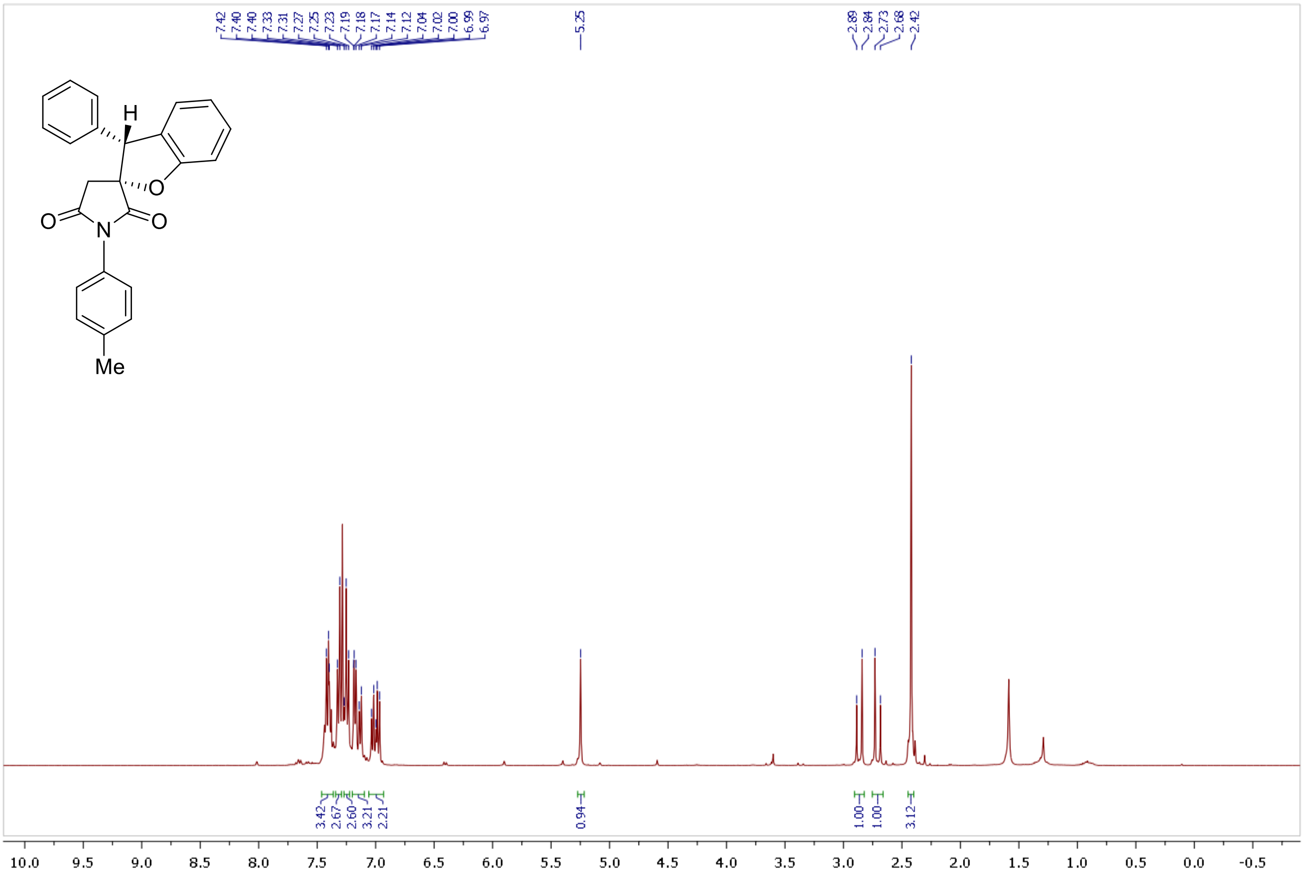

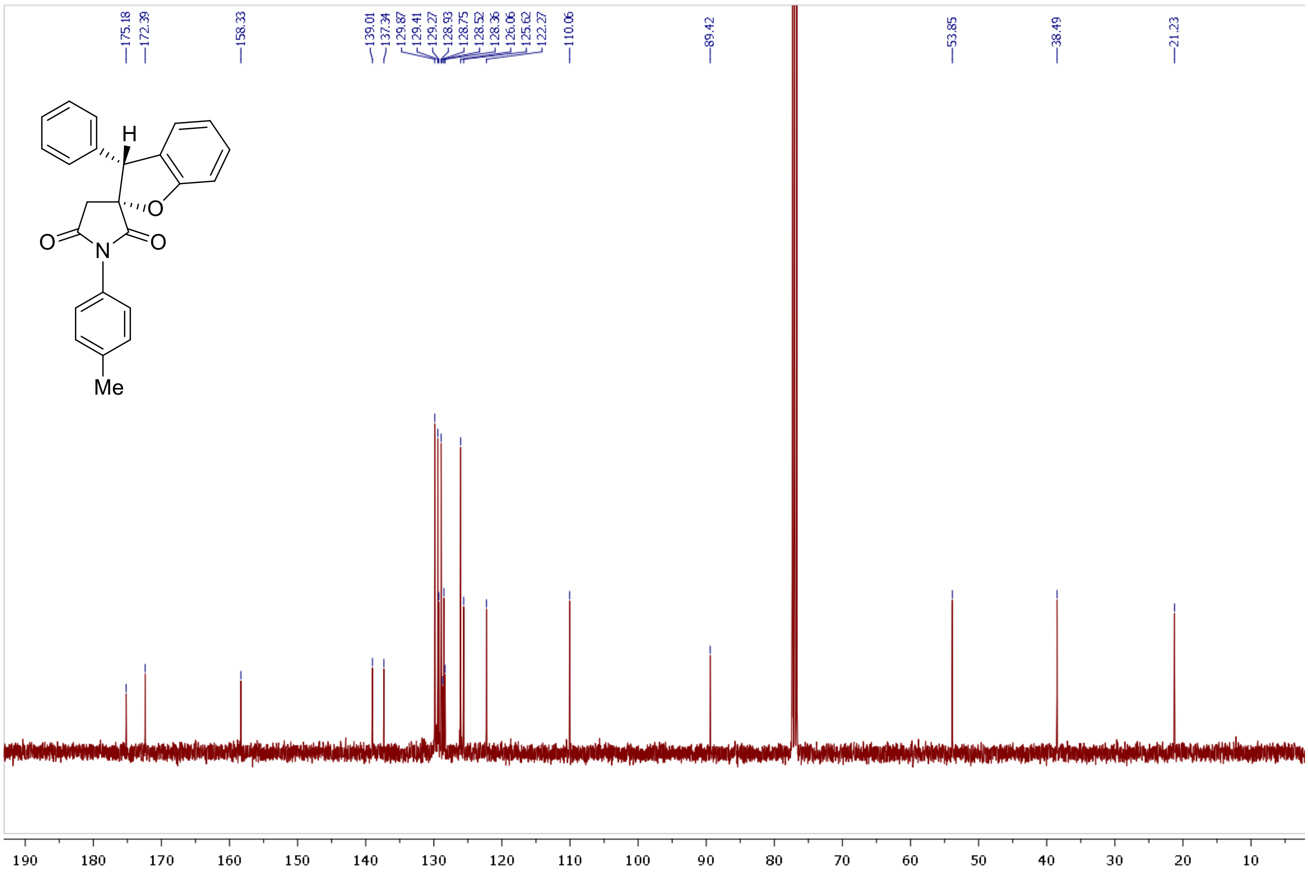

Copies of ^1H (400.13 MHz, $CDCl_3$) and ^{13}C (100.61 MHz, $CDCl_3$) spectra of *syn*-**7j**

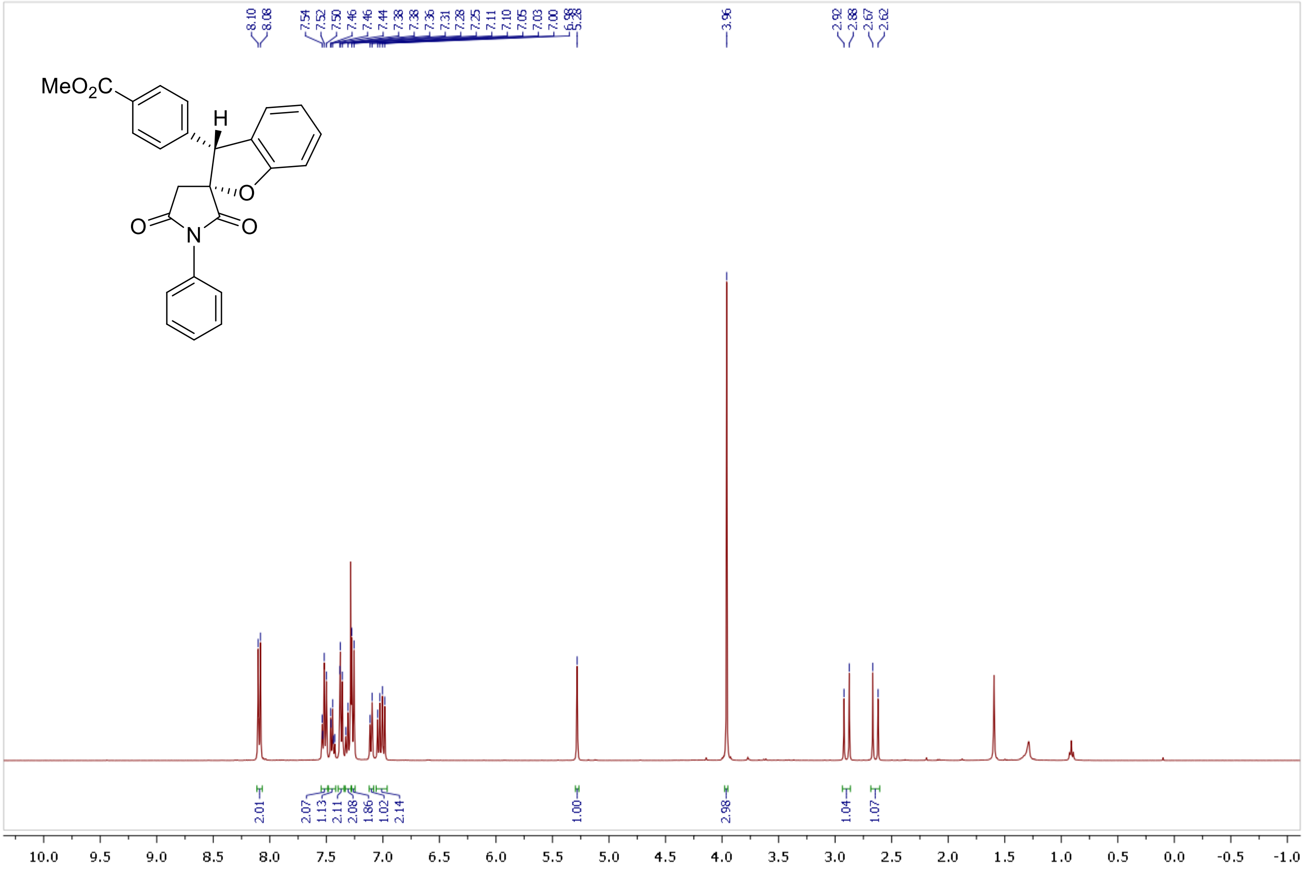

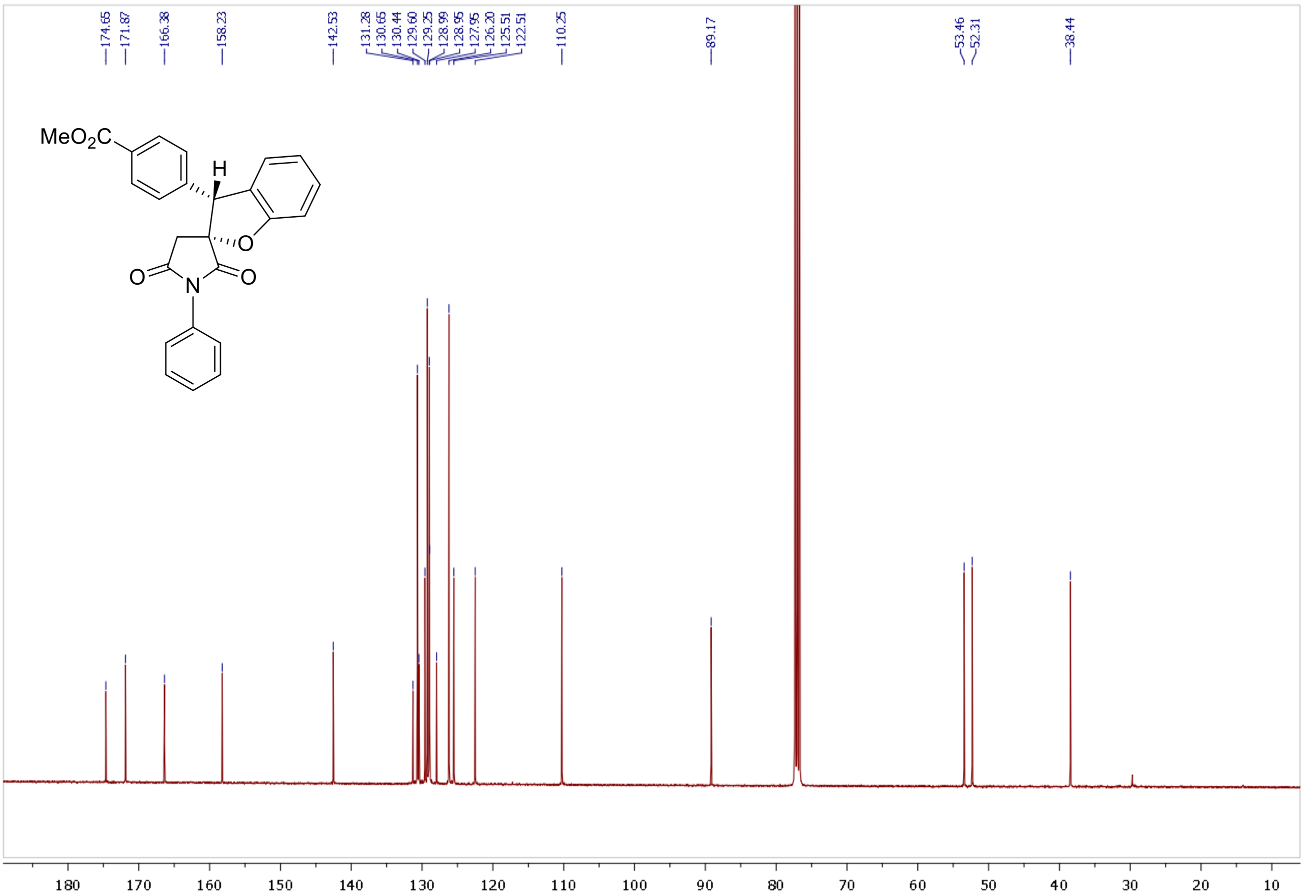

Copies of ^{1}H (400.13 MHz, $CDCl_3$) and ^{13}C (100.61 MHz, $CDCl_3$) spectra of *syn*-**7k**

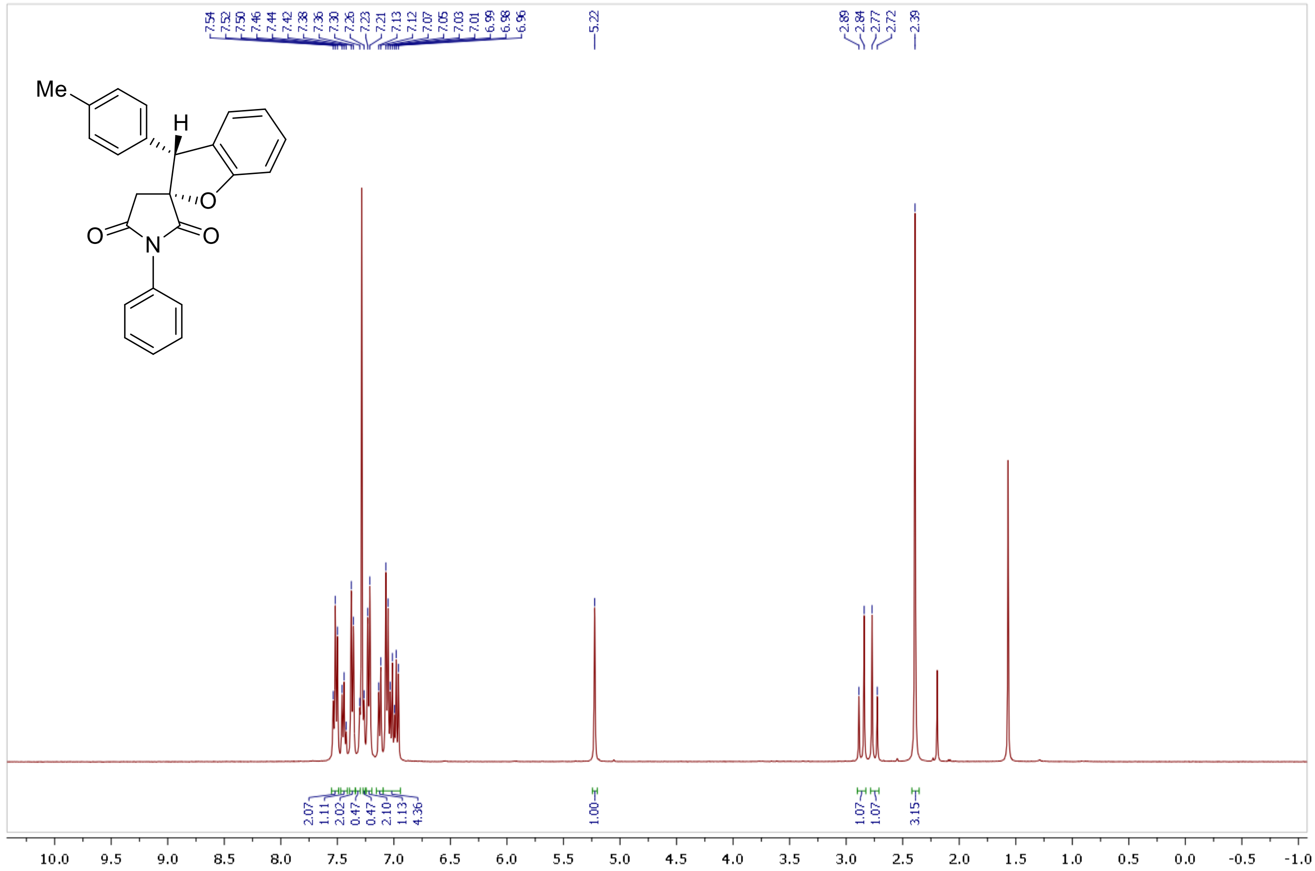

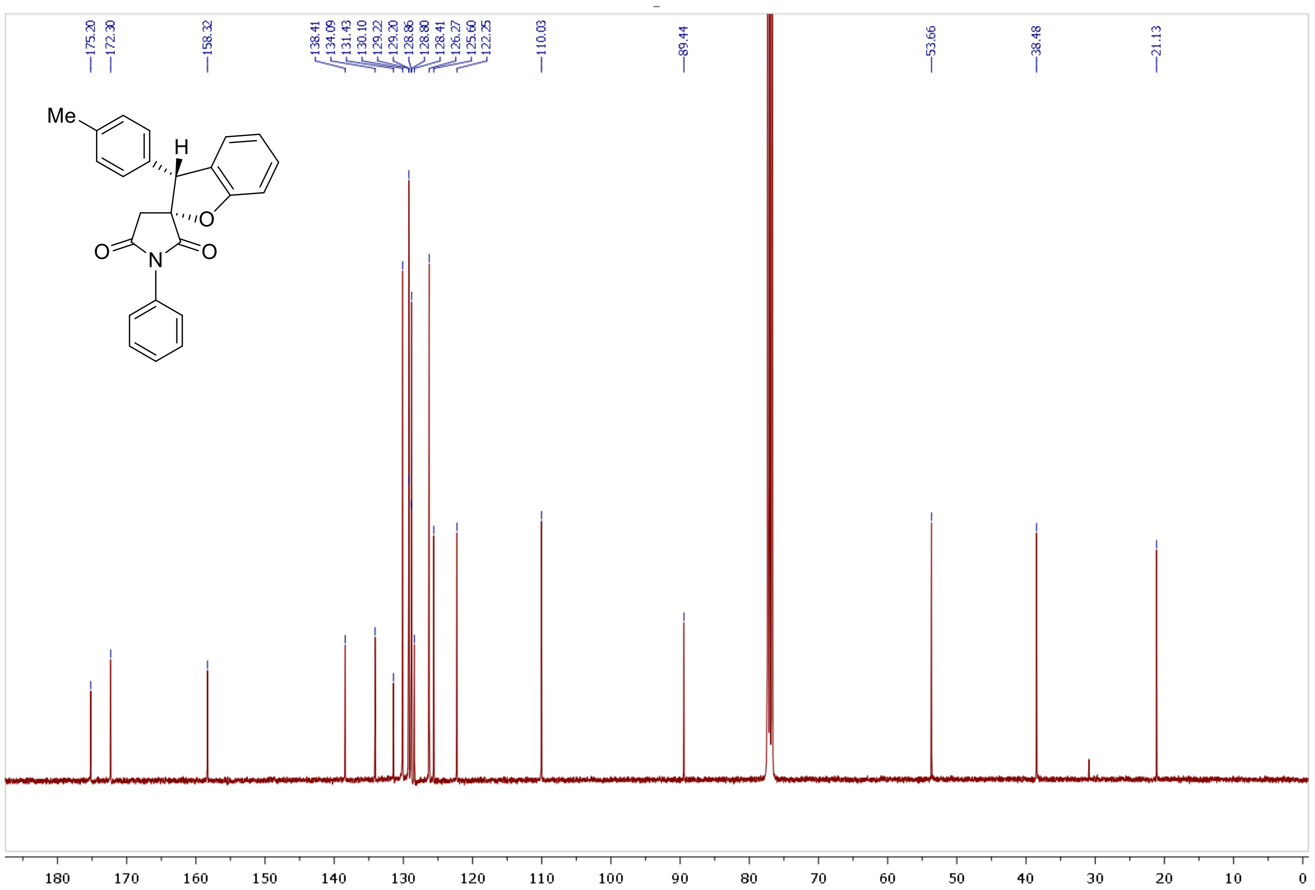

Copies of ^{1}H (400.13 MHz, $CDCl_3$) and ^{13}C (100.61 MHz, $CDCl_3$) spectra of *syn*-**7l**

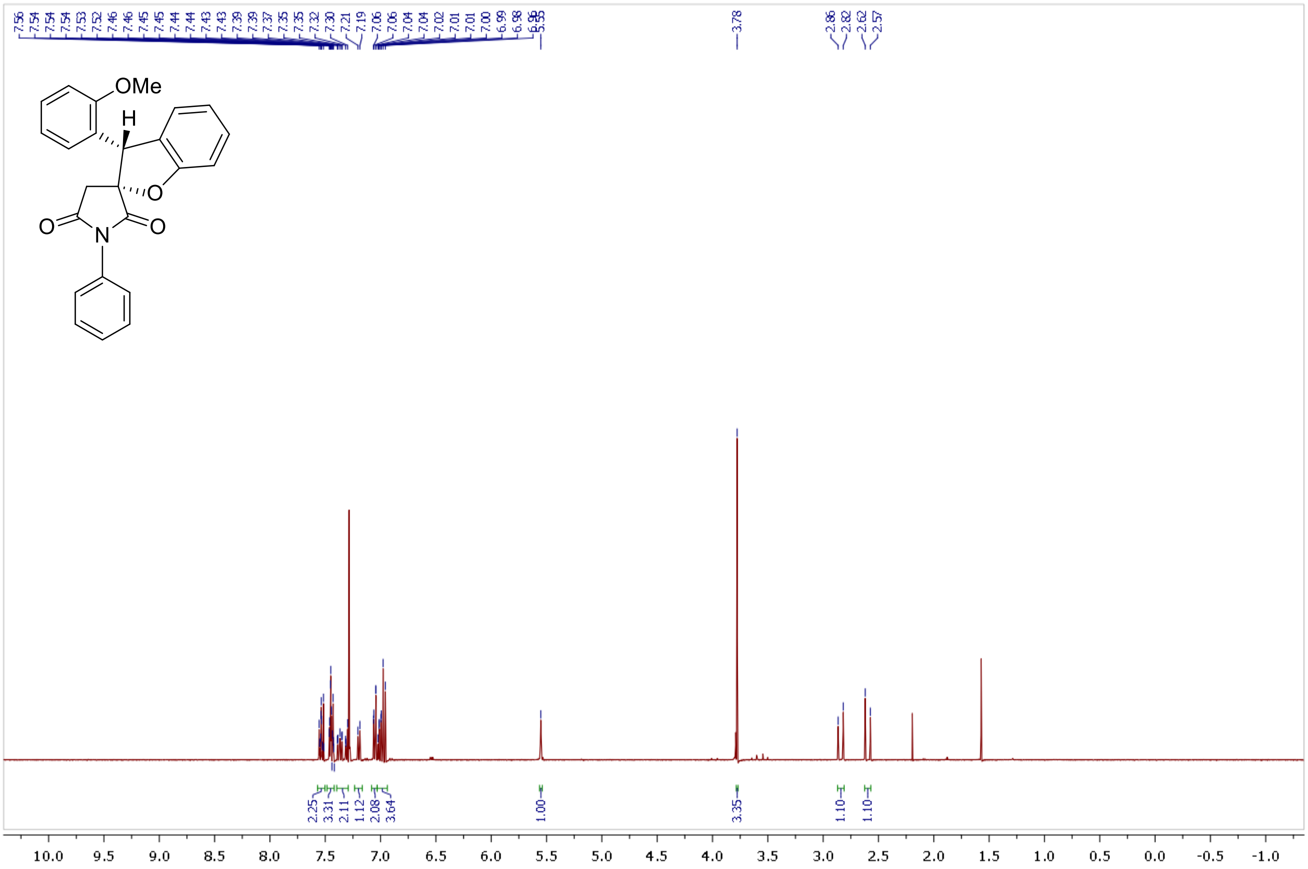

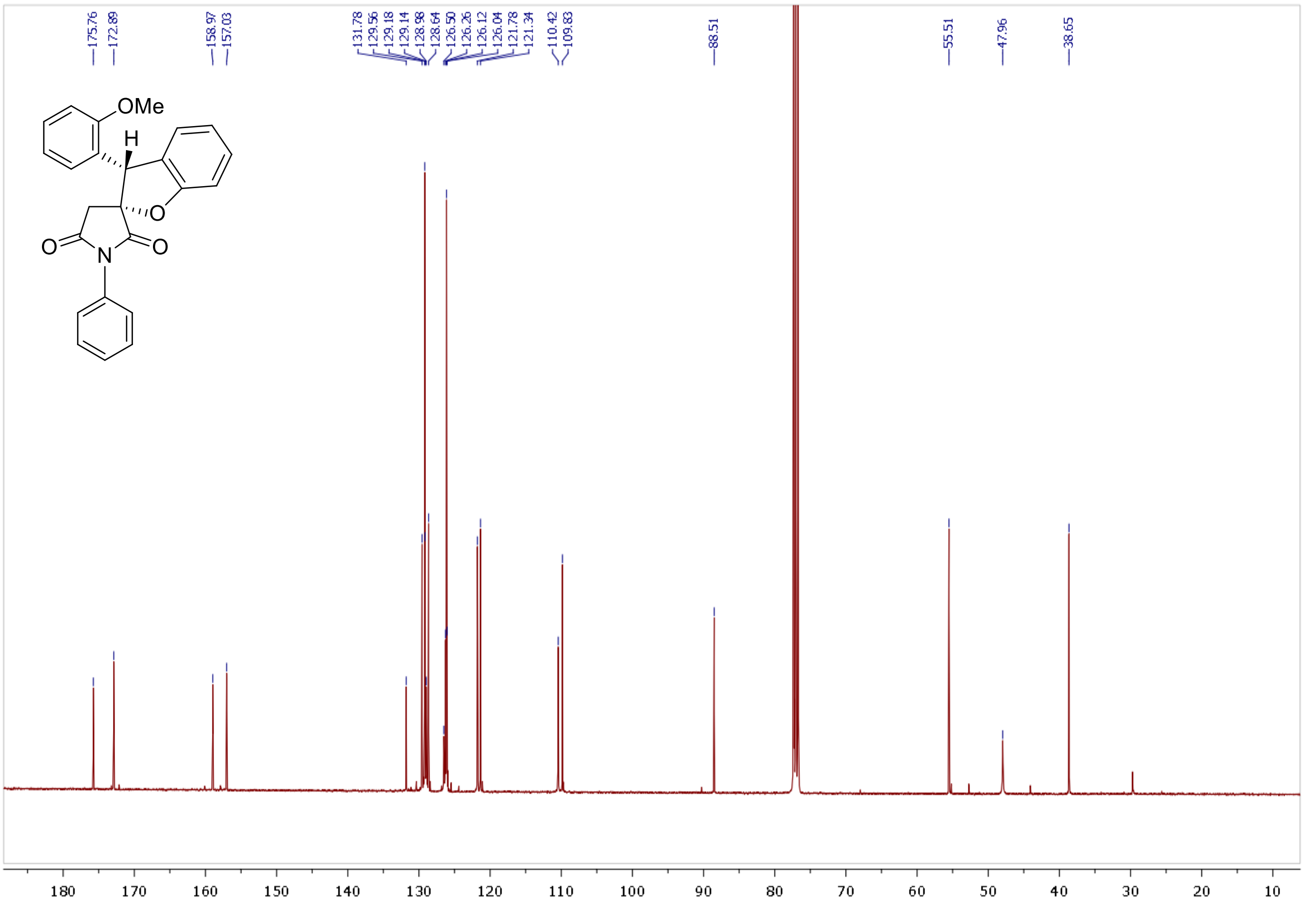

IV. X-ray crystallographic data for compound *syn*-7a

X-ray Single Crystal analysis was performed on RigakuXtaLAB. The crystal was grown by slow evaporation of solution in *n*-hexane-acetone mixture (1:1). The crystal was kept at 100.15 K during data collection. Using Olex2 [3], the structure was solved with the SHELXS [4] structure solution program using Direct Methods and refined with the SHELXL [5] refinement package using Least Squares minimization. CCDC 2166113 contain the supplementary crystallographic data for this paper. These data can be obtained free of charge from The Cambridge Crystallographic Data Centre via https://www.ccdc.cam.ac.uk/.

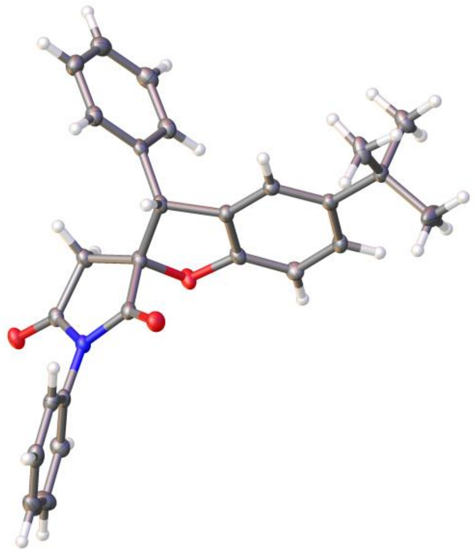

Figure S1. ORTEP representation of compound *syn*-**7a** (thermal ellipsoids are shown at 50% probability)

**Table S1.** Crystal data and structure refinement for *syn*-**7a**	
CCDC	2166113
Empiricalformula	$C_{27}H_{25}NO_3$
Formula weight	411.48
Temperature/K	100.15
Crystal system	monoclinic
Space group	$P2_1/c$
a/Å	9.8232(2)
b/Å	6.11690(10)
c/Å	35.3601(7)
α/°	90
β/°	97.266(2)
γ/°	90
Volume/Å^3	2107.64(7)
Z	4
ρ_{calc}g/cm^3	1.297
μ/mm^{-1}	0.670
F(000)	872.0

Crystal size/mm^3	0.08 × 0.04 × 0.03
Radiation	CuKα (λ = 1.54184)
2Θ range for data collection/°	9.076 to 152.92
Index ranges	-11 ≤ h ≤ 12, -7 ≤ k ≤ 4, -44 ≤ l ≤ 43
Reflections collected	17376
Independent reflections	4379 [R_{int} = 0.0259, R_{sigma} = 0.0178]
Data/restraints/parameters	4379/0/283
Goodness-of-fit on F^2	1.048
Final R indexes [I>=2σ (I)]	R_1 = 0.0418, wR_2 = 0.1024
Final R indexes [all data]	R1 = 0.0448, wR2 = 0.1047
Largest diff. peak/hole / e Å^{-3}	0.37/-0.26

V. References

1. Chupakhin, E. G.; Kantin, G. P.; Dar'in, D. V.; Krasavin, M. Convenient preparation of (*E*)-3-arylidene-4-diazopyrrolidine-2,5-diones in array format. *Mendeleev Commun.* **2021**, *31*, 36.
2. Chupakhin, E.; Gecht, M.; Ivanov, A.; Kantin, G.; Dar'in, D.; Krasavin, M. (*E*)-3-Arylidene-4-diazopyrrolidine-2,5-diones: Preparation and Use in RhII-Catalyzed X–H Insertion Reactions towards Novel, Medicinally Important Michael Acceptors. *Synthesis* **2021**, *53*, 1292.
3. Dolomanov, O.V., Bourhis, L.J., Gildea, R.J, Howard, J.A.K. & Puschmann, H. (2009), J. Appl. Cryst. 42, 339-341.
4. Sheldrick, G.M. (2008). Acta Cryst. A64, 112-122.
5. Sheldrick, G.M. (2015). Acta Cryst. C71, 3-8.
